# Small Structural Changes in Chili-Derived Capsaicin Resulting in Nonivamide Analogs of Significantly Improved Cytotoxicity and Good Tumor/Non-Tumor Cell Selectivity

**DOI:** 10.3390/molecules30173488

**Published:** 2025-08-25

**Authors:** Niels V. Heise, René Csuk, Thomas Mueller

**Affiliations:** 1 Organic Chemistry, Martin-Luther-University Halle-Wittenberg, Kurt-Mothes Str. 2, 06120 Halle (Saale), Germany; 2 Hematology/Oncology, Medical Faculty, University Clinic for Internal Medicine IV, Martin-Luther University Halle-Wittenberg, Ernst Grube Str. 40, 06120 Halle (Saale), Germany; thomas.mueller@medizin.uni-halle.de

**Keywords:** capsaicin, nonivamide, cytotoxicity

## Abstract

Capsaicin, the major pungent alkaloid in *Capsicum* species, has been reported to exhibit cytotoxic activity through various mechanisms. In this study, capsaicin and 37 structurally related vanillylamide and ester analogs were synthesized and evaluated for cytotoxic activity and tumor cell/non-tumor cell selectivity in vitro and compared with a *Capsicum baccatum* (*Aji mochero*) extract. Seven analogs with superior potency and selectivity compared to capsaicin were identified. Notably, vanillylamides with a C_16_–C_18_ chain exhibited IC_50_ values five-fold lower than capsaicin (15–84 µM), with selectivity indices up to 35. The extract obtained from the dried chili fruit, known to hold capsaicin as its primary component, however, exhibited significantly lower cytotoxic activity against tumor cells than pure capsaicin. These data demonstrate that even minor modifications to the acyl chain (as exemplified for the nonivamide analogs) can enhance the cytotoxicity and selectivity of these derivatives and that isolated compounds are able to offer even greater efficacy than whole-fruit extracts.

## 1. Introduction

Peppers are native to Central and South America and Mexico, with their use as spices dating back more than 6000 years [1]. It was C. Columbus who introduced chilis to Spain in 1493 [2], and in the following years, these spices spread all over Europe, and later on to India, Asia, and Africa.

**Figure 1:**
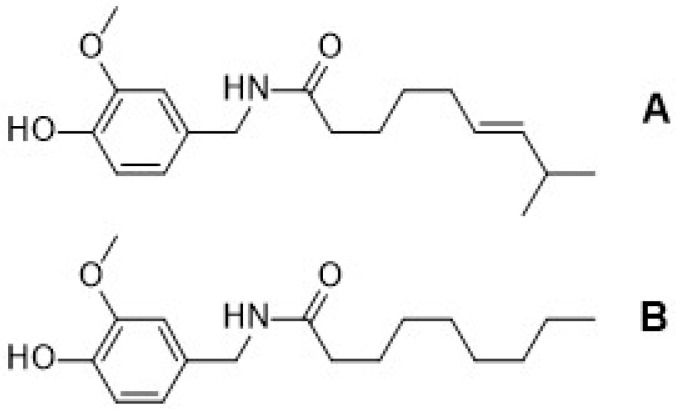
Structure of capsaicin (**A**) and nonivamide (**B**).

Plants of the genus *Capsicum* are rich in a class of compounds known as capsaicinoids, with capsaicin (**A**, 8-methyl-*N*-vanillyl-6-nonenamide, Figure 1) being the most important. The “chemistry” of **A** dates back to 1816: C.F. Buchholz [3] extracted **A** in impure form for the first time, and **A** was obtained in almost pure form by J.C. Tresh in 1876 [4]. Its burning and even painful sensations were first reported by R. Buchheim [5,6] and E. Högyes [7] in 1872 and 1877, respectively. Finally, the structure of **A** was elucidated by D.J. Bennett and G.W. Kirby almost a century later, in 1968 [8].

These sometimes extremely painful sensations are due to the binding of **A** and analogs thereof to the vanilloid receptor subtype 1 (TRPV1) [9], resulting in a hypersensitivity reaction in the skin.

For many years, **A** has been reported as a cytotoxic agent for more than 70 different tumor cell lines (e.g., carcinomas, sarcomas, leukemias, and lymphomas) [10,11] and several different modes of action have been reported, more or less irrespective of the presence of the TRPV1 receptor in these malignant cells [12,13,14,15].

However, prolonged exposure to low concentrations of **A** (0.1–10 μM) has been reported to lead to the development of aggressive phenotypes in tumor cells [10,16]. There is a suggestion that low concentrations might act as co-carcinogens [17], which has been supported by epidemiological studies revealing a positive correlation between chili consumption and the development of certain malignancies [18,19,20], but contradicted by recent results that did not find an increase in tumor incidence [21].

There have been several excellent reviews on this topic (also referred to in the literature as “the double-edged sword“ postulate) [11,22,23,24]. In addition, the role of the amide bond in capsaicin and analogs has been studied quite recently [25].

Cancer remains one of the leading causes of morbidity and mortality worldwide [26]. While chemotherapy is a cornerstone of cancer treatment, many conventional chemotherapeutic agents suffer from significant limitations, including severe side effects, limited tumor selectivity, and the emergence of drug resistance [27]. There is therefore an urgent need to discover and develop new anticancer compounds that are more effective, less toxic to normal cells, and capable of overcoming resistance mechanisms. Natural products and their derivatives continue to be a valuable source of novel chemical structures with potential anticancer activity, offering promising avenues for the development of improved therapeutics.

Recently, we reported on the strong inhibitory activity of capsaicin (**A**) and nonivamide (**B**, Figure 1) against acetylcholinesterase and butyrylcholinesterase [28]. Furthermore, **B** (also known as pseudocapsaicin) was shown to protect model cells from Alzheimer’s disease-related damage [23,29]. In this study, we characterized novel capsaicin-derivative compounds in comparison to capsaicin and whole-chili extract, focusing on their anticancer activity and selectivity. A mandatory prerequisite for the application of such compounds as potential anticancer agents is proof of low (cyto)toxicity to normal, non-malignant cells.

To accomplish this, we performed cytotoxicity assays to analyze the impact of new compounds on tumor cells and normal fibroblasts and to assess general cytotoxic activity and tumor cell selectivity. For this purpose, we used our standard cell line panel comprising tumor cell lines from various sources—including breast, lung, and colorectal cancers—representing the most common cancer types. As a result, we identified three compounds that showed cytotoxic activity in various tumor models while being non-toxic to normal fibroblasts. Their selectivity indices were up to 35, thereby outperforming capsaicin and whole-chili extract.

## 2. Results

### 2.1. Synthesis

**Scheme 1:**
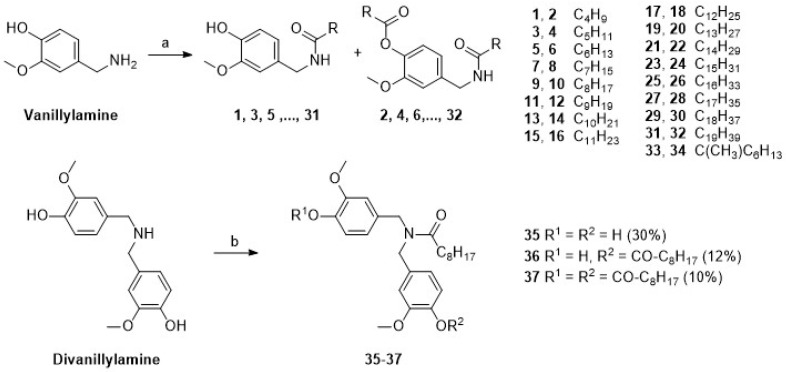
Synthesis of compounds **1**–**37**; reactions and conditions: (**a**) DCM, 5 h, 20 °C; (**b**) nonanoic acid, (COCl)_2_, DMF (cat.), and then divanillylamine, 5 h, 20 °C.

To investigate the activity/selectivity pattern in more detail, compounds **1**–**37** were prepared as previously reported [28] and—together with capsaicin—subjected to cytotoxicity assays. The data were compared with the characteristics of a prepared whole-chili extract, known to have capsaicin as its primary component. Capsaicin (**A**) was obtained from a local supplier and purified by chromatography [30] followed by repeated re-crystallization. For the synthesis of **1**–**34**, briefly, a panel of alkanoic acids were converted into their respective vanillyl amides (Scheme 1) and di-acylation products; products **35**–**37** were obtained from divanillylamine.

### 2.2. Biological Evaluation

**Figure 2:**
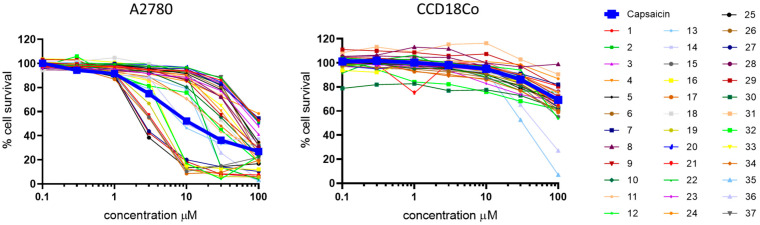
Dose–response pattern of new compounds (colored lines) compared to capsaicin (bold blue line) in A2780 cells and non-malignant fibroblasts.

All compounds (**1**–**37**) and capsaicin were screened in SRB assays for their cytotoxic activity. For an initial screening, A2780 (human ovarian carcinoma) cells were used, with CCD18Co (non-malignant human fibroblast) cells to obtain some indication about the general cytotoxic activity and the tumor/non-tumor-cell selectivity. The results from these assays are depicted in Figure 2.

**Figure 3:**
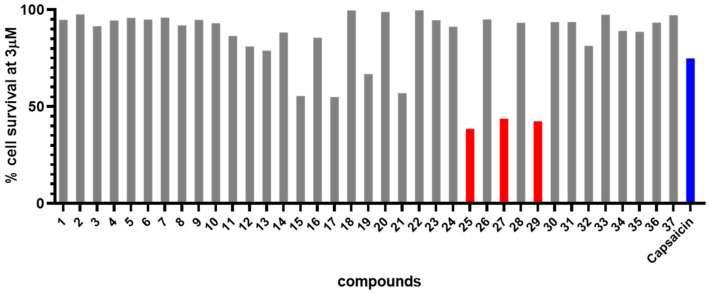
Analysis of cytotoxic activity of capsaicin (blue) and compounds **1**–**37** employing a concentration of 3 μM and A2780 tumor cells. Most active and selective compounds (**25**, **27**, **29**) are depicted in red color.

A potential tumor cell selectivity of compounds can be assumed if the dose–response pattern clearly differs between tumor and normal cells. Most compounds exhibited no toxicity and caused only minimal growth inhibition in normal fibroblasts, even at 100 µM, thus not reaching an IC_50_ value (Figure 2, right). As a result, 7 out of 37 compounds (**15**, **17**, **19**, **21**, **25**, **27**, **29**), with IC_50_ values well below 10 µM, demonstrated tumor cell selectivity over non-tumor cells and were more active than capsaicin. An analysis (Figure 3) of cytotoxic activity at 3 μM (from dose–response curves) revealed the three most active and selective compounds (red) compared to capsaicin (blue).

The three most active compounds (**25**, **27**, **29**) were further investigated concerning their cytotoxic activity using a panel of five different human tumor cell lines compared to non-malignant fibroblasts. The results from these investigations are summarized in Table 1.

These results confirm the superior activity/selectivity pattern of the new compounds compared to capsaicin. Overall, A2780 ovarian carcinoma and MCF7 breast carcinoma cells proved to be the most susceptible tumor cell types to capsaicin and new derivatives, with A2780 being the most sensitive.

**Figure 4:**
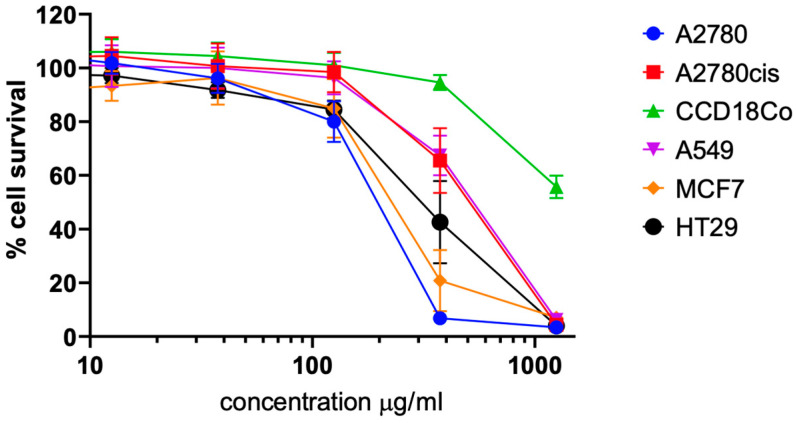
Analysis of the cytotoxic activity (from SRM, *n* = 3; means shown and standard deviation) of a chili extract in a panel of cancer cell lines from different entities and non-malignant fibroblasts. Chart shows mean curves from 3 independent experiments ± standard deviation. Cytotoxicity testing was performed in the concentration range of 0.125–1250 μg/mL; the area of cytotoxic activity is shown.

To complement our investigations, we were also interested in the cytotoxic effect of whole-chili extract given that capsaicin is its main component. Ultrasonic extraction of capsaicinoids from hot chili peppers was thereby shown to significantly improve the extraction yield since power ultrasound promotes cell wall disruption and hydration by forcing the solvent into the plant cells. This results in an increased mass transfer, where intracellular substances are washed out of the plant cell into the solvent. As a purely mechanical, non-thermal processing method, ultrasonic extraction prevents thermal damage to thermally labile compounds. Recently, Olguín-Rojas et al. [31] reported the extraction and isolation of capsaicinoids with a yield of more than 96% from three *Capsicum chinense* varieties. As a consequence, we decided to use a modified version of this method for the extract preparation, and *Aji mochero* (*Capsicum baccatum*) chilis were therefore used. Chili extracts are used as an ingredient in what is considered one of the most delicious extract-based sauces (“vicious but delicious”) in food production. However, the extract is also promoted as an appetite suppressant and as a weight loss agent through increased fat burning. We were therefore interested in determining the cytotoxic activity and selectivity of the extract. The results from these investigations are summarized in Table 2 and graphically illustrated in Figure 4.

As a result, the extract exhibited a cytotoxicity and selectivity pattern similar to that of capsaicin and the new derivatives, with the most pronounced effect observed in A2780 ovarian carcinoma and MCF7 breast carcinoma cells. Overall, there was a tumor cell selectivity over non-tumor cells, with a selectivity index (IC_50_ CCD18Co/IC_50_ A2780) of more than six-fold, which was very similar to that of capsaicin.

Interestingly, these results also revealed that the extract (with a capsaicin content of approximately 80%) [32,33,34] exhibited significantly lower cytotoxic activity against tumor cells than pure capsaicin (Table 2). In A2780 cells, for example, there was an approximately 30-fold difference between the IC_50_ values of capsaicin (15.9 µM ≈ 4.856 µg/mL) and the extract (80% of 183.4 µg/mL ≈ 146.72 µg/mL).

Finally, these results also suggest that even minor structural modifications (cf. compounds **25**, **27**, **29** compared to all other structures in this study) can lead to significant changes in their biological activity—for example, increased cytotoxic activity against tumor cells was observed accompanied by improved tumor cell/non-tumor cell selectivity.

Different modes of action for capsaicin have been reported for various cells. Our current investigations do not allow us to draw conclusions about how the derivatives exert their cytotoxic effect. Since the target(s) of our compounds are unknown and will be the subject of future investigations, we cannot perform structure–activity analyses or corresponding molecular modeling calculations.

## 3. Discussion

Capsaicin, the major pungent alkaloid in *Capsicum* species, exhibits well-documented anticancer activity across various tumor models, mediated by multiple mechanisms. This comprehensive study investigated capsaicin alongside 37 structurally related vanillylamide and ester analogs synthesized to evaluate cytotoxicity and tumor selectivity in vitro. A *Capsicum baccatum* (*Aji mochero*) extract was assessed comparatively. Overall, 37 structurally related vanillylamide and ester derivatives (compounds **1**–**37**) were synthesized via the conversion of various alkanoic acids into their respective vanillyl amides, including di-acylation and divanillylamine derivatives. As a result, seven analogs demonstrated superior potency and selectivity relative to capsaicin, with vanillylamides featuring C16-C18 chains showing five-fold-lower IC_50_ values (15–84 µM) and selectivity indices (SI) up to 35. Whole-fruit extract, despite capsaicin being its major component, exhibited significantly lower cytotoxic activity than pure capsaicin. These findings highlight the impact of minor acyl chain modifications on cytotoxicity and selectivity, underlining the potential of purified compounds over extracts for therapeutic applications in oncology.

Compounds **25**, **27**, and **29** emerged as the most active and selective derivatives, exhibiting IC_50_ values in A2780 cells of 2.8 µM, 3.4 µM, and 3.0 µM, respectively, with selectivity indices exceeding 29, compared to capsaicin’s IC_50_ of 15.9 µM and SI of 6.3.

Despite the high activity of compounds **25**, **27**, and **29**, it would be very interesting to synthesize and study the cytotoxic activity of analogs of compounds **35**–**37**, i.e., divanillylamides holding C_16_–C_18_ carboxylic acid residues. However, it has to be noted that solubility issues were encountered even with monomeric vanillylamides carrying longer alkyl chains. Hence, it can assumed that these issues will increase with divanillylamines holding three long alkyl chains, thus making them unsuitable for in vitro testing.

This systematic evaluation underscores the following: Capsaicin and structurally related vanillylamide analogs exhibit significant cytotoxicity toward various cancer cell lines, with a high degree of selectivity. Structural modifications, particularly in acyl chain length, can dramatically enhance anticancer efficacy and selectivity. Whole-capsicum extracts, despite high capsaicin content, display lower cytotoxic activity, indicating the benefit of purified compound use in therapeutic approaches. Several factors might explain this discrepancy, including matrix effects due to the presence of other compounds (that might interfere), reduced purity, degradation, and binding to other extract constituents.

## 4. Materials and Methods

### 4.1. General

Reagents were purchased from commercial suppliers and used without further purification. The solvents were dried according to usual procedures. TLC was performed on silica gel (Macherey-Nagel, detection with UV absorption; Macherey-Nagel, Düren Germany). Melting points have been measured with a Büchi M-565 instrument (Büchi Labortechnik, Flawill, Switzerland). NMR spectra were recorded using VARIAN spectrometers (Varian Germany, Darmstadt, Germany) at 27 °C (δ given in ppm; *J* in Hz, typical experiments for assignments: ^13^C APT, HMBC, HSQC). ASAP-MS spectra were taken on an Advion (Advion, Ithaca, NY, USA) expression CMS-L with an ASAP/APCI Ion source (capillary voltage 150 V, capillary temperature 220 °C, and voltage of the ion source: 15 V; APCI source temperature 300 °C with 5 μA). TLC plates (SiO_2_, F_254_ from Macherey-Nagel) were impregnated with AgNO_3_ (15% followed by drying at 110 °C). AgNO_3_-impregnated silica gel was freshly prepared from silica gel (180 g, 0.040–0.063 mm, Merck, Darmstadt, Germany) and AgNO_3_ (22.5 g in 45 mL water) followed by drying at 110 °C for 1 h. *Aji mochero* chilis were obtained from a local supplier, and used as received.

### 4.2. Synthesis of the Amides **1**–**37**

Reactions of the carboxylic acid (1.25 eq, all obtained from a local supplier; used as received) in dry DCM (25 mL) with oxalyl chloride (4.0 eq.) in the presence of catalytic amounts of dry DMF at 22 °C for 4 h followed by the evaporation of all volatiles in vacuo gave an oily residue that was re-dissolved in dry DCM (25 mL) and added slowly to a solution of (di)-vanillylamide (1 eq., from a local supplier, used as received) in dry DCM (25 mL). The reactions were completed in about 5 h (as checked by TLC). Standard aqueous work-up followed by chromatography (silica gel, hexanes/ethyl acetate mixtures) gave the products. Identity with previously synthesized compounds [28] was determined by comparing their ^1^H and ^13^C NMR spectra, MS and IR spectra, and (where applicable) mixed melting points; the former were identical to authentic material, and the latter showed no melting point depression. Spectra and data are provided in the Supplementary Materials file. CAUTION: skin contact of capsaicin and analogs leads to extremely burning sensations.

#### 4.2.1. *N*-[(4-Hydroxy-3-methoxy)benzyl]pentanamide (**1**) and 2-methoxy-4-[(pentanoylamino)methyl]phenyl pentanoate (**2**)

Following GP from vanillylamine and pentanoic acid, **1** (25%) and **2** (28%) were obtained.

Data for **1**: colorless oil; R_F_ = 0.21 (silica gel, *n*-hexane/ethyl acetate, 6:4); ESI-MS (methanol/chloroform, 4:1): *m*/*z* (%) = 102 ([M + Na]^+^, 100%).

Data for **2**: R_F_ = 0.37 (silica gel, *n*-hexane/ethyl acetate, 6:4); ESI-MS (methanol/chloroform, 4:1): *m*/*z* (%) = 344 ([M + Na]^+^, 100%).

#### 4.2.2. *N*-[(4-Hydroxy-3-methoxy)benzyl]hexanamide (**3**) and 2-methoxy-4-[(hexanoylamino)methyl]phenyl hexanoate (**4**)

Following GP from vanillylamine and hexanoic acid, **3** (27%) and **4** (25%) were obtained.

Data for **3**: m.p. 49–49.5 °C; R_F_ = 0.06 (silica gel, *n*-hexane/ethyl acetate, 7:3); ESI-MS (methanol/chloroform, 4:1): *m*/*z* (%) = 274 ([M + Na]^+^, 100%).

Data for **4**: colorless solid; m.p. 104–106 °C; R_F_ = 0.22 (silica gel, *n*-hexane/ethyl acetate, 7:3); ESI-MS (methanol/chloroform, 4:1): *m*/*z* (%) = 372 ([M + Na]^+^, 100%).

#### 4.2.3. *N*-[(4-Hydroxy-3-methoxy)benzyl]heptanamide (**5**) and 2-methoxy-4-[(heptanoylamino)methyl]phenyl heptanoate (**6**)

Following GP from vanillylamine and heptanoic acid, **5** (31%) and **6** (56%) were obtained.

Data for **5**: m.p. 59–62 °C; R_F_ = 0.46 (silica gel, *n*-hexane/ethyl acetate, 6:4); ESI-MS (methanol/chloroform, 4:1): *m*/*z* (%) = 288 ([M + Na]^+^, 100%).

Data for **6**: colorless solid; m.p. 63–65 °C; R_F_ = 0.67 (silica gel, *n*-hexane/ethyl acetate, 6:4); ESI-MS (methanol/chloroform, 4:1): *m*/*z* (%) = 400 ([M + Na]^+^, 100%).

#### 4.2.4. *N*-[(4-Hydroxy-3-methoxy)benzyl]octanamide (**7**) and 2-methoxy-4-[(octanoylamino)methyl]phenyl octanoate (**8**)

Following GP from vanillylamine and octanoic anhydride, **7** (36%) and **8** (56%) were obtained.

Data for **7**: m.p. 41–44 °C; R_F_ = 0.44 (silica gel, *n*-hexane/ethyl acetate, 6:4); ESI-MS (methanol/chloroform, 4:1): *m*/*z* (%) = 304 ([M + Na]^+^, 100%).

Data for **8**: colorless solid; m.p. 71–73 °C; R_F_ = 0.88 (silica gel, *n*-hexane/ethyl acetate, 6:4); ESI-MS (methanol/chloroform, 4:1): *m*/*z* (%) = 428 ([M + Na]^+^, 100%).

#### 4.2.5. *N*-[(4-Hydroxy-3-methoxy)benzyl]nonanamide (**9**) and 2-methoxy-4-[(nonanoylamino)methyl]phenyl nonanoate (**10**)

Following GP from vanillylamine and nonanoic acid, **9** (67%) and **10** (26%) were obtained.

Data for **9**: colorless solid; m.p. 48–50 °C; R_F_ = 0.65 (silica gel, *n*-hexane/ethyl acetate, 4:6); ESI-MS (methanol/chloroform, 4:1): *m*/*z* (%) = 292 ([M − H]^−^, 100%).

Data for **10**: colorless solid; m.p. 74 °C; R_F_ = 0.8 (silica gel, *n*-hexane/ethyl acetate, 4:6); ESI-MS (methanol/chloroform, 4:1): *m*/*z* (%) = 432 ([M − H]^−^, 100%).

#### 4.2.6. *N*-[(4-Hydroxy-3-methoxy)benzyl]decanamide (**11**) and 2-methoxy-4-[(decanoylamino)methyl]phenyl decanoate (**12**)

Following GP from vanillylamine and decanoic acid, **11** (25%) and **12** (54%) were obtained.

Data for **11**: colorless solid; m.p. 55–57 °C; R_F_ = 0.16 (silica gel, *n*-hexane/ethyl acetate, 6:4); ESI-MS (methanol/chloroform, 4:1): *m*/*z* (%) = 331 ([M + Na]^+^, 100%).

Data for **12**: colorless solid; m.p. 99 °C; R_F_ = 0.70 (silica gel, *n*-hexane/ethyl acetate, 6:4); ESI-MS (methanol/chloroform, 4:1): *m*/*z* (%) = 460 ([M − H]^−^, 100%).

#### 4.2.7. *N*-[(4-Hydroxy-3-methoxy)benzyl]undecanamide (**13**) and 22-methoxy-4-[(undecanoylamino)methyl]phenyl undecanoate (**14**)

Following GP from vanillylamine and undecanoic acid, **13** (23%) and **14** (54%) were obtained.

Data for **13**: colorless solid; m.p. 60–63 °C; R_F_ = 0.24 (silica gel, *n*-hexane/ethyl acetate, 6:4); ESI-MS (methanol/chloroform, 4:1): *m*/*z* (%) = 344 ([M + Na]^+^, 100%).

Data for **14**: colorless solid; m.p. 81–83 °C; R_F_ = 0.64 (silica gel, *n*-hexane/ethyl acetate, 6:4); ESI-MS (methanol/chloroform, 4:1): *m*/*z* (%) = 513 ([M + Na]^+^, 100%).

#### 4.2.8. *N*-[(4-Hydroxy-3-methoxy)benzyl]dodecanamide (**15**) and 2-methoxy-4-[(dodecanoylamino)methyl]phenyl dodecanoate (**16**)

Following GP from vanillylamine and dodecanoic acid, **15** (38%) and **16** (59%) were obtained.

Data for **15** colorless solid; m.p. 76–78 °C; R_F_ = 0.25 (silica gel, *n*-hexane/ethyl acetate, 6:4); ESI-MS (methanol/chloroform, 4:1): *m*/*z* (%) = 358 ([M + Na]^+^, 100%).

Data for **16**: colorless solid; m.p. 84 °C; R_F_ = 0.64 (silica gel, *n*-hexane/ethyl acetate, 6:4); ESI-MS (methanol/chloroform, 4:1): *m*/*z* (%) = 540 ([M + Na]^+^, 100%).

#### 4.2.9. *N*-[(4-Hydroxy-3-methoxy)benzyl]tridecanamide (**17**) and 2-methoxy-4-[(tridecanoylamino)methyl]phenyl tridecanoate (**18**)

Following GP from vanillylamine and tridecanoic acid, **17** (43%) and **18** (33%) were obtained.

Data for **17**: colorless solid; m.p. 70–72 °C; R_F_ = 0.11 (silica gel, *n*-hexane/ethyl acetate, 7:3); ESI-MS (methanol/chloroform, 4:1): *m*/*z* (%) = 373 ([M + Na]^+^, 100%).

Data for **18**: m.p. 79 °C; R_F_ = 0.24 (silica gel, *n*-hexane/ethyl acetate, 7:3); ESI-MS (methanol/chloroform, 4:1): *m*/*z* (%) = 569 ([M + Na]^+^, 100%).

#### 4.2.10. *N*-[(4-Hydroxy-3-methoxy)benzyl]tetradecanamide (**19**) and 2-methoxy-4-[(tetradecanoylamino)methyl]phenyl tetradecanoate (**20**)

Following GP from vanillylamine and tetradecanoic acid, **19** (33%) and **20** (47%) were obtained.

Data for **19**: colorless solid; m.p. 76 °C; R_F_ = 0.13 (silica gel, *n*-hexane/ethyl acetate, 7:3); ESI-MS (methanol/chloroform, 4:1): *m*/*z* (%) = 386 ([M + Na]^+^, 100%).

Data for **20**: colorless solid; m.p. 85–87 °C; R_F_ = 0.28 (silica gel, *n*-hexane/ethyl acetate, 7:3); ESI-MS (methanol/chloroform, 4:1): *m*/*z* (%) = 596 ([M + Na]^+^, 100%).

#### 4.2.11. *N*-[(4-Hydroxy-3-methoxy)benzyl]pentadecanamide (**21**) and 2-methoxy-4-[(pentadecanoylamino)methyl]phenyl pentadecanoate (**22**)

Following GP from vanillylamine and pentadecanoic acid, **21** (48%) and **22** (26%) were obtained.

Data for **21**: colorless solid; m.p. 79 °C; R_F_ = 0.11 (silica gel, *n*-hexane/ethyl acetate, 7:3); ESI-MS (methanol/chloroform, 4:1): *m*/*z* (%) = 400 ([M + Na]^+^, 100%).

Data for **22**: colorless solid; m.p. 79–80 °C; R_F_ = 0.75 (silica gel, *n*-hexane/ethyl acetate, 6:4); ESI-MS (methanol/chloroform, 4:1): *m*/*z* (%) = 625 ([M + Na]^+^, 100%).

#### 4.2.12. *N*-[(4-Hydroxy-3-methoxy)benzyl]hexadecanamide (**23**) and 2-methoxy-4-[(hexadecanoylamino)methyl]phenyl hexadecanoate (**24**)

Following GP from vanillylamine and hexadecanoic acid, **23** (31%) and **24** (30%) were obtained.

Data for **23**: colorless solid; m.p. 80 °C; R_F_ = 0.12 (silica gel, *n*-hexane/ethyl acetate, 7:3); ESI-MS (methanol/chloroform, 4:1): *m*/*z* (%) = 390 ([M − H]^−^, 100%).

Data for **24**: m.p. 91–92 °C; R_F_ = 0.45 (silica gel, *n*-hexane/ethyl acetate, 7:3); ESI-MS (methanol/chloroform, 4:1): *m*/*z* (%) = 665 ([M + Na]^+^, 100%).

#### 4.2.13. *N*-[(4-Hydroxy-3-methoxy)benzyl]heptadecanamide (**25**) and 2-methoxy-4-[(heptadecanoylamino)methyl]phenyl heptadecanoate (**26**)

Following GP from vanillylamine and heptadecanoic acid, **25** (32%) and **26** (30%) were obtained.

Data for **25**: colorless solid; m.p. 84 °C; R_F_ = 0.15 (silica gel, *n*-hexane/ethyl acetate, 7:3); ESI-MS (methanol/chloroform, 4:1): *m*/*z* (%) = 404 ([M − H]^−^, 100%).

Data for **26**: m.p. 94 °C; R_F_ = 0.45 (silica gel, *n*-hexane/ethyl acetate, 7:3); ESI-MS (methanol/chloroform, 4:1): *m*/*z* (%) = 680 ([M + Na]^+^, 100%).

#### 4.2.14. *N*-[(4-Hydroxy-3-methoxy)benzyl]octadecanamide (**27**) and 2-methoxy-4-[(octadecanoylamino)methyl]phenyl octadecanoate (**28**)

Following GP from vanillylamine and octadecanoic acid, **27** (45%) and **28** (34%) were obtained.

Data for **27**: colorless solid; m.p. 87–89 °C; R_F_ = 0.10 (silica gel, *n*-hexane/ethyl acetate, 7:3); ESI-MS (methanol/chloroform, 4:1): *m*/*z* (%) = 418 ([M − H]^−^, 100%).

Data for **28**: colorless solid; 94 °C; R_F_ = 0.49 (silica gel, *n*-hexane/ethyl acetate, 7:3); ESI-MS (methanol/chloroform, 4:1): *m*/*z* (%) = 709 ([M + Na]^+^, 100%).

#### 4.2.15. *N*-[(4-Hydroxy-3-methoxy)benzyl]nonadecanamide (**29**) and 2-methoxy-4-[(nonadecanoylamino)methyl]phenyl nonadecanoate (**30**)

Following GP from vanillylamine and nonadecanoic acid, **29** (51%) and **30** (34%) were obtained.

Data for **29**: colorless solid; m.p. 92 °C; R_F_ = 0.18 (silica gel, *n*-hexane/ethyl acetate, 7:3); ESI-MS (methanol/chloroform, 4:1): *m*/*z* (%) = 432 ([M − H]^−^, 100%).

Data for **30**: colorless solid; m.p. 97 °C; R_F_ = 0.55 (silica gel, *n*-hexane/ethyl acetate, 7:3); ESI-MS (methanol/chloroform, 4:1): *m*/*z* (%) = 737 ([M + Na]^+^, 100%).

#### 4.2.16. *N*-[(4-Hydroxy-3-methoxy)benzyl]icosanamide (**31**) and 2-methoxy-4-[(icosanoylamino)methyl]phenyl icosanoate (**32**)

Following GP from vanillylamine and icosanoic acid, **31** (20%) and **32** (56%) were obtained.

Data for **31**: colorless solid; m.p. 94 °C; R_F_ = 0.38 (silica gel, *n*-hexane/ethyl acetate, 6:4); ESI-MS (methanol/chloroform, 4:1): *m*/*z* (%) = 446 ([M − H^]−,^ 100%).

Data for **32**: colorless solid; m.p. 80 °C; R_F_ = 0.75 (silica gel, *n*-hexane/ethyl acetate, 6:4); ESI-MS (methanol/chloroform, 4:1): *m*/*z* (%) = 764 ([M + Na]^+^, 100%).

#### 4.2.17. *N*,*N*-bis(4-hydroxy-3-methoxybenzyl)nonanamide (**33**), 4-{[4-hydroxy-3-methoxybenzyl)(nonanoyl)amino]methyl}-2-methoxyphenyl nonanoate (**34**) and (nonanoylimino) bis(methylene-2-methoxy-4,1-phenylene) dinonanoate (**35**)

Following GP from divanillylamine and nonanoic acid, **33** (30%), **34** (12%), and **35** (10%) were obtained.

Data for **33**: colorless oil; R_F_ = 0.55 (*n*-hexane/ethyl acetate, 5:5); ESI-MS (methanol/chloroform, 4:1): *m*/*z* (%) = 430 ([M + H]^+^, 100%).

Data for **34**: colorless oil; R_F_ = 0.65 (*n*-hexane/ethyl acetate, 5:5); ESI-MS (methanol/chloroform, 4:1): *m*/*z* (%) = 592 ([M + Na]^+^, 100%).

Data for **35**: colorless oil; R_F_ = 0.8 (*n*-hexane/ethyl acetate, 5:5); ESI-MS (methanol/chloroform, 4:1): *m*/*z* (%) = 733 ([M + Na]^+^, 100%).

#### 4.2.18. *N*-[(4-Hydroxy-3-methoxyphenyl)methyl]-2-methyloctanamide (**36**) and 4-{[(2-methyloctanoyl)amino]methyl}-2-(methyloxy)phenyl 2-methyloctanoate (**37**)

Following GP from vanillylamine and 2-methyloctanoic acid, **36** (48%) and **37** (17%) were obtained.

Data for **36**: colorless solid; m.p. 78–80 °C; R_F_ = 0.28 (silica gel, *n*-hexane/ethyl acetate, 4:6); ESI-MS (methanol/chloroform, 4:1): *m*/*z* (%) = 292 ([M − H]^−^, 100%).

Data for **37**: colorless solid; m.p. 80–82 °C; R_F_ = 0.55 (silica gel, *n*-hexane/ethyl acetate, 4:6); ESI-MS (methanol/chloroform, 4:1): *m*/*z* (%) = 432 ([M − H]^−^, 100%).

### 4.3. Capsaicin

Capsaicin was obtained from a local supplier and purified [30] to remove traces of small amounts of main/secondary capsaicinoids. Chromatography was performed on AgNO_3_-impregnated silica gel using dry chloroform as the eluent (R_f_ = 0.03) followed by a first crystallization from 2-propanol/*n*-hexane (15:85) at −40 °C followed by a second re-crystallization from methyl-tert-butylether/*n*-pentane (2:1) at −40 °C to yield colorless crystals; m.p. 67–69 °C (lit.: [35] 58–61 °C); NMR and MS as reported by Drosky et al. [30].

### 4.4. Preparation of the Chili Extract

The chili fruit (*Capsicum baccatum*, obtained from a local market, 14 g) were cut in small pieces and soaked in methanol for 1 h; all of the material was transferred into an ultrasound device (Hielscher UP200S, Hielscher Ultrasonics GmbH, Teltow, Germany) and treated at 50 °C for 10 min (25 kHz, 50–560 W). After cooling to 22 °C, the reaction mixture was filtered, and the solvent was evaporated under diminished pressure, leading to a brownish amorphous powder (160 mg) that was used in the bio-assays.

### 4.5. SRB Assay

Cytotoxic activities of compounds were analyzed using the SRB cytotoxicity assay. The human cancer cell lines A2780 (ECACC #93112519), A2780Cis (ECACC #93112517), A549 (ATCC—CCL-185), HT29 (ATCC—HTB-38), and MCF7 (ATCC—HTB-22) were cultivated in RPMI1640 medium, non-malignant human fibroblasts CCD18Co (ATCC—CRL-1459) were grown in MEME (both from Sigma-Aldrich, St. Louis, MO, USA). Both media were supplemented with 10% fetal bovine serum (Biowest, Nuaillé, France) and 1% penicillin-streptomycin (Sigma-Aldrich). Cells were seeded in 96-well plates and, after 24h, were treated with serial dilutions of compounds for 72 h. All subsequent steps were performed according to the previously described SRB assay protocol [26,36,37]. Dose–response curves and calculations of IC_50_ values, including standard deviations, were performed using GraphPad Prism (version 8) (https://www.graphpad.com, accessed on 17 March 2025).

## Figures and Tables

**Table 1:** Cytotoxic activity (IC_50_, means with SD ≤ 15%, in μM) from SRB assays; three independent experiments of the three most active compounds—**25**, **27**, and **29**—and capsaicin (**A**) in a panel of human cancer cell lines: A2780, A2780cis (ovarian carcinoma), A549 (lung carcinoma), MCF7 (breast carcinoma), HT29 (colorectal carcinoma), and CCD18Co (non-malignant human fibroblasts). A selectivity index (SI, as a ration of the respective IC_50_ values (IC_50_ CCD18Co/IC_50_ A2780) is provided too.

Comp.	A2780	A2780cis	A549	MCF7	HT29	CCD18Co	SI
**25**	2.8	>100	60.5	38.4	78.5	>100	>35.7
**27**	3.4	76.4	50.3	32.6	42.4	>100	>29.0
**29**	3.0	59.5	55.3	15.9	68.4	>100	>33.4
**A**	15.9	84.3	70.3	42.1	51.0	>100	>6.3

**Table 2:** Cytotoxicity of a chili extract and of capsaicin (**A**) in a panel of different cancer cell lines and non-malignant CCD18Co cells. IC_50_ values of the extract (μg/mL) were calculated from dose–response curves shown in Figure 4 (three independent experiments); IC_50_ values for capsaicin are taken from Table 1 and were converted into μg/mL.

Cell Line	Extract (μg/mL)	Capsaicin A (μg/mL)
A2780	183.4	4.9
A2780cis	460.9	25.8
A549	484.5	21.5
MCF7	232.4	12.9
HT29	309.9	15.6
CCD18Co	>1250	>30.5

## Data Availability

The raw data supporting the conclusions of this article will be made available by the authors upon request.

## Supplementary Materials

The following supporting information can be downloaded at: https://www.mdpi.com/article/10.3390/molecules30173488/s1. Spectra and data for compounds, ^1^H and ^13^C NMR spectra of compounds **1**–**37**.
