# Supplementary material for: Small Structural Changes in Chili-Derived Capsaicin Resulting in Nonivamide Analogs of Significantly Improved Cytotoxicity and Good Tumor/Non-Tumor Cell Selectivity
Source: Molecules. 2025 Aug 25;30(17):3488. doi: 10.3390/molecules30173488 (PMC12429925; doi:10.3390/molecules30173488)

Supplementary Materials

Small Structural Changes in Chili-Derived Capsaicin Resulting in Nonivamide Analogs of Significantly Improved Cytotoxicity and Good Tumor/Non-Tumor Cell Selectivity

Niels V. Heise [1], René Csuk [1,*] and Thomas Mueller [2]

[1] Organic Chemistry, Martin-Luther-University Halle-Wittenberg, Kurt-Mothes Str. 2, 06120 Halle (Saale), Germany
[2] Hematology/Oncology, Medical Faculty, University Clinic for Internal Medicine IV, Martin-Luther University Halle-Wittenberg, Ernst Grube Str. 40, 06120 Halle (Saale), Germany
* Correspondence: rene.csuk@chemie.uni-halle.de

1. Data for compounds **1-37**.

1.1.*N*-[(4-Hydroxy-3-methoxy)benzyl]pentanamide (**1**)

Colorless oil; R_F = 0.21 (silica gel, *n*-hexane/ethyl acetate, 6:4); ESI-MS (methanol/chloroform, 4:1): *m/z* (%) = 102 ($[M+Na]^+$, 100%); ^{1}H NMR: δ = 7.26 (*s*, 1H), 6.96 (*d*, *J* = 8.0 Hz, 1H), 6.88 (*d*, *J* = 1.9 Hz, 1H), 6.82 (*dd*, *J* = 8.1, 1.9 Hz, 1H), 5.89 (*s*, 1H, OH), 4.40 (*d*, *J* = 4.6 Hz, 2H), 3.80 (*s*, 3H), 2.25 – 2.20 (*t*, *J* = 7.5 Hz, 2H), 1.79 – 1.70 (*m*, 2H), 1.51 – 1.40 (*m*, 2H), 0.96 (*m*, 3H) ppm; ^{13}C NMR: δ = 13.7, 22.4, 27.8, 33.7, 43.4, 55.9, 112.2, 120.0, 122.9, 137.2, 139.2, 151.3, 171.9 ppm.

1.2. 2-Methoxy-4-[(pentanoylamino)methyl]phenyl pentanoate (**2**)

Colorless oil; R_F = 0.37 (silica gel, *n*-hexane/ethyl acetate, 6:4); ESI-MS (methanol/chloroform, 4:1): *m/z* (%) = 344 ($[M+Na]^+$, 100%); ^{1}H NMR: δ = 6.96 (*d*, *J* = 8.0 Hz, 1H), 6.89 (*d*, *J* = 1.9 Hz, 1H), 6.83 (*dd*, *J* = 8.0, 1.8 Hz, 1H), 5.79 (*s*, 1H), 4.40 (*d*, *J* = 5.1 Hz, 2H), 3.80 (*s*, 3H), 2.57 (*t*, *J* = 7.5 Hz, 2H), 2.22 (*t*, *J* = 7.6 Hz, 2H), 1.79 – 1.70 (*m*, 2H), 1.69 – 1.59 (*m*, 2H), 1.50 – 1.41 (*m*, 2H), 1.41 – 1.31 (*m*, 2H), 0.96 (*t*, *J* = 7.4 Hz, 3H), 0.92 (*t*, *J* = 7.3 Hz, 3H) ppm; ^{13}C NMR: δ = 13.7, 13.8, 22.2, 22.4, 27.0, 27.8, 33.7, 36.5, 43.5, 55.9, 112.2, 120.0, 122.9, 137.2, 139.2, 151.3, 171.9, 173.0 ppm.

1.3. *N*-[(4-Hydroxy-3-methoxy)benzyl]hexanamide (**3**)

White solid; m.p. 49-49.5 °C); R_F = 0.06 (silica gel, *n*-hexane/ethyl acetate, 7:3); ESI-MS (methanol/chloroform, 4:1): *m/z* (%) = 274 ($[M+Na]^+$, 100%); ^{1}H NMR: δ = 6.81 (*d*, *J* = 8.0

Hz, 1H), 6.75 (*d*, J = 1.9 Hz, 1H), 6.69 (*dd*, J = 8.0, 1.9 Hz, 1H), 6.10 (*s*, 1H), 4.29 (*d*, J = 5.5 Hz, 2H), 3.80 (*s*, 3H), 2.16 (*d*, J = 7.8 Hz, 2H), 1.66 – 1.57 (*m*, 2H), 1.32 – 1.20 (*m*, 4H), 0.85 (*d*, J = 6.9 Hz, 3H) ppm; ^{13}C NMR: δ = 13.9, 22.3, 25.5, 31.4, 36.6, 43.4, 55.8, 110.8, 114.5, 120.6, 130.2, 145.2, 146.9, 173.4 ppm.

1.4. 2-Methoxy-4-[(hexanoylamino)methyl]phenyl hexanoate (**4**)

Colorless solid; m.p. 104-106 °C; R_F = 0.22 (silica gel, *n*-hexane/ethyl acetate, 7:3); ESI-MS (methanol/chloroform, 4:1): *m/z* (%) = 372 ([M+Na]$^+$, 100%); ^{1}H NMR: δ = 6.84 (*d*, J = 8.0 Hz, 1H), 6.75 (*d*, J = 1.9 Hz, 1H), 6.72 (*dd*, J = 8.0, 1.9 Hz, 1H), 6.54 (*s*, 1H), 4.41 – 4.25 (*m*, 2H), 3.86 (*s*, 3H), 3.41 (*t*, J = 7.4 Hz, 2H), 2.56 – 2.49 (*m*, 2H), 1.89 – 1.76 (*m*, 2H), 1.59 – 1.49 (*m*, 2H), 1.36 – 1.18 (*m*, 8H)), 0.91 – 0.83 (*m*, 6H) ppm; ^{13}C NMR: δ = 13.7, 13.8, 22.4, 22.4, 22.9, 29.6, 30.8, 31.2, 42.8, 43.5, 55.9, 60.7, 110.4, 114.4, 120.6, 130.0, 145.1, 146.7, 168.6, 210.1 ppm.

1.5. *N*-[(4-Hydroxy-3-methoxy)benzyl]heptanamide (**5**)

Colorless solid; m.p. 59-62 °C); R_F = 0.46 (silica gel, *n*-hexane/ethyl acetate, 6:4); ESI-MS (methanol/chloroform, 4:1): *m/z* (%) = 288 ([M+Na]$^+$, 100%); ^{1}H NMR: δ = 6.86 (*d*, J = 8.0 Hz, 1H), 6.81 (*d*, J = 1.9 Hz, 1H), 6.76 (*dd*, J = 8.1, 1.9 Hz, 1H), 5.79 (*s*, 1H), 4.35 (*d*, J = 5.5 Hz, 2H), 3.87 (*s*, 3H), 2.20 (*t*, 2H), 1.69 – 1.61 (*m*, 2H), 1.37 – 1.23 (*m*, 6H), 0.87 (*t*, 3H) ppm; ^{13}C NMR: δ 14.0, 22.5, 25.8, 29.0, 31.5, 36.8, 43.5, 55.9, 110.7, 114.4, 120.8, 130.3, 145.2, 146.7, 173.0 ppm.

1.6. 2-Methoxy-4-[(heptanoylamino)methyl]phenyl heptanoate (**6**)

Colorless solid; m.p. 63-65 °C; R_F = 0.67 (silica gel, *n*-hexane/ethyl acetate, 6:4); ESI-MS (methanol/chloroform, 4:1): *m/z* (%) = 400 ([M+Na]$^+$, 100%); ^{1}H NMR: δ = 6.95 (*d*, J = 8.0 Hz, 1H), 6.88 (*d*, J = 1.9 Hz, 1H), 6.82 (*dd*, J = 8.0, 1.8 Hz, 1H), 5.91 (*s*, 1H), 4.39 (*d*, J = 5.0 Hz, 2H), 3.79 (*s*, 3H), 2.56 (*t*, J = 7.5 Hz, 2H), 2.21 (*t*, J = 7.6 Hz, 2H), 1.81 – 1.69 (*m*, 2H), 1.69 – 1.60 (*m*, 2H), 1.47 – 1.37 (*m*, 2H), 1.37 – 1.22 (*m*, 10H), 0.93 – 0.84 (*m*, 6H) ppm; ^{13}C NMR: δ = 14.0, 14.0, 22.5, 22.5, 25.0, 25.7, 28.7, 29.0, 31.4, 31.5, 34.0, 36.7, 43.4, 55.8, 112.2, 120.0, 122.8, 137.2, 139.2, 151.3, 172.0, 173.1 ppm.

1.7. *N*-[(4-Hydroxy-3-methoxy)benzyl]octanamide (**7**)

Data for **7**: m.p. 41-44 °C); R_F = 0.44 (silica gel, *n*-hexane/ethyl acetate, 6:4); ESI-MS (methanol/chloroform, 4:1): *m/z* (%) = 304 ($[M+Na]^+$, 100%); ^1H NMR: δ = 6.83 (*d*, *J* = 8.0 Hz, 1H), 6.78 (*d*, *J* = 1.9 Hz, 1H), 6.73 (*dd*, *J* = 8.0, 1.9 Hz, 1H), 5.95 (*s*, 1H), 4.32 (*d*, *J* = 5.3 Hz, 2H), 3.84 (*s*, 3H), 2.19 (*t*, 2H), 1.69 – 1.57 (*m*, 2H), 1.33 – 1.19 (*m*, 8H), 0.88 – 0.83 (*m*, 3H) ppm; ^{13}C NMR: δ = 14.0, 22.6, 25.8, 29.0, 29.2, 31.7, 36.7, 43.6, 55.9, 110.7, 114.4, 120.8, 130.2, 145.2, 146.7, 173.1 ppm.

1.8. 2-Methoxy-4-[(octanoylamino)methyl]phenyl octanoate (**8**)

Colorless solid; m.p. 71-73 °C; R_F = 0.88 (silica gel, *n*-hexane/ethyl acetate, 6:4); ESI-MS (methanol/chloroform, 4:1): *m/z* (%) = 428 ($[M+Na]^+$, 100%); ^1H NMR: δ = 6.94 (*d*, *J* = 8.0 Hz, 1H), 6.87 (*d*, *J* = 1.9 Hz, 1H), 6.81 (*dd*, *J* = 8.0, 1.9 Hz, 1H), 5.95 (*s*, 1H), 4.38 (*d*, *J* = 5.4 Hz, 2H), 3.78 (*s*, 3H), 2.55 (*t*, *J* = 7.5 Hz, 2H), 2.20 (*t*, 2H), 1.79 – 1.69 (*m*, 2H), 1.68 – 1.59 (*m*, 2H), 1.48 – 1.19 (*m*, 16H), 0.92 – 0.83 (*m*, 6H) ppm; ^{13}C NMR: δ = 14.0, 14.0, 22.6, 25.0, 25.8, 28.9, 29.0, 29.0, 29.3, 29.3, 31.7, 31.7, 34.0, 36.7, 43.4, 55.8, 112.1, 120.0, 122.8, 137.2, 139.2, 151.2, 172.0, 173.2 ppm.

1.9. *N*-[(4-Hydroxy-3-methoxy)benzyl]nonanamide (**9**)

Colorless solid; m.p. 48-50 °C; R_F = 0.65 (silica gel, *n*-hexane/ethyl acetate, 4:6); ESI-MS (methanol/chloroform, 4:1): *m/z* (%) = 292 ($[M-H]^-$, 100%); ^1H NMR: δ = 6.85 (*d*, *J* = 8.0 Hz, 1H), 6.80 (*d*, *J* = 1.9 Hz, 1H), 6.75 (*dd*, *J* = 8.0, 1.7 Hz, 1H), 5.84 (*s*, 1H), 4.35 (*d*, *J* = 4.3 Hz, 2H), 3.87 (*s*, 3H), 2.21 (*t*, *J* = 7.6 Hz, 2H), 1.70 – 1.60 (*m*, 2H), 1.36 – 1.20 (*m*, 11H), 0.87 (*t*, *J* = 6.9 Hz, 3H) ppm; ^{13}C NMR: δ = 14.0, 22.6, 25.8, 29.1, 29.3, 29.3, 31.8, 36.7, 43.6, 55.9, 110.7, 114.4, 120.8, 130.2, 145.2, 146.7, 173.2 ppm.

1.10. 2-Methoxy-4-[(nonanoylamino)methyl]phenyl nonanoate (**10**)

Colorless solid; m.p. 74 °C; R_F = 0.8 (silica gel, *n*-hexane/ethyl acetate, 4:6); ESI-MS (methanol/chloroform, 4:1): *m/z* (%) = 432 ($[M-H]^-$, 100%); ^1H NMR: δ = 6.97 (*d*, *J* = 8.0 Hz, 1H), 6.89 (*s*, 1H), 6.84 (*d*, *J* = 8.0 Hz, 1H), 5.76 (*s*, 1H), 4.44 – 4.36 (*m*, 2H), 3.80 (*s*, 3H), 2.56 (*t*, *J* = 7.5 Hz, 2H), 2.22 (*t*, *J* = 8.1 Hz, 2H), 1.81 – 1.71 (*m*, 2H), 1.71 – 1.18 (*m*, 22H), 0.94 – 0.82 (*m*, 6H) ppm; ^{13}C NMR: δ = 14.2, 14.2, 22.8, 22.8, 25.2, 26.0, 29.2, 29.2, 29.4, 29.4, 29.5, 29.5, 32.0, 32.0, 34.2, 37.0, 43.7, 56.1, 112.4, 120.2, 123.1, 139.4, 151.5, 172.1, 173.3 ppm.

1.11. *N*-[(4-Hydroxy-3-methoxy)benzyl]decanamide (**11**)

Colorless solid; m.p. 55-57 °C; R_F = 0.16 (silica gel, *n*-hexane/ethyl acetate, 6:4); ESI-MS (methanol/chloroform, 4:1): *m/z* (%) = 331 ($[M+Na]^+$, 100%); ^{1}H NMR: δ = 6.85 (*d*, *J* = 8.0 Hz, 1H), 6.80 (*d*, 1H), 6.75 (*dd*, *J* = 8.0, 1.4 Hz, 1H), 5.84 (*s*, 1H), 4.34 (*d*, *J* = 4.0 Hz, 2H), 3.87 (*s*, 3H), 2.20 (*t*, *J* = 7.5 Hz, 2H), 1.70 – 1.58 (*m*, 2H), 1.34 – 1.20 (*m*, 12H), 0.87 (*t*, *J* = 6.8 Hz, 3H) ppm; ^{13}C NMR: δ = 14.1, 22.6, 25.8, 29.2, 29.3, 29.3, 29.4, 31.8, 36.9, 43.6, 56.0, 110.8, 114.4, 120.8, 130.3, 145.2, 146.7, 173.1 ppm.

1.12. 2-Methoxy-4-[(decanoylamino)methyl]phenyl decanoate (**12**)

Colorless solid; m.p. 99 °C; R_F = 0.70 (silica gel, *n*-hexane/ethyl acetate, 6:4); ESI-MS (methanol/chloroform, 4:1): *m/z* (%) = 460 ($[M-H]^-$, 100%); ^{1}H NMR: δ = 6.85 (*d*, *J* = 8.0 Hz, 1H), 6.75 (*d*, *J* = 1.9 Hz, 1H), 6.72 (*dd*, *J* = 8.0, 1.9 Hz, 1H), 6.52 (*s*, 1H), 4.42 – 4.25 (*m*, 2H), 3.86 (*s*, 3H), 3.42 (*t*, *J* = 7.4 Hz, 2H), 2.53 (*t*, *J* = 7.2, 5.7 Hz, 2H), 1.87 – 1.75 (*m*, 2H), 1.58 – 1.48 (*m*, 2H), 1.32 – 1.20 (*m*, 24H), 0.87 (*m*, 6H) ppm; ^{13}C NMR: δ = 14.1, 14.1, 22.6, 22.6, 23.3, 27.5, 29.0, 29.1, 29.1, 29.2, 29.2, 29.3, 29.3, 29.3, 29.3, 31.2, 31.8, 31.8, 42.9, 43.5, 55.9, 60.7, 110.4, 114.4, 120.6, 130.0, 145.1, 146.6, 168.5, 210.2 ppm.

1.13. *N*-[(4-Hydroxy-3-methoxy)benzyl]undecanamide (**13**)

Colorless solid; m.p. 60-63 °C; R_F = 0.24 (silica gel, *n*-hexane/ethyl acetate, 6:4); ESI-MS (methanol/chloroform, 4:1): *m/z* (%) = 344 ($[M+Na]^+$, 100%); : ^{1}H NMR: δ = 6.86 (*d*, *J* = 8.0 Hz, 1H), 6.81 (*d*, *J* = 1.9 Hz, 1H), 6.75 (*dd*, *J* = 8.0, 1.8 Hz, 1H), 5.79 (*s*, 1H), 4.35 (*d*, *J* = 4.5 Hz, 2H), 3.87 (*s*, 3H), 2.21 (*t*, *J* = 7.6 Hz, 2H), 1.70 – 1.58 (*m*, 2H), 1.37 – 1.19 (*m*, 14H), 0.87 (*t*, *J* = 6.9 Hz, 3H) ppm; ^{13}C NMR: δ = 14.1, 22.6, 25.8, 29.3, 29.3, 29.3, 29.5, 29.5, 31.9, 36.8, 43.6, 55.9, 110.7, 114.3, 120.8, 130.3, 145.1, 146.7, 173.0 ppm.

1.14. 2-Methoxy-4-[(undecanoylamino)methyl]phenyl undecanoate (**14**)

Colorless solid; m.p. 81-83 °C; R_F = 0.64 (silica gel, *n*-hexane/ethyl acetate, 6:4); ESI-MS (methanol/chloroform, 4:1): *m/z* (%) = 513 ($[M+Na]^+$, 100%); ^{1}H NMR: δ = 6.96 (*d*, *J* = 8.0 Hz, 1H), 6.89 (*d*, *J* = 1.9 Hz, 1H), 6.83 (*d*, *J* = 8.0, 1.8 Hz, 1H), 5.85 (*s*, 1H), 4.40 (*d*, *J* = 4.8 Hz, 2H), 3.80 (*s*, 3H), 2.56 (*t*, *J* = 7.5 Hz, 2H), 2.22 (*t*, *J* = 7.6 Hz, 2H), 1.80 – 1.71 (*m*, 2H), 1.69 – 1.60 (*m*, 2H), 1.47 – 1.37 (*m*, 2H, 24-H), 1.37 – 1.21 (*m*, 26H), 0.92 – 0.84 (*m*, 6H) ppm; ^{13}C NMR: δ = 14.1, 14.1, 22.7, 22.7, 25.0, 25.8, 29.0, 29.3, 29.3, 29.3, 29.3, 29.3, 29.3, 29.3, 29.5, 29.5, 29.5, 31.9, 31.9, 34.0, 36.7, 43.5, 55.9, 112.2, 120.0, 122.9, 137.2, 139.2, 151.3, 172.0, 173.1 ppm.

1.15. *N*-[(4-Hydroxy-3-methoxy)benzyl]dodecanamide (**15**)

Colorless solid; m.p. 76-78 °C; R_F = 0.25 (silica gel, *n*-hexane/ethyl acetate, 6:4); ESI-MS (methanol/chloroform, 4:1): *m/z* (%) = 358 ($[M+Na]^+$, 100%); ^{1}H NMR: δ = 6.87 (*d*, *J* = 8.0 Hz, 1H), 6.81 (*d*, *J* = 1.9 Hz, 1H), 6.77 (*dd*, *J* = 8.1, 1.9 Hz, 1H), 5.73 (*s*, 1H), 4.36 (*d*, *J* = 5.4 Hz, 2H), 3.88 (*s*, 3H), 2.21 (*t*, 2H), 1.69 – 1.62 (*m*, 2H), 1.35 – 1.22 (*m*, 16H), 0.89 (*t*, *J* = 7.0 Hz, 3H) ppm; ^{13}C NMR: δ = 14.1, 22.7, 25.8, 29.3, 29.3, 29.3, 29.3, 29.5, 29.6, 31.9, 36.8, 43.6, 55.9, 76.7, 77.0, 77.3, 110.7, 114.4, 120.8, 130.3, 145.1, 146.7, 173.0 ppm.

1.16. 2-Methoxy-4-[(dodecanoylamino)methyl]phenyl dodecanoate (**16**)

Colorless solid; m.p. 84 °C; R_F = 0.64 (silica gel, *n*-hexane/ethyl acetate, 6:4); ESI-MS (methanol/chloroform, 4:1): *m/z* (%) = 540 ($[M+Na]^+$, 100%); ^{1}H NMR: δ = 6.95 (*d*, 1H), 6.88 (*d*, *J* = 1.9 Hz, 1H), 6.82 (*d*, *J* = 8.0, 1.9 Hz, 1H), 5.87 (*s*, 1H), 4.39 (*d*, 2H), 3.79 (*d*, *J* = 1.5 Hz, 3H), 2.55 (*t*, *J* = 7.5 Hz, 2H), 2.20 (*t*, *J* = 7.6 Hz, 2H), 1.80 – 1.70 (*m*, 2H), 1.69 – 1.59 (*m*, 2H), 1.46 – 1.36 (*m*, 2H), 1.36 – 1.20 (*m*, 30H), 0.87 (*m*, 6H) ppm; ^{13}C NMR: δ = 14.1, 14.1, 22.7, 22.7, 25.0, 25.8, 29.0, 29.3, 29.3, 29.3, 29.3, 29.3, 29.3, 29.5, 29.5, 29.6, 29.6, 31.9, 31.9, 34.0, 36.7, 43.5, 55.9, 112.2, 120.0, 122.9, 137.2, 139.2, 151.3, 172.0, 173.1 ppm.

1.17. *N*-[(4-Hydroxy-3-methoxy)benzyl]tridecanamide (**17**)

Colorless solid; m.p. 70-72 °C; R_F = 0.11 (silica gel, *n*-hexane/ethyl acetate, 7:3); ESI-MS (methanol/chloroform, 4:1): *m/z* (%) = 373 ($[M+Na]^+$, 100%); ^{1}H NMR: δ = 6.85 (*d*, *J* = 8.0 Hz, 1H), 6.80 (*d*, *J* = 1.9 Hz, 1H), 6.75 (*dd*, *J* = 8.0, 1.9 Hz, 1H), 5.80 (*s*, 1H), 4.34 (*d*, *J* = 5.2 Hz, 2H), 3.86 (*s*, 3H), 2.20 (*t*, *J* = 7.6 Hz, 2H), 1.68 – 1.59 (*m*, 2H), 1.35 – 1.21 (*m*, 18H), 0.86 (*t*, 3H) ppm; ^{13}C NMR: δ = 14.1, 22.7, 25.8, 29.3, 29.3, 29.3, 29.3, 29.5, 29.6, 29.6, 29.6, 31.9, 36.8, 43.5, 55.9, 110.7, 114.4, 120.8, 130.3, 145.1, 146.7, 173.0 ppm.

1.18. 2-Methoxy-4-[(tridecanoylamino)methyl]phenyl tridecanoate (**18**)

Colorless solid; m.p. 79 °C; R_F = 0.24 (silica gel, *n*-hexane/ethyl acetate, 7:3); ESI-MS (methanol/chloroform, 4:1): *m/z* (%) = 569 ($[M+Na]^+$, 100%); ^{1}H NMR: δ = 6.95 (*d*, *J* = 8.0 Hz, 1H), 6.88 (*d*, *J* = 1.9 Hz, 1H), 6.83 (*dd*, *J* = 8.0, 1.9 Hz, 1H), 5.79 (*t*, *J* = 5.7 Hz, 1H), 4.40 (*d*, *J* = 5.3 Hz, 2H), 3.80 (*s*, 3H), 2.56 (*t*, *J* = 7.5 Hz, 2H), 2.25 – 2.16 (*m*, 2H), 1.80 – 1.70 (*m*, 2H), 1.70 – 1.59 (*m*, 2H), 1.47 – 1.36 (*m*, 2H), 1.36 – 1.21 (*m*, 34H), 0.88 (*t*, *J* = 6.7 Hz, 6H)

ppm; ^{13}C NMR: δ = 14.1, 14.1, 22.7, 22.7, 25.0, 25.8, 29.0, 29.3, 29.3, 29.3, 29.3, 29.3, 29.3, 29.5, 29.5, 29.6, 29.6, 29.6, 29.6, 29.6, 29.6, 31.9, 31.9, 34.0, 36.8, 43.4, 55.8, 112.2, 120.0, 122.9, 137.2, 139.2, 151.3, 172.0, 173.1 ppm.

1.19. *N*-[(4-Hydroxy-3-methoxy)benzyl]tetradecanamide (**19**)

Colorless solid; m.p. 76 °C; R_F = 0.13 (silica gel, *n*-hexane/ethyl acetate, 7:3); ESI-MS (methanol/chloroform, 4:1): *m/z* (%) = 386 ($[M+Na]^+$, 100%); ^1H NMR: δ = 6.85 (*d*, *J* = 8.0 Hz, 1H), 6.80 (*d*, *J* = 1.9 Hz, 1H), 6.75 (*dd*, *J* = 8.0, 1.9 Hz, 1H), 5.83 (*s*, 1H), 4.34 (*d*, *J* = 4.7 Hz, 2H), 3.87 (*s*, 3H), 2.20 (*t*, *J* = 7.6 Hz, 2H), 1.70 – 1.59 (*m*, 2H), 1.35 – 1.22 (*m*, 20H), 0.90 – 0.85 (*m*, 3H) ppm; ^{13}C NMR: δ = 14.1, 22.7, 25.8, 29.3, 29.3, 29.3, 29.3, 29.5, 29.6, 29.6, 29.6, 29.6, 31.9, 36.8, 43.6, 55.9, 110.7, 114.4, 120.8, 130.3, 145.1, 146.7, 173.0 ppm.

1.20. 2-Methoxy-4-[(tetradecanoylamino)methyl]phenyl tetradecanoate (**20**)

Colorless solid; m.p. 85-87 °C; R_F = 0.28 (silica gel, *n*-hexane/ethyl acetate, 7:3); ESI-MS (methanol/chloroform, 4:1): *m/z* (%) = 596 ($[M+Na]^+$, 100%); ^1H NMR: δ = 6.95 (*d*, *J* = 8.0 Hz, 1H), 6.91 – 6.86 (*m*, 1H), 6.82 (*dd*, *J* = 8.0, 1.5 Hz, 1H), 5.85 (*s*, 1H), 4.40 (*d*, *J* = 4.3 Hz, 2H), 3.80 (*s*, 3H), 2.56 (*t*, *J* = 7.5 Hz, 2H), 2.21 (*t*, *J* = 7.5 Hz, 2H), 1.82 – 1.70 (*m*, 2H), 1.69 – 1.59 (*m*, 2H), 1.47 – 1.37 (*m*, 2H), 1.37 – 1.18 (*m*, 38H), 0.88 (*t*, *J* = 6.7 Hz, 7H) ppm; ^{13}C NMR: δ = 14.1, 14.1, 22.7, 22.7, 25.0, 25.8, 29.0, 29.3, 29.3, 29.3, 29.3, 29.3, 29.3, 29.5, 29.5, 29.6, 29.6, 29.6, 29.6, 29.6, 29.6, 29.7, 31.9, 31.9, 34.0, 36.8, 43.5, 55.9, 112.2, 120.0, 122.9, 137.2, 139.2, 151.3, 171.9, 173.0 ppm.

1.21. *N*-[(4-Hydroxy-3-methoxy)benzyl]pentadecanamide (**21**)

Colorless solid; m.p. 79 °C; R_F = 0.11 (silica gel, *n*-hexane/ethyl acetate, 7:3); ESI-MS (methanol/chloroform, 4:1): *m/z* (%) = 400 ($[M+Na]^+$, 100%); ^1H NMR: δ = 6.85 (*d*, *J* = 8.0 Hz, 1H), 6.80 (*d*, *J* = 1.9 Hz, 1H), 6.75 (*dd*, *J* = 8.0, 1.8 Hz, 1H), 5.82 (*s*, 1H), 4.35 (*d*, *J* = 4.5 Hz, 2H), 3.87 (*s*, 3H), 2.21 (*t*, *J* = 7.6 Hz, 2H), 1.72 – 1.56 (*m*, 2H), 1.38 – 1.17 (*m*, 22H), 0.88 (*t*, *J* = 6.8 Hz, 3H) ppm; ^{13}C NMR: δ = 14.1, 22.7, 25.8, 29.3, 29.3, 29.5, 29.6, 29.6, 29.6, 29.6, 29.7, 29.7, 31.9, 36.8, 43.6, 55.9, 110.7, 114.4, 120.8, 130.3, 145.2, 146.7, 173.0 ppm.

1.22. 2-Methoxy-4-[(pentadecanoylamino)methyl]phenyl pentadecanoate (**22**)

Colorless solid; m.p. 79-80 °C; R_F = 0.75 (silica gel, *n*-hexane/ethyl acetate, 6:4); ESI-MS (methanol/chloroform, 4:1): *m/z* (%) = 625 ($[M+Na]^+$, 100%); ^1H NMR: δ = 6.96 (*d*, *J* = 8.0

Hz, 1H), 6.89 (*d*, *J* = 1.9 Hz, 1H), 6.83 (*dd*, *J* = 8.1, 1.9 Hz, 1H), 5.74 (*t*, *J* = 5.7 Hz, 1H), 4.41 (*d*, *J* = 5.4 Hz, 2H), 3.80 (*s*, 3H), 2.56 (*t*, *J* = 7.5 Hz, 2H), 2.40 – 2.24 (*m*, 2H), 2.26 – 2.15 (*m*, 2H, 26-H), 1.81 – 1.70 (*m*, 2H), 1.69 – 1.58 (*m*, 2H), 1.48 – 1.36 (*m*, 2H), 1.36 – 1.17 (*m*, 36H), 1.00 – 0.76 (*m*, 10H) ppm; ^{13}C NMR: δ = 14.1, 14.1, 22.7, 22.7, 25.0, 25.8, 29.3, 29.3, 29.3, 29.3, 29.7, 29.7, 29.7, 29.7, 29.7, 29.7, 29.6, 29.6, 29.6, 29.6, 29.6, 29.6, 29.6, 31.9, 31.9, 34.0, 36.8, 43.5, 55.9, 112.2, 120.0, 122.9, 137.1, 139.2, 151.3, 172.0, 173.1 ppm.

1.23. *N*-[(4-Hydroxy-3-methoxy)benzyl]hexadecanamide (**23**)

Colorless solid; m.p. 80 °C; R_F = 0.12 (silica gel, *n*-hexane/ethyl acetate, 7:3); ESI-MS (methanol/chloroform, 4:1): *m/z* (%) = 390 ([M-H]$^-$, 100%); ^{1}H NMR: δ = 6.84 (*d*, *J* = 8.0 Hz, 1H), 6.75 (*d*, *J* = 1.9 Hz, 1H), 6.72 (*dd*, *J* = 8.0, 2.0 Hz, 1H), 5.62 (*s*, 1H), 4.42 – 4.24 (*m*, 2H), 3.86 (*s*, 3H), 2.55 – 2.48 (*m*, 2H), 1.80 (*t*, *J* = 7.6 Hz, 2H), 1.42 – 1.12 (*m*, 24H), 0.92 – 0.82 (*m*, 3H) ppm; ^{13}C NMR: δ = 14.1, 22.7, 23.3, 27.5, 29.0, 29.3, 29.3, 29.4, 29.4, 29.5, 29.6, 29.6, 29.7, 31.2, 31.9, 42.9, 43.5, 55.9, 60.8, 110.4, 114.4, 120.6, 130.0, 145.1, 146.6, 168.5 ppm.

1.24. 2-Methoxy-4-[(hexadecanoylamino)methyl]phenyl hexadecanoate (**24**)

Colorless solid; m.p. 91-92 °C; R_F = 0.45 (silica gel, *n*-hexane/ethyl acetate, 7:3); ESI-MS (methanol/chloroform, 4:1): *m/z* (%) = 665 ([M+Na]$^+$, 100%); ^{1}H NMR: δ = 6.95 (*d*, *J* = 8.0 Hz, 1H), 6.88 (*d*, *J* = 1.9 Hz, 1H), 6.82 (*dd*, *J* = 8.0, 1.9 Hz, 1H), 5.75 (*t*, *J* = 5.7 Hz, 1H), 4.40 (*d*, *J* = 5.7 Hz, 2H), 3.79 (*s*, 3H), 2.56 (*t*, *J* = 7.5 Hz, 2H), 2.23 – 2.15 (*m*, 2H), 1.81 – 1.70 (*m*, 2H), 1.69 – 1.58 (*m*, 2H), 1.49 – 1.18 (*m*, 48H), 0.92 – 0.81 (*m*, 6H, 24-H) ppm; ^{13}C NMR: δ = 14.1, 22.7, 25.0, 25.8, 29.0–29.7, 31.9, 34.0, 36.8, 43.4, 55.8, 112.2, 120.0, 122.9, 137.3, 139.2, 151.3, 171.9, 172.9 ppm.

1.25. *N*-[(4-Hydroxy-3-methoxy)benzyl]heptadecanamide (**25**)

Colorless solid; m.p. 84 °C; R_F = 0.15 (silica gel, *n*-hexane/ethyl acetate, 7:3); ESI-MS (methanol/chloroform, 4:1): *m/z* (%) = 404 ([M-H]$^-$, 100%); ^{1}H NMR: δ = 6.85 (*d*, *J* = 8.0 Hz, 1H), 6.79 (*d*, *J* = 2.0 Hz, 1H), 6.74 (*dd*, *J* = 8.0, 2.0 Hz, 1H), 5.73 – 5.66 (*m*, 1H), 4.34 (*d*, *J* = 5.5 Hz, 2H), 3.86 (*s*, 3H), 2.18 (*t*, *J* = 7.4 Hz, 2H), 1.67 – 1.56 (*m*, 2H), 1.24 (*d*, *J* = 3.2 Hz, 26H), 0.91 – 0.80 (*m*, 3H) ppm; ^{13}C NMR: δ = 14.1, 22.7, 25.8, 29.3–29.7, 31.9, 36.8, 43.5, 55.9, 110.7, 114.3, 120.8, 130.4, 145.1, 146.7, 172.9ppm.

1.26. 2-Methoxy-4-[(heptadecanoylamino)methyl]phenyl heptadecanoate (**26**)

Colorless solid; m.p. 94 °C; R_F = 0.45 (silica gel, *n*-hexane/ethyl acetate, 7:3); ESI-MS (methanol/chloroform, 4:1): *m/z* (%) = 680 ($[M+Na]^+$, 100%); ^{1}H NMR: δ = 6.84 (*d*, *J* = 8.0 Hz, 1H), 6.75 (*d*, *J* = 1.9 Hz, 1H), 6.72 (*dd*, *J* = 8.0, 2.0 Hz, 1H), 5.69 – 5.58 (*m*, 1H), 4.38 – 4.27 (*m*, 2H), 3.86 (*s*, 3H), 2.65 – 2.43 (*m*, 2H), 1.88 – 1.65 (*m*, 2H), 1.43 – 1.14 (*m*, 56H), 0.95 – 0.81 (*m*, 6H) ppm; ^{13}C NMR: δ = 14.1, 22.7, 23.3, 27.5–29.7, 31.2, 31.9, 43.5, 55.9, 110.4, 114.4, 120.6, 130.0, 145.1, 146.6, 168.5, 173.7 ppm.

1.27. *N*-[(4-Hydroxy-3-methoxy)benzyl]octadecanamide (**27**)

Colorless solid; m.p. 87-89 °C; R_F = 0.10 (silica gel, *n*-hexane/ethyl acetate, 7:3); ESI-MS (methanol/chloroform, 4:1): *m/z* (%) = 418 ($[M-H]^-$, 100%); ^{1}H NMR: δ = 6.85 (*d*, *J* = 8.0 Hz, 1H), 6.79 (*d*, *J* = 1.9 Hz, 1H), 6.74 (*dd*, *J* = 8.0, 2.0 Hz, 1H), 5.69 (*t*, *J* = 5.9 Hz, 1H), 4.34 (*d*, *J* = 5.6 Hz, 2H), 3.86 (*s*, 3H), 2.24 – 2.11 (*m*, 2H), 1.66 – 1.58 (*m*, 2H), 1.44 – 1.11 (*m*, 28H), 0.87 (*t*, *J* = 6.7 Hz, 3H) ppm; ^{13}C NMR: δ = 14.1, 22.7, 25.8, 29.7–29.0, 29.7, 31.9, 43.5, 55.9, 110.7, 114.4, 120.8, 130.4, 145.1, 146.7, 172.9 ppm.

1.28. 2-Methoxy-4-[(octadecanoylamino)methyl]phenyl octadecanoate (**28**)

Colorless solid; 94 °C; R_F = 0.49 (silica gel, *n*-hexane/ethyl acetate, 7:3); ESI-MS (methanol/chloroform, 4:1): *m/z* (%) = 709 ($[M+Na]^+$, 100%); ^{1}H NMR: δ = 6.95 (*d*, *J* = 8.0 Hz, 1H), 6.88 (*d*, *J* = 1.9 Hz, 1H), 6.82 (*dd*, *J* = 8.1, 1.8 Hz, 1H), 5.73 – 5.65 (*m*, 1H), 4.40 (*d*, *J* = 5.3 Hz, 2H), 3.80 (*s*, 3H), 2.56 (*t*, *J* = 7.5 Hz, 2H), 2.20 (*t*, *J* = 7.6 Hz, 2H), 1.78 – 1.70 (*m*, 2H), 1.71 – 1.56 (*m*, 2H), 1.36 – 1.16 (*m*, 56H), 0.93 – 0.84 (*m*, 6H) ppm; ^{13}C NMR: δ = 14.1, 22.7, 25.0, 25.8, 29.7–29.0, 31.9, 34.0, 36.8, 43.5, 55.9, 112.2, 120.0, 122.9, 137.2, 139.2, 151.3, 171.9, 173.0 ppm.

1.29. *N*-[(4-Hydroxy-3-methoxy)benzyl]nonadecanamide (**29**)

Colorless solid; m.p. 92 °C; R_F = 0.18 (silica gel, *n*-hexane/ethyl acetate, 7:3); ESI-MS (methanol/chloroform, 4:1): *m/z* (%) = 432 ($[M-H]^-$, 100%); ^{1}H NMR: δ = 6.86 (*d*, *J* = 8.0 Hz, 1H), 6.80 (*d*, *J* = 1.9 Hz, 1H), 6.76 (*dd*, *J* = 8.1, 1.9 Hz, 1H), 5.76 – 5.64 (*m*, 1H), 4.35 (*d*, *J* = 5.4 Hz, 2H), 3.87 (*s*, 3H), 2.22 – 2.15 (*m*, 2H), 1.69 – 1.58 (*m*, 2H), 1.25 (*d*, *J* = 4.2 Hz, 30H), 0.88 (*t*, *J* = 6.9 Hz, 3H) ppm; ^{13}C NMR: δ = 14.1, 22.7, 25.8, 29.7–29.3, 31.9, 36.8, 43.5, 55.9, 110.7, 114.3, 120.8, 130.4, 145.1, 146.7, 172.9 ppm.

1.30. 2-Methoxy-4-[(nonadecanoylamino)methyl]phenyl nonadecanoate (**30**)

Colorless solid; m.p. 97 °C; R_F = 0.55 (silica gel, *n*-hexane/ethyl acetate, 7:3); ESI-MS (methanol/chloroform, 4:1): *m/z* (%) = 737 ($[M+Na]^+$, 100%); ^{1}H NMR: δ = 6.96 (*d*, *J* = 7.9 Hz, 1H), 6.89 (*d*, *J* = 1.9 Hz, 1H), 6.83 (*dd*, *J* = 8.0, 1.7 Hz, 1H), 5.75 – 5.61 (*m*, 1H), 4.41 (*d*, *J* = 5.0 Hz, 2H), 3.80 (*s*, 3H), 2.56 (*t*, *J* = 7.5 Hz, 2H), 2.20 (*t*, *J* = 7.6 Hz, 2H), 1.78 – 1.72 (*m*, 2H), 1.68 – 1.61 (*m*, 2H), 1.43 – 1.18 (*m*, 60H), 0.91 – 0.83 (*m*, 6H, 27-H) ppm; ^{13}C NMR: δ = 14.1, 22.7, 25.0, 25.8, 29.7–29.1, 31.9, 34.0, 36.8, 43.5, 55.9, 112.2, 120.0, 122.9, 137.2, 139.2, 151.3, 171.9, 172.9 ppm.

1.31. *N*-[(4-Hydroxy-3-methoxy)benzyl]icosanamide (**31**)

Colorless solid; m.p. 94 °C; R_F = 0.38 (silica gel, *n*-hexane/ethyl acetate, 6:4); ESI-MS (methanol/chloroform, 4:1): *m/z* (%) = 446 ($[M-H]^-$, 100%); ^{1}H NMR: δ = 6.96 (*d*, *J* = 8.0 Hz, 1H), 6.89 (*d*, *J* = 1.9 Hz, 1H), 6.81 (*dd*, *J* = 5.4, 1.9 Hz, 1H), 5.84 – 5.63 (*m*, 1H), 4.41 (*d*, *J* = 5.2 Hz, 2H), 3.80 (*s*, 3H), 2.56 (*t*, *J* = 7.5 Hz, 2H), 2.20 (*td*, *J* = 7.6, 4.5 Hz, 2H), 1.75 (*p*, *J* = 7.5 Hz, 2H), 1.69 – 1.58 (*m*, 2H), 1.44 – 1.36 (*m*, 2H), 1.35 – 1.21 (*m*, 26H), 0.90 – 0.84 (*m*, 3H) ppm; ^{13}C NMR: δ = 14.1, 22.7, 25.0, 25.8, 29.7–29.1, 31.9, 34.0, 43.5, 55.9, 76.7, 77.0, 77.3, 110.7, 112.2, 114.3, 120.0, 120.8, 122.9, 137.2 ppm.

1.32. 2-Methoxy-4-[(icosanoylamino)methyl]phenyl icosanoate (**32**)

Colorless solid; m.p. 80 °C; R_F = 0.75 (silica gel, *n*-hexane/ethyl acetate, 6:4); ESI-MS (methanol/chloroform, 4:1): *m/z* (%) = 764 ($[M+Na]^+$, 100%); ^{1}H NMR: δ = 6.96 (*d*, *J* = 7.8 Hz, 1H), 6.89 (*s*, 1H), 6.83 (*d*, *J* = 7.9 Hz, 1H), 5.81 – 5.76 (*m*, 1H), 4.43 – 4.39 (*m*, 2H), 3.80 (*s*, 3H), 2.56 (*t*, *J* = 7.5 Hz, 2H), 2.29 – 2.12 (*m*, 2H), 1.85 – 1.69 (*m*, 2H), 1.69 – 1.60 (*m*, 2H), 1.50 – 1.11 (*m*, 64H), 0.88 (*t*, *J* = 6.7 Hz, 6H) ppm; ^{13}C NMR: δ = 14.3, 22.8, 25.2, 26.0, 29.8–29.2, 32.1, 34.2, 37.0, 43.7, 56.1, 76.8, 77.2, 77.5, 112.4, 120.2, 123.0, 137.3, 139.4, 151.4, 172.1, 173.3 ppm.

1.33. *N,N*-bis(4-hydroxy-3-methoxybenzyl)nonanamide (**33**)

Colorless oil; R_F = 0.55 (*n*-hexane/ethyl acetate, 5:5); ESI-MS (methanol/chloroform, 4:1): *m/z* (%) = 430 ($[M+H]^+$, 100%); ^{1}H NMR: δ = 6.95 – 6.50 (*m*, 6H), 4.44 (*d*, *J* = 54.6 Hz, 4H), 3.83 (*s*, 6H), 2.42 (*t*, *J* = 7.5 Hz, 2H), 1.73 – 1.68 (*m*, 2H), 1.36 – 1.21 (*m*, 10H), 0.87 (*t*, *J* = 6.8 Hz,

3H) ppm; ^{13}C NMR: δ = 14.2, 22.8, 25.8, 29.3, 29.5, 29.6, 32.0, 33.5, 49.9, 56.1, 109.0, 111.3, 114.8, 119.6, 121.7, 128.5, 129.7, 145.2, 146.9, 174.0 ppm.

1.34. 4-{[4-Hydroxy-3-methoxybenzyl)(nonanoyl)amino]methyl}-2-methoxyphenyl nonanoate (**34**)

Colorless oil; R_F = 0.65 (*n*-hexane/ethyl acetate, 5:5); ESI-MS (methanol/chloroform, 4:1): *m/z* (%) = 592 ($[M+Na]^+$, 100%); ^{1}H NMR: δ = 7.02 – 6.53 (*m*, 6H), 5.80 (*d*, *J* = 22.9 Hz, 1H, OH), 4.53 (*d*, *J* = 10.3 Hz, 2H), 4.39 (*d*, *J* = 13.7 Hz, 2H), 3.82 (*d*, *J* = 1.4 Hz, 3H), 3.75 (*d*, *J* = 1.4 Hz, 3H), 2.63 – 2.50 (*m*, 2H), 2.50 – 2.32 (*m*, 2H), 1.79 – 1.65 (*m*, 4H), 1.46 – 1.17 (*m*, 20H), 0.92 – 0.81 (*m*, 6H) ppm; ^{13}C NMR: δ = 14.0, 14.0, 22.6, 22.6, 25.0, 25.6, 29.0–29.5, 31.8, 31.8, 33.3, 34.0, 47.9, 50.0, 55.8, 55.9, 110.2, 112.6, 114.7, 121.5, 122.6, 123.2, 129.4, 136.4, 139.2, 145.2, 147.1, 151.3, 171.9, 173.9 ppm.

1.35. (Nonanoylimino) bis(methylene-2-methoxy-4,1-phenylene) dinonanoate (**35**)

Colorless oil; R_F = 0.8 (*n*-hexane/ethyl acetate, 5:5); ESI-MS (methanol/chloroform, 4:1): *m/z* (%) = 733 ($[M+Na]^+$, 100%); ^{1}H NMR: δ = 7.10 – 6.62 (*m*, 6H,), 4.57 (*s*, 2H), 4.43 (*s*, 2H), 3.77 (*s*, 6H), 2.74 – 2.26 (*m*, 6H), 1.85 – 1.54 (*m*, 6H), 1.44 – 1.17 (*m*, 30H), 0.95 – 0.76 (*m*, 9H) ppm; ^{13}C NMR: δ = 14.0, 14.0, 14.0, 22.6–34.0, 48.0, 49.8, 55.8, 55.9, 110.2, 112.6, 118.3, 120.5, 122.7, 123.2, 135.2, 136.2, 139.2, 139.2, 151.3, 151.6, 171.9, 174.0, 179.3 ppm.

1.36. *N*-[(4-Hydroxy-3-methoxyphenyl)methyl]-2-methyloctanamide (**36**)

Colorless solid; m.p. 78-80 °C; R_F = 0.28 (silica gel, *n*-hexane/ethyl acetate, 4:6); ESI-MS (methanol/chloroform, 4:1): *m/z* (%) = 292 ($[M-H]^-$, 100%); ^{1}H NMR: δ = 6.83 (*d*, *J* = 8.0 Hz, 1H), 6.78 (*d*, *J* = 1.9 Hz, 1H), 6.72 (*dd*, *J* = 8.0, 1.9 Hz, 1H), 5.98 (*t*, *J* = 5.5 Hz, 1H), 5.78 – 4.79 (*m*, 1H, OH), 4.40 – 4.26 (*m*, 2H), 3.83 (*s*, 3H), 2.20 (*m*, 1H), 1.70 – 1.59 (*m*, 1H), 1.42 – 1.32 (*m*, 1H), 1.25 (*m*, 8H), 1.13 (*d*, *J* = 6.8 Hz, 3H), 0.85 (*t*, *J* = 7.0 Hz, 3H) ppm; ^{13}C NMR: δ = 14.1, 18.0, 22.7, 27.6, 29.4, 31.8, 34.5, 41.7, 43.5, 56.0, 110.7, 114.5, 120.7, 130.5, 145.2, 146.9, 176.8 ppm.

1.37. 4-{[(2-Methyloctanoyl)amino]methyl}-2-(methyloxy)phenyl 2-methyl octanoate (**37**)

Colorless solid; m.p. 80-82 °C; R_F = 0.55 (silica gel, *n*-hexane/ethyl acetate, 4:6); ESI-MS (methanol/chloroform, 4:1): *m/z* (%) = 432 ($[M-H]^-$, 100%); ^{1}H NMR: δ = 6.94 (*d*, *J* = 8.0 Hz, 1H), 6.88 (*d*, *J* = 1.9 Hz, 1H), 6.82 (*dd*, *J* = 8.0, 1.9 Hz, 1H), 6.10 – 5.99 (*m*, 1H), 4.47 – 4.38 (*m*, 2H), 3.78 (*s*, 3H), 2.78 – 2.62 (*m*, 1H), 2.33 – 2.18 (*m*, 1H), 1.87 – 1.75 (*m*, 1H), 1.73 – 1.63 (*m*, 1H), 1.59 – 1.48 (*m*, 1H), 1.46 – 1.37 (*m*, 2H), 1.29 (*d*, *J* = 7.0 Hz, 3H), 1.37 – 1.21 (*m*, 15H), 1.17 (*d*, *J* = 6.9 Hz, 3H), 0.91 – 0.84 (*m*, 6H) ppm; ^{13}C NMR: δ = 14.2, 14.2, 17.3, 18.0, 22.7, 22.8, 27.2, 27.6, 29.4, 29.4, 31.9, 31.9, 34.0, 34.5, 39.6, 39.7, 41.7, 43.6, 55.9, 76.8, 77.2, 77.4, 77.5, 112.2, 120.1, 123.0, 137.2, 139.5, 151.5, 175.1, 177.1 ppm.

^{1}H and ^{13}C NMR spectra of compounds 1-37

All NMR spectra were recorded in $CDCl_3$ (400 MHz for ^{1}H and 101 MHz for ^{13}C); for ^{13}C NMR APT spectra: thereby, C_q and CH_2 up, CH + CH_3 down.

Compound **1**

^{1}H NMR Spectrum ($CDCl_3$, 400 MHz)

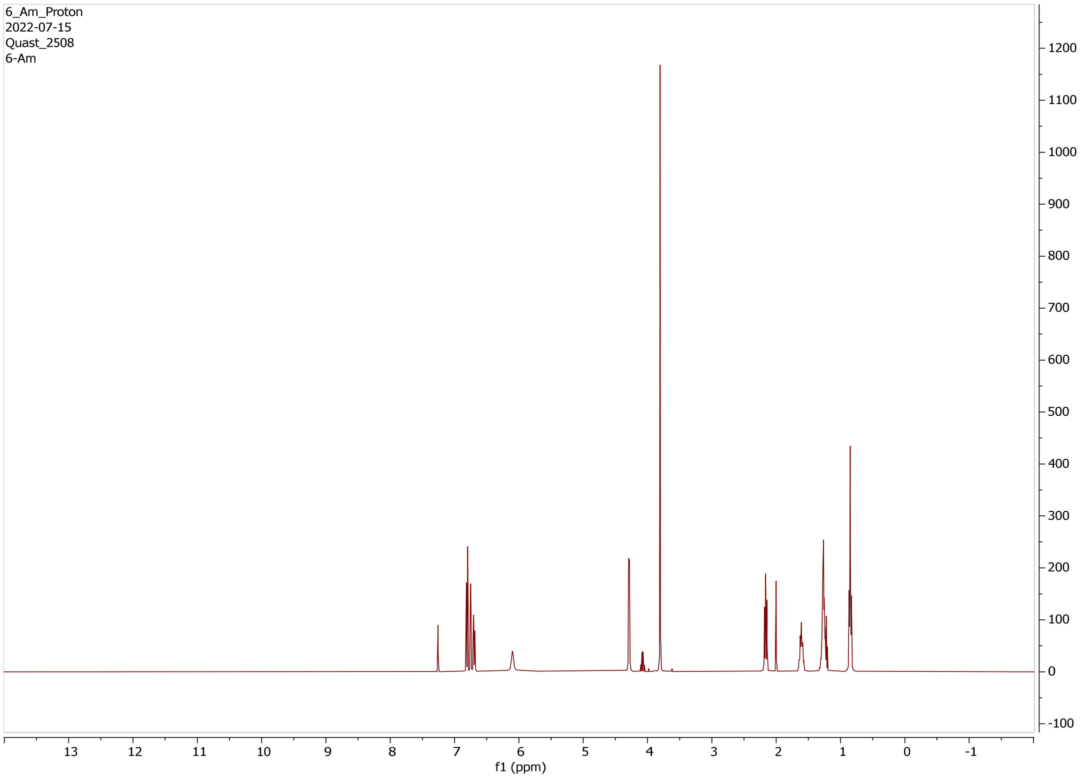

^{13}C-APT NMR Spectrum ($CDCl_3$, 101 MHz)

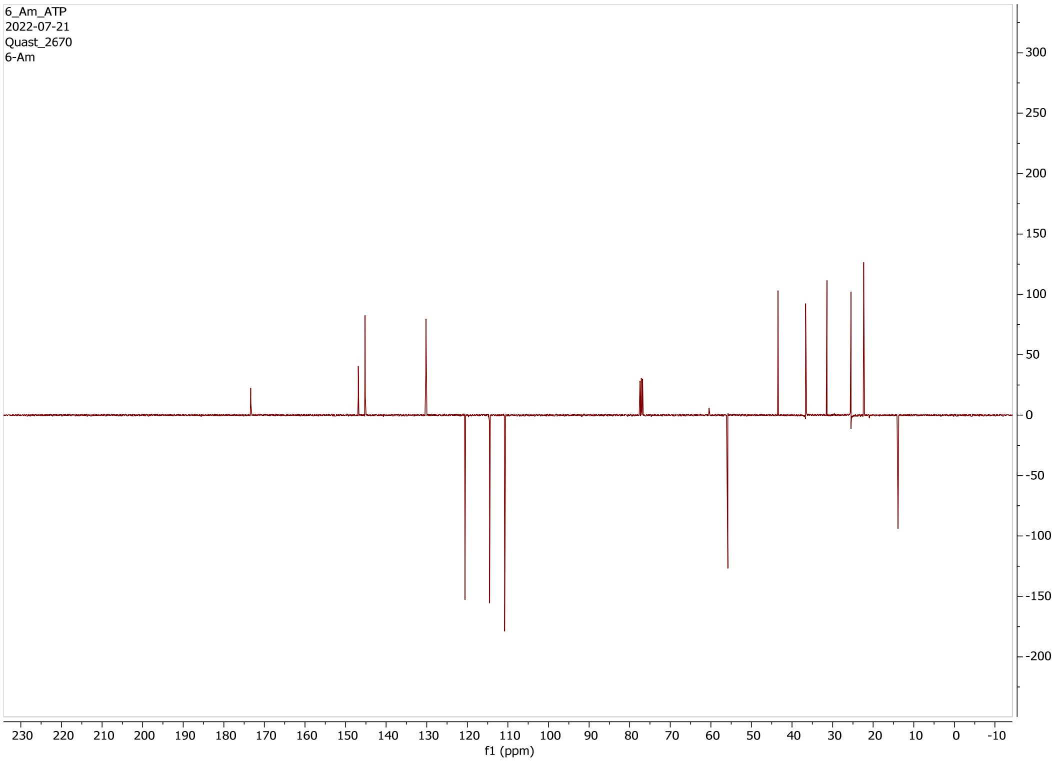

Compound **2**

^{1}H NMR Spectrum ($CDCl_3$, 400 MHz)

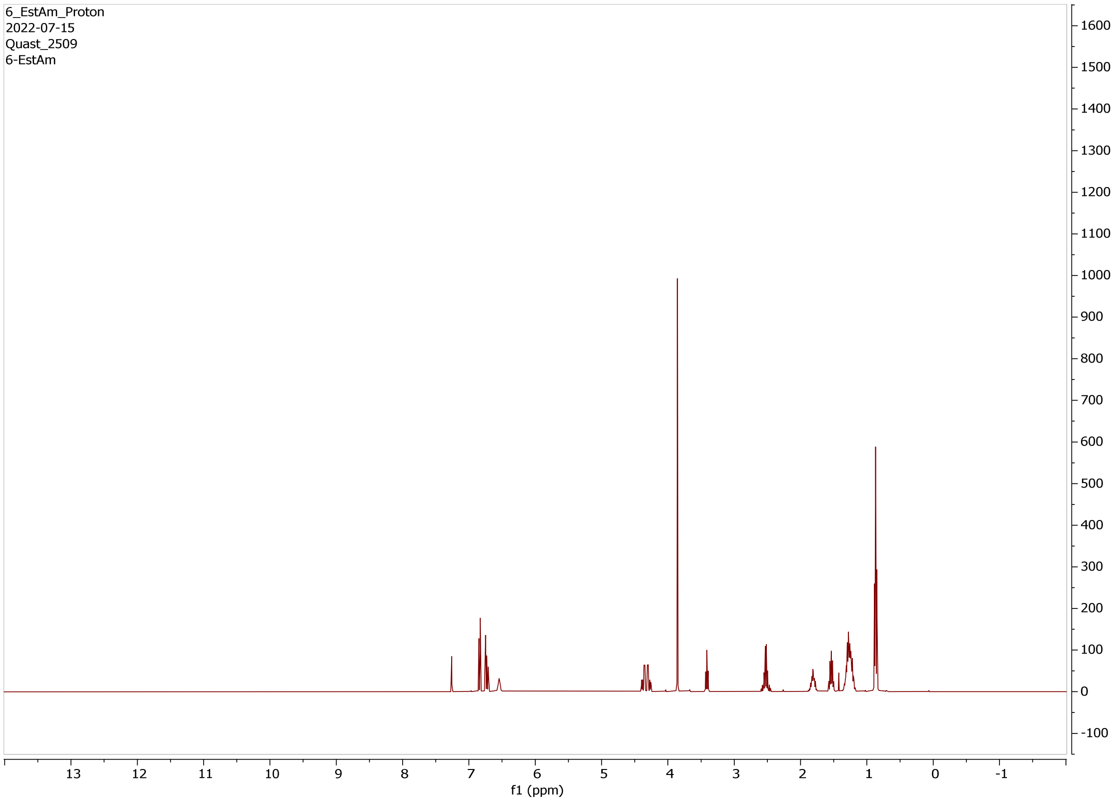

^{13}C-APT NMR Spectrum ($CDCl_3$, 101 MHz)

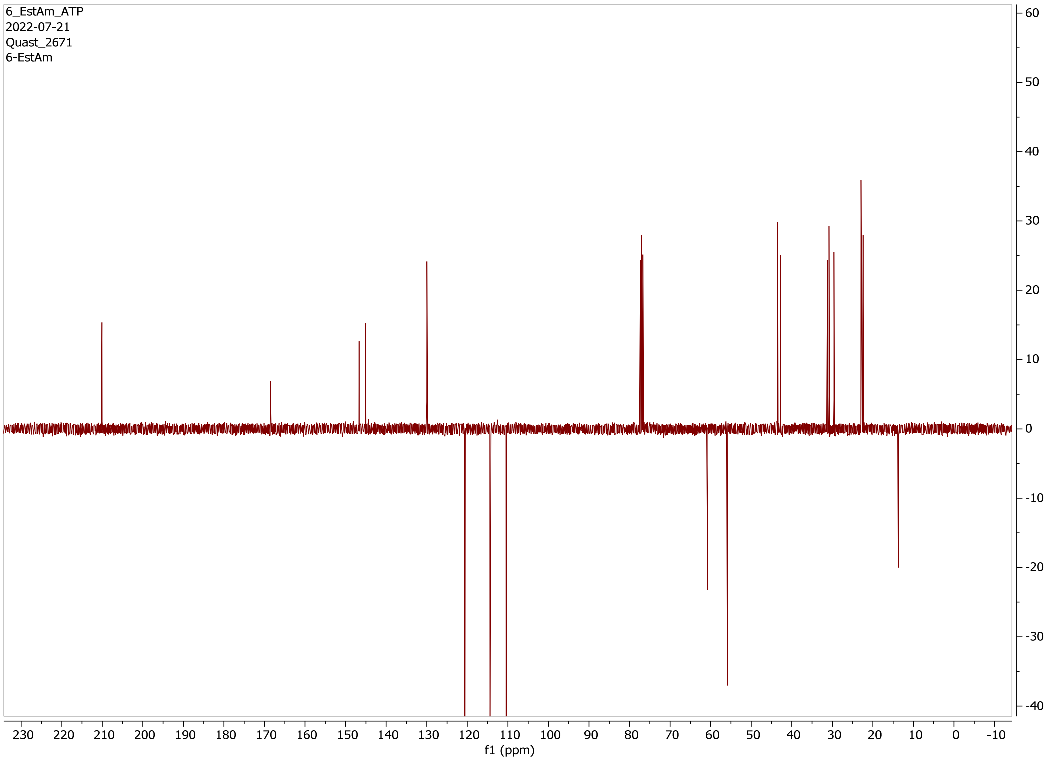

Compound **3**

^{1}H NMR Spectrum ($CDCl_3$, 400 MHz)

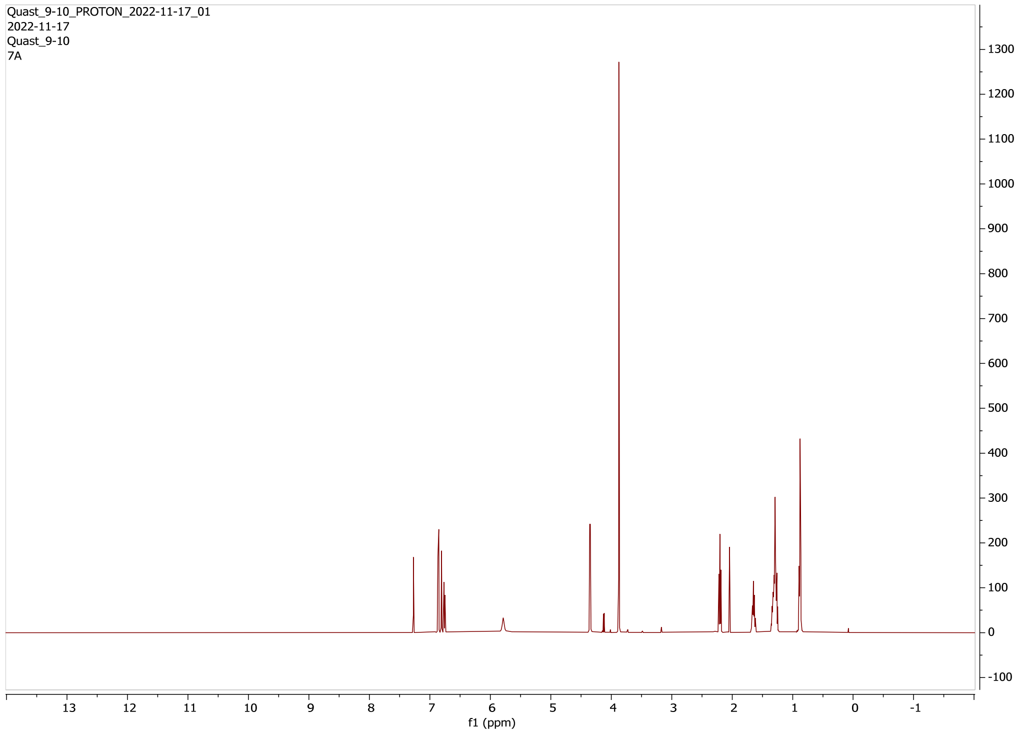

^{13}C-APT NMR Spectrum ($CDCl_3$, 101 MHz)

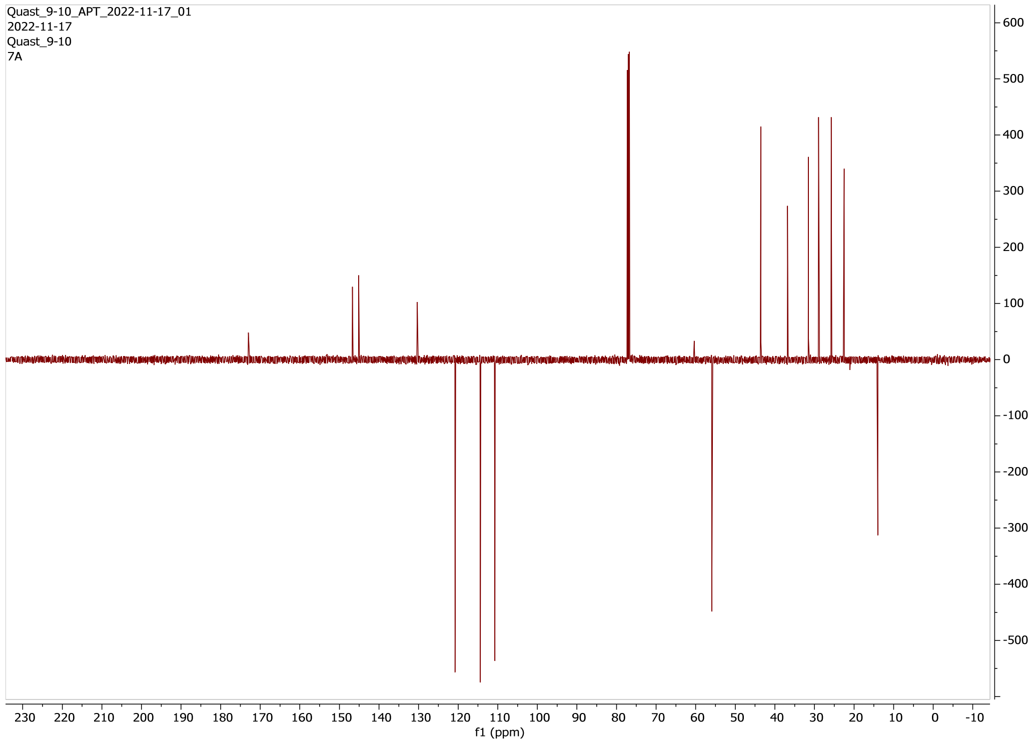

Compound **4**

^{1}H NMR Spectrum ($CDCl_3$, 400 MHz)

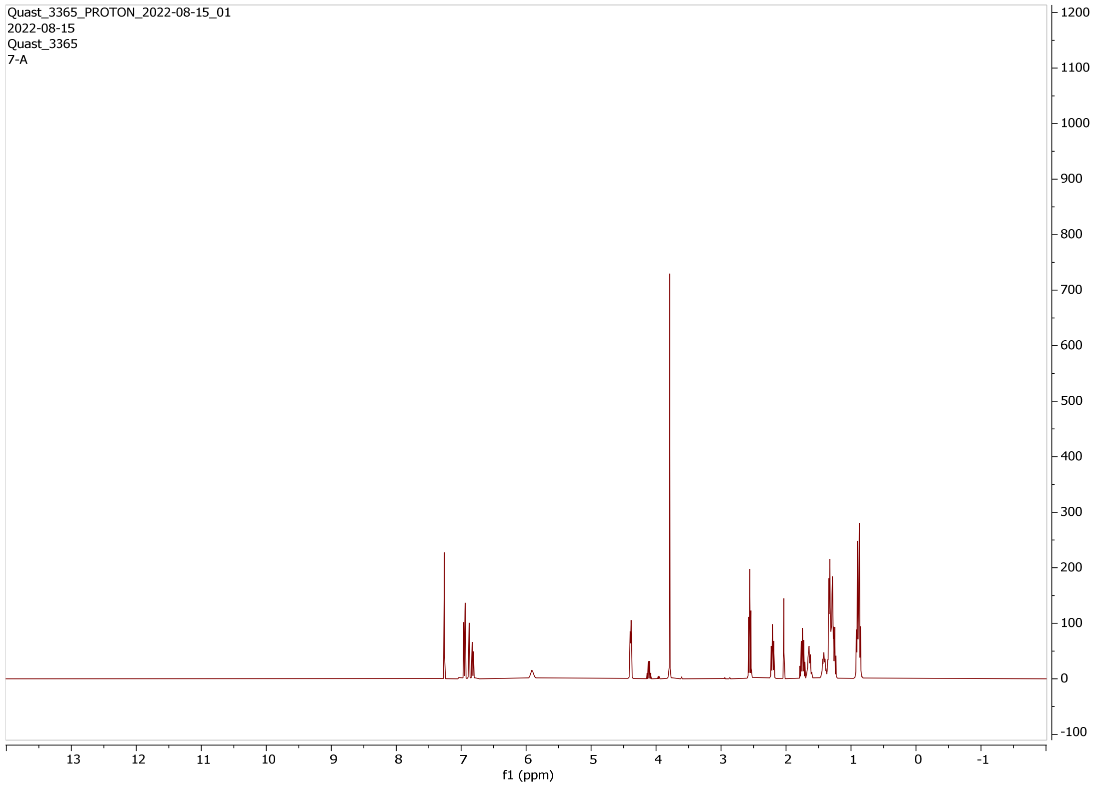

^{13}C-APT NMR Spectrum ($CDCl_3$, 101 MHz)

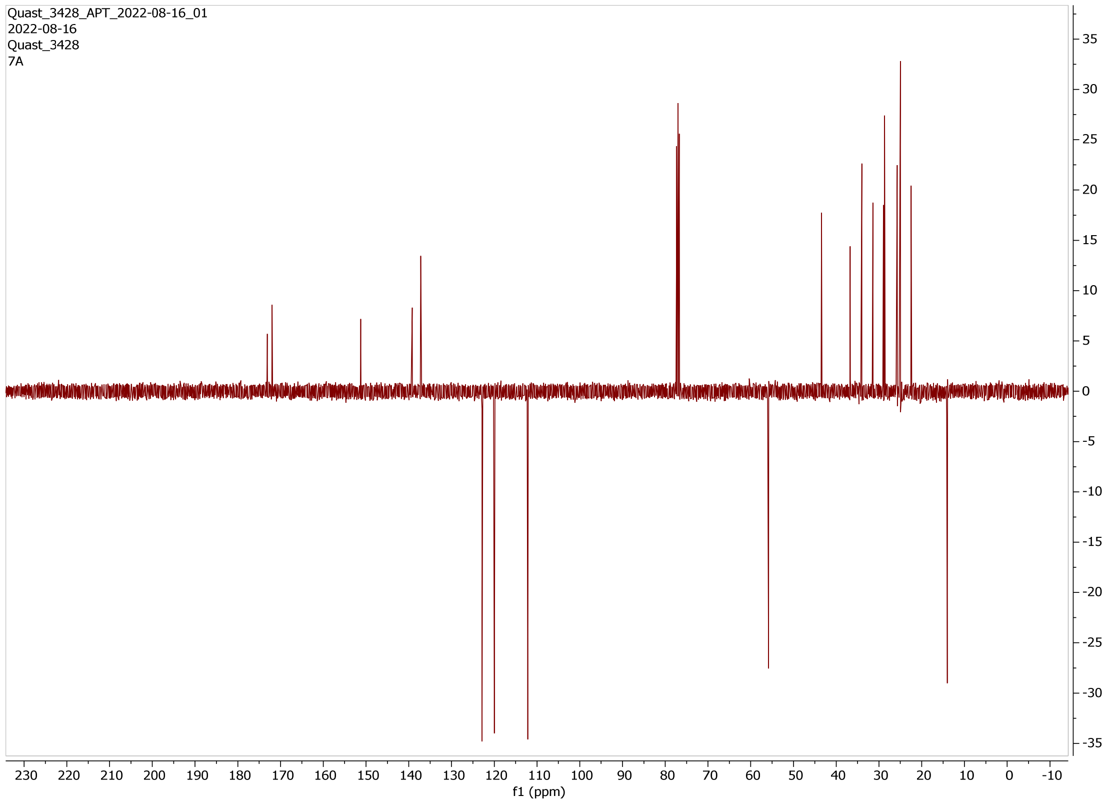

Compound **5**

^{1}H NMR Spectrum ($CDCl_3$, 400 MHz)

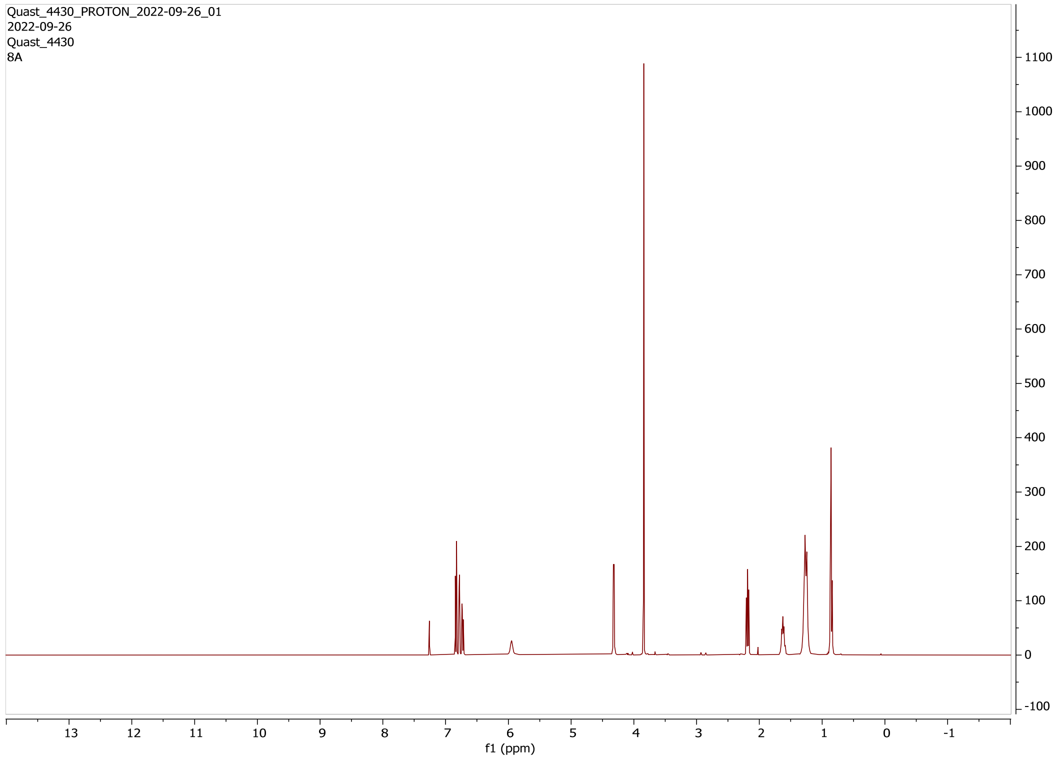

^{13}C-APT NMR Spectrum ($CDCl_3$, 101 MHz)

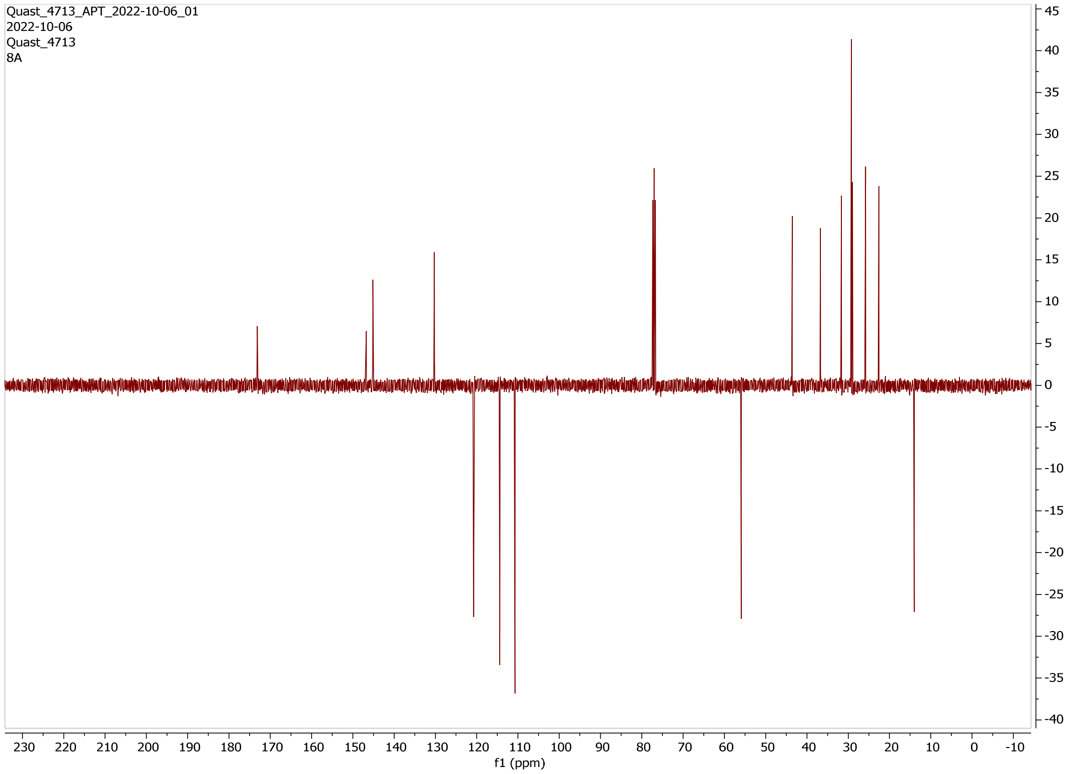

Compound **6**

^{1}H NMR Spectrum ($CDCl_3$, 400 MHz)

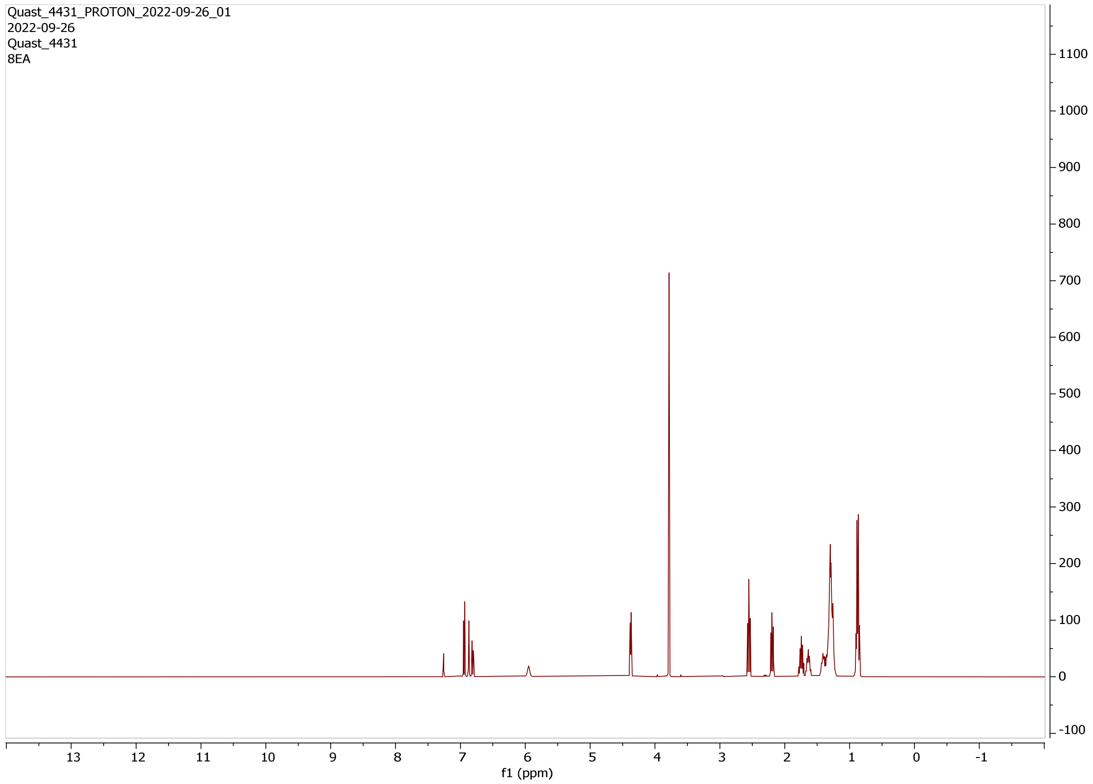

^{13}C-APT NMR Spectrum ($CDCl_3$, 101 MHz)

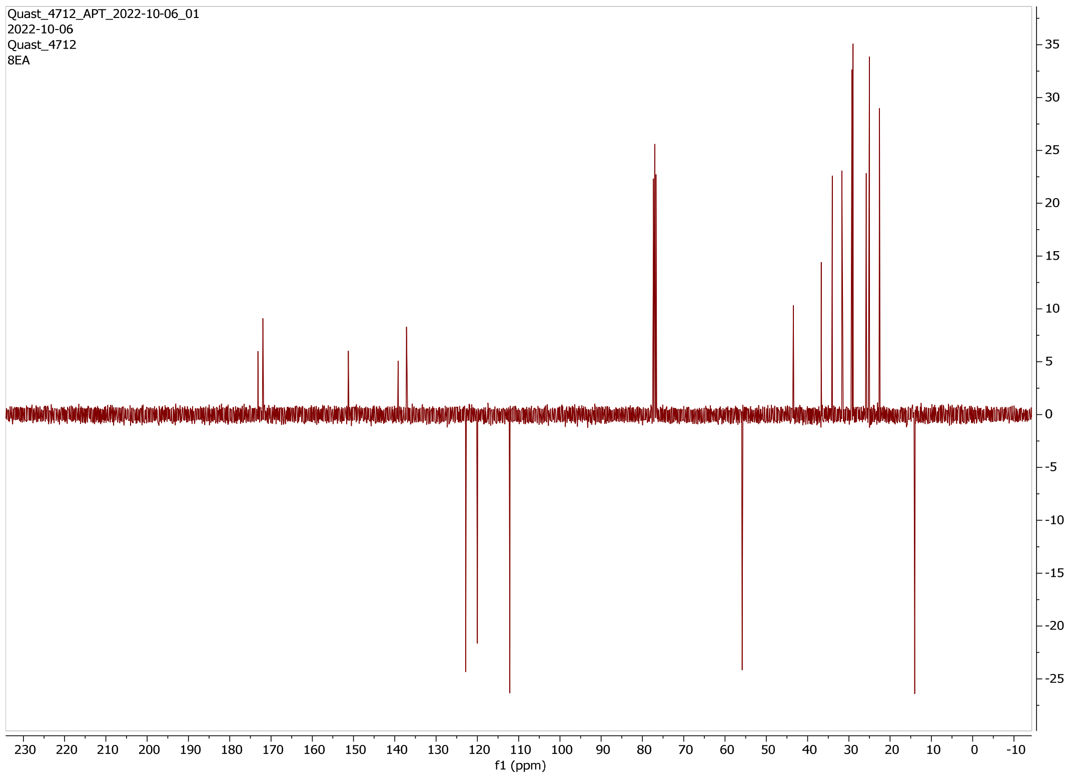

Compound **7**

^{1}H NMR Spectrum ($CDCl_3$, 400 MHz)

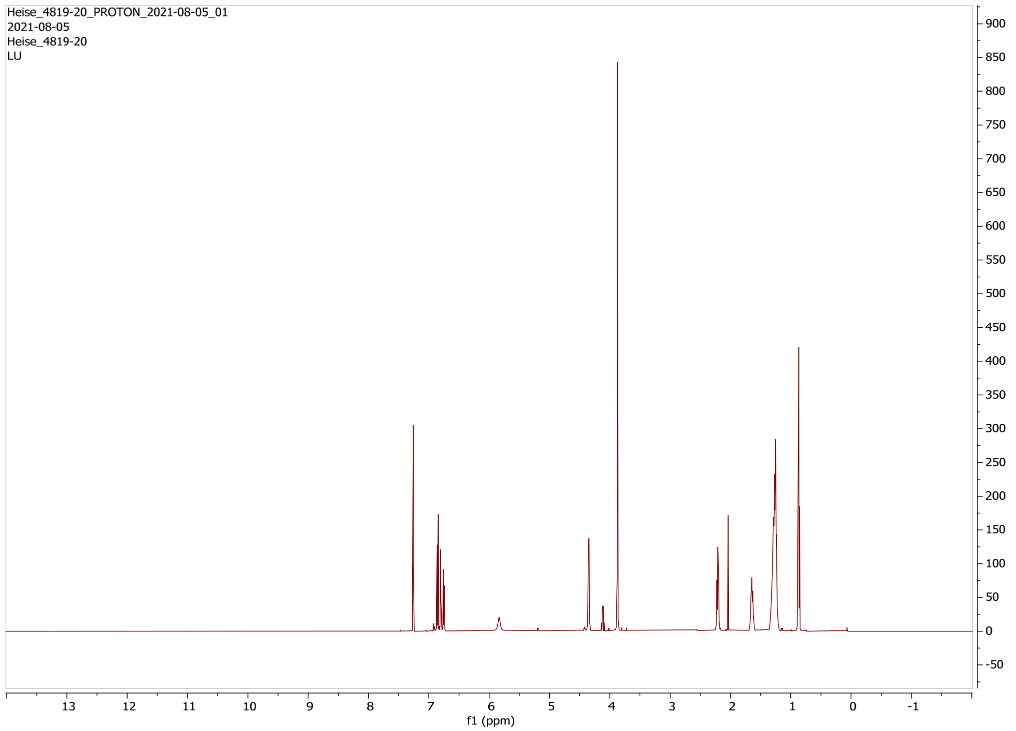

^{13}C-APT NMR Spectrum ($CDCl_3$, 101 MHz)

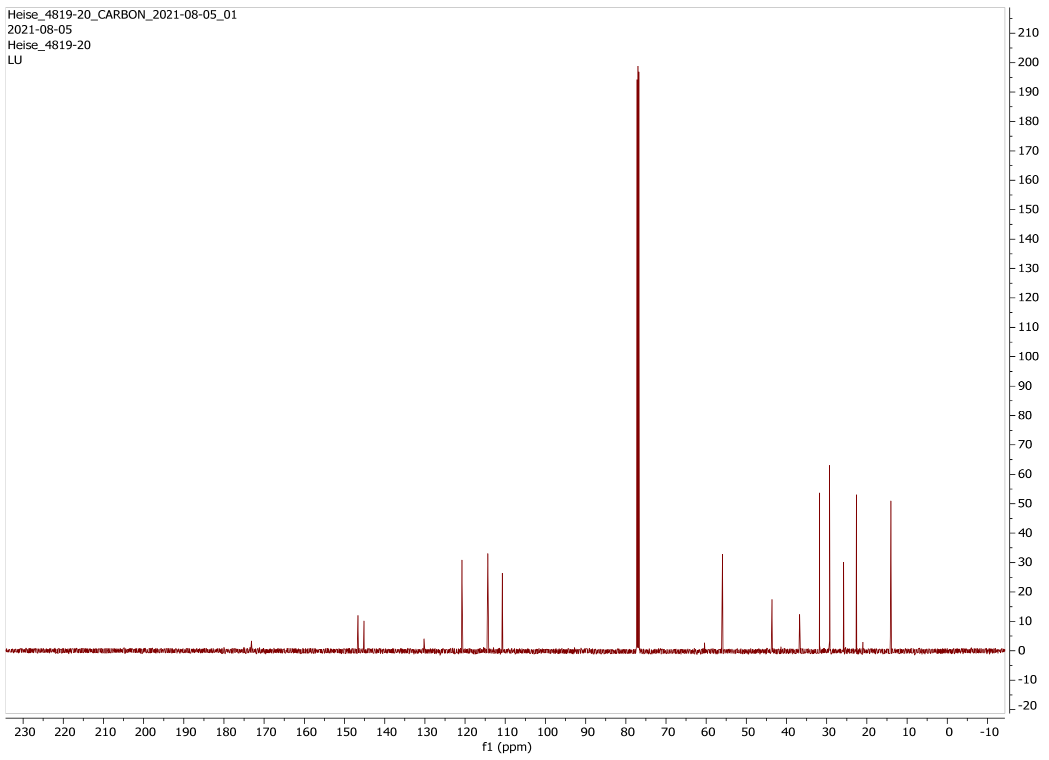

Compound **8**

^{1}H NMR Spectrum ($CDCl_3$, 400 MHz)

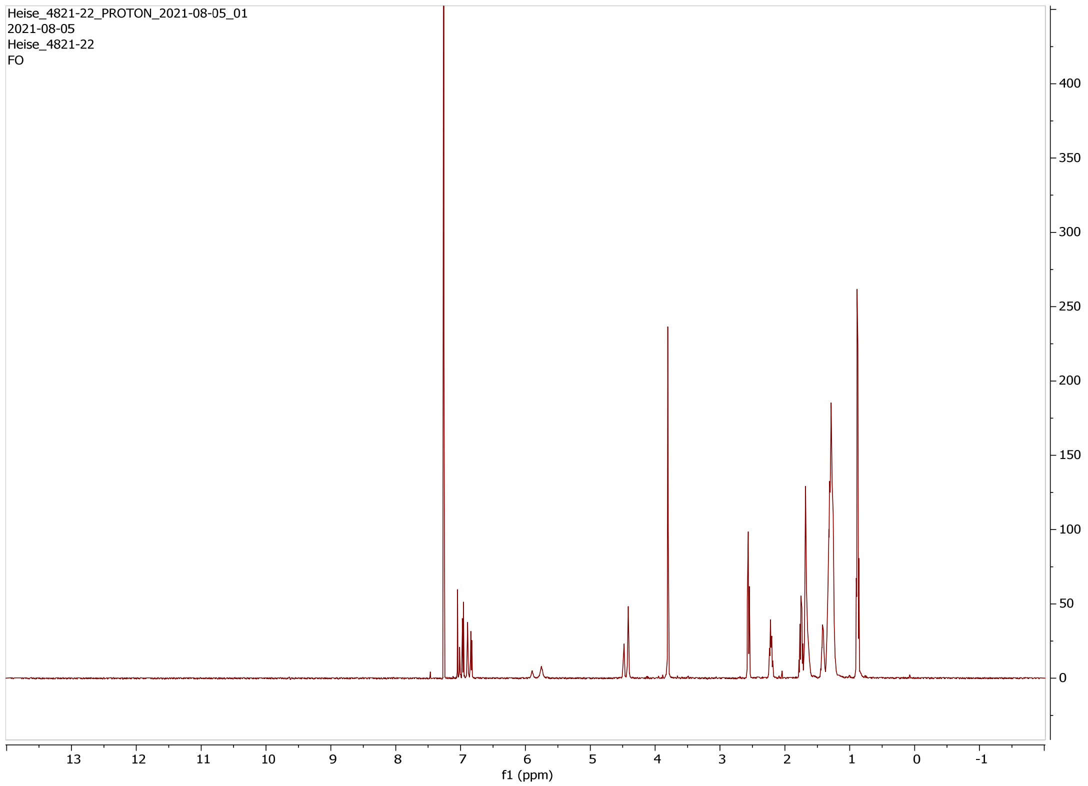

^{13}C-APT NMR Spectrum ($CDCl_3$, 101 MHz)

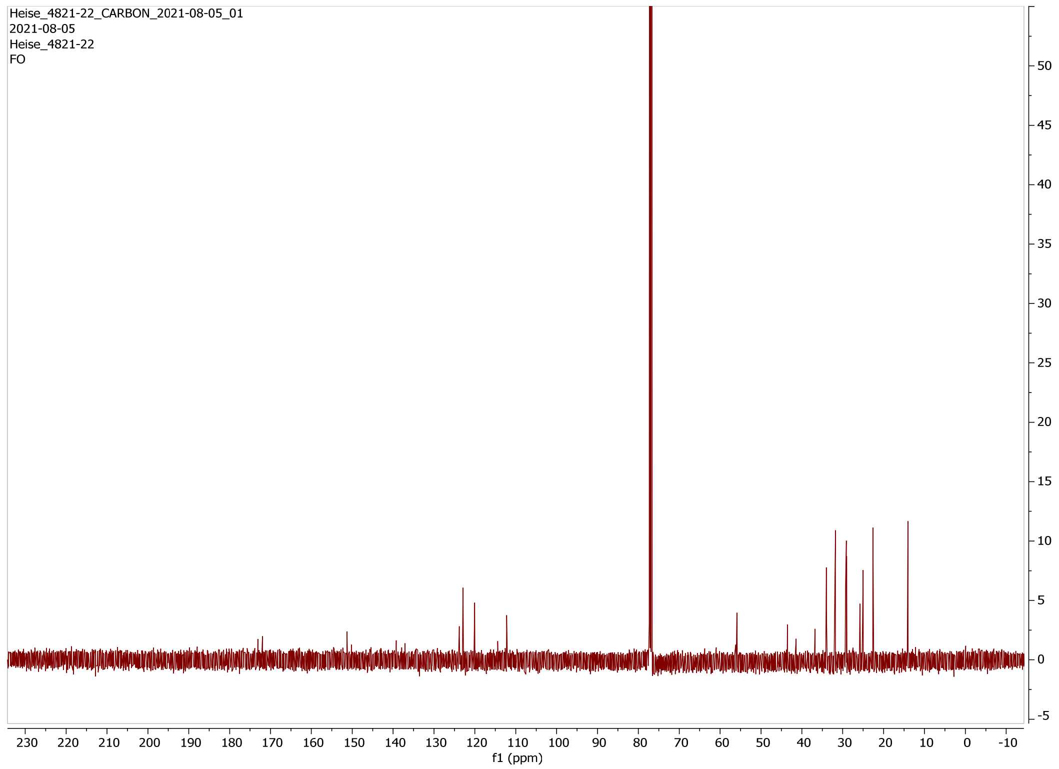

Compound **9**

^{1}H NMR Spectrum ($CDCl_3$, 400 MHz)

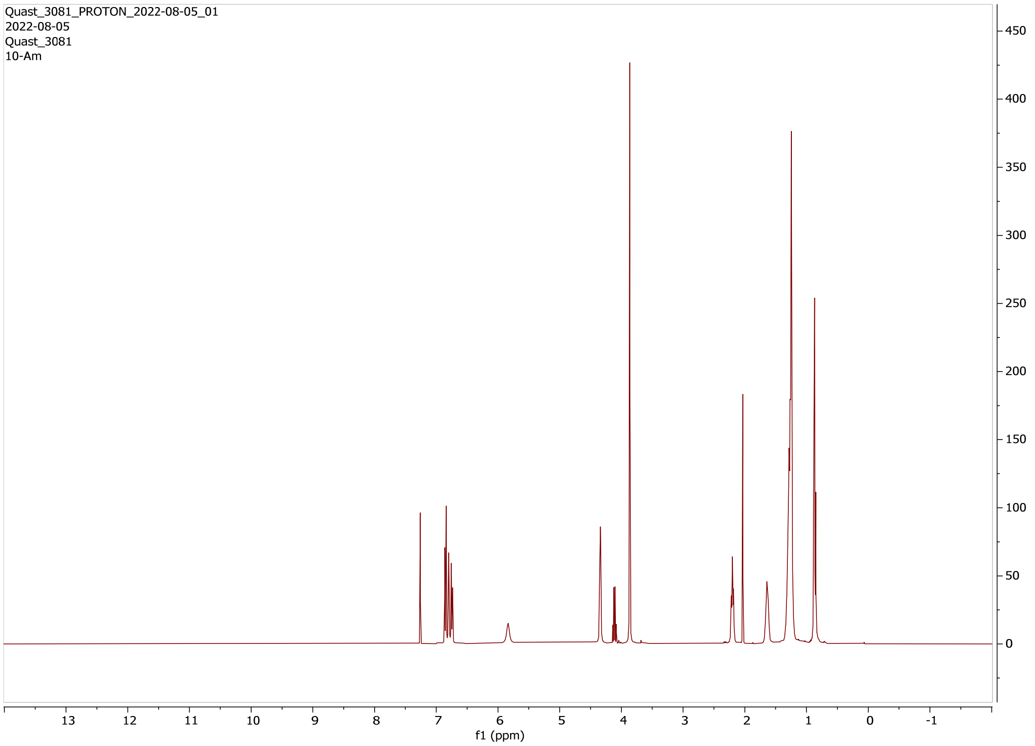

^{13}C-APT NMR Spectrum ($CDCl_3$, 101 MHz)

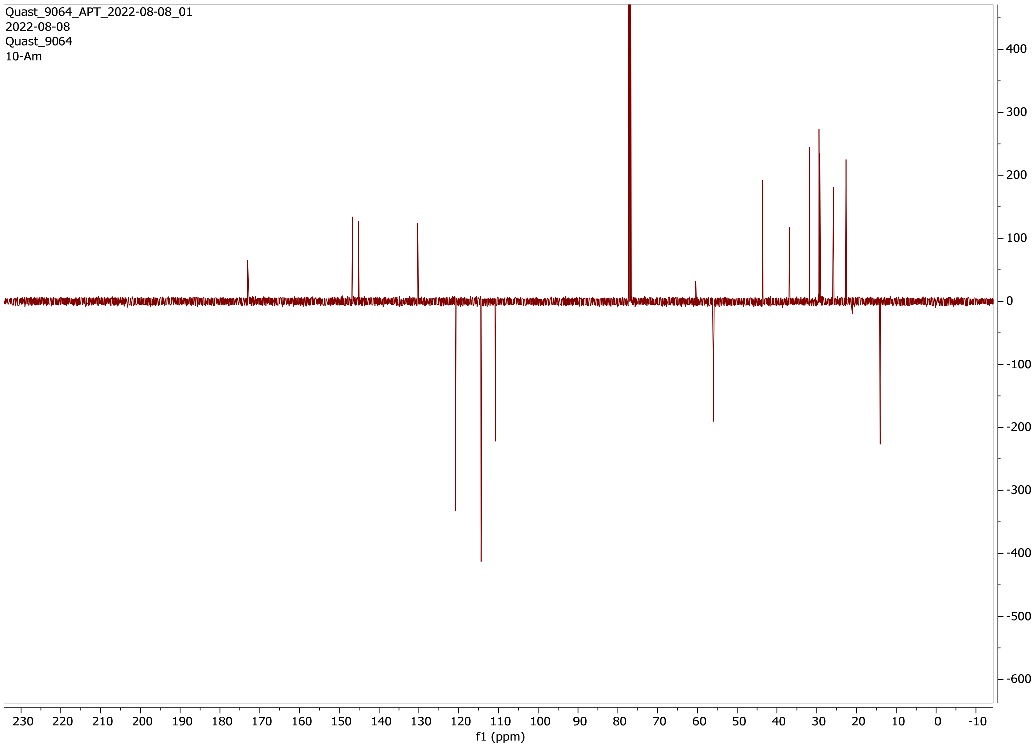

Compound **10**

^{1}H NMR Spectrum ($CDCl_3$, 400 MHz)

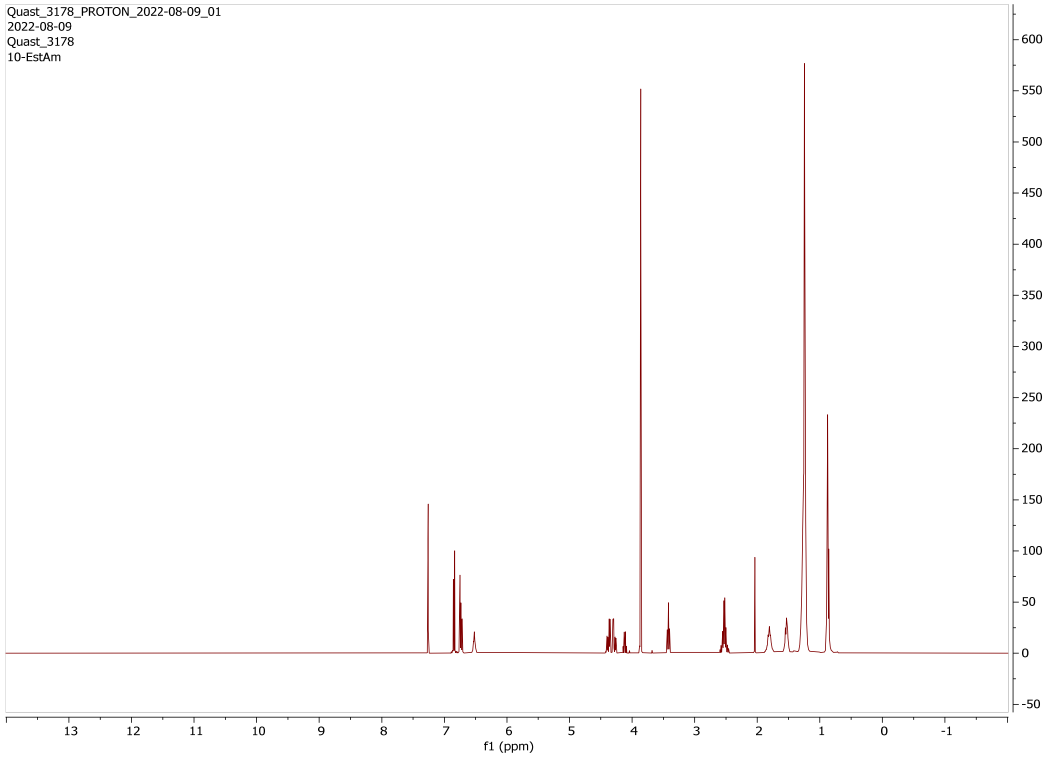

^{13}C-APT NMR Spectrum ($CDCl_3$, 101 MHz)

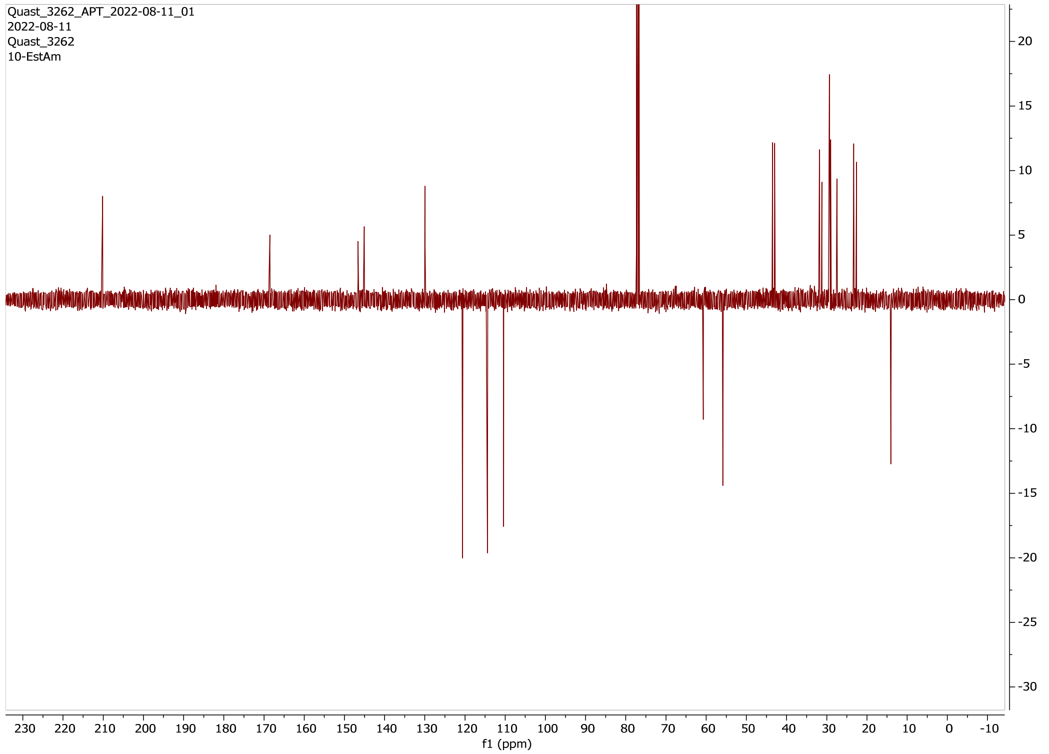

Compound **11**

^{1}H NMR Spectrum ($CDCl_3$, 400 MHz)

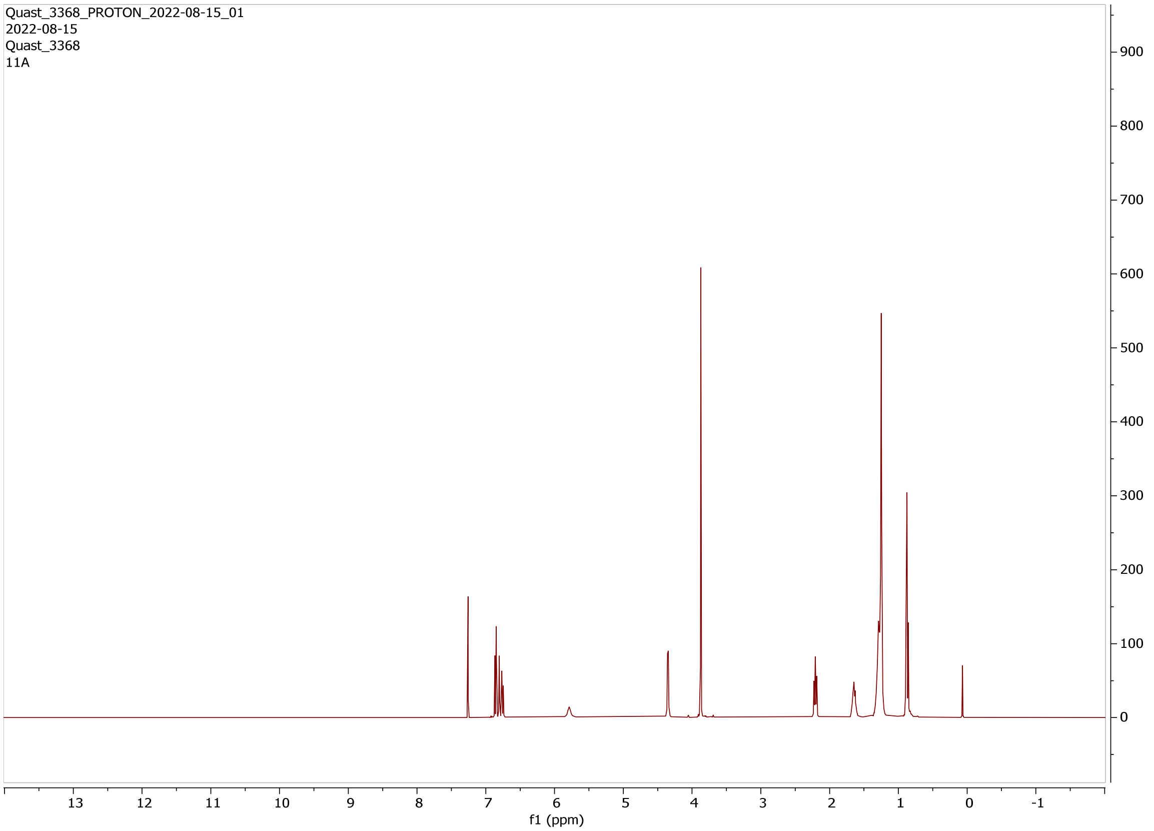

^{13}C-APT NMR Spectrum ($CDCl_3$, 101 MHz)

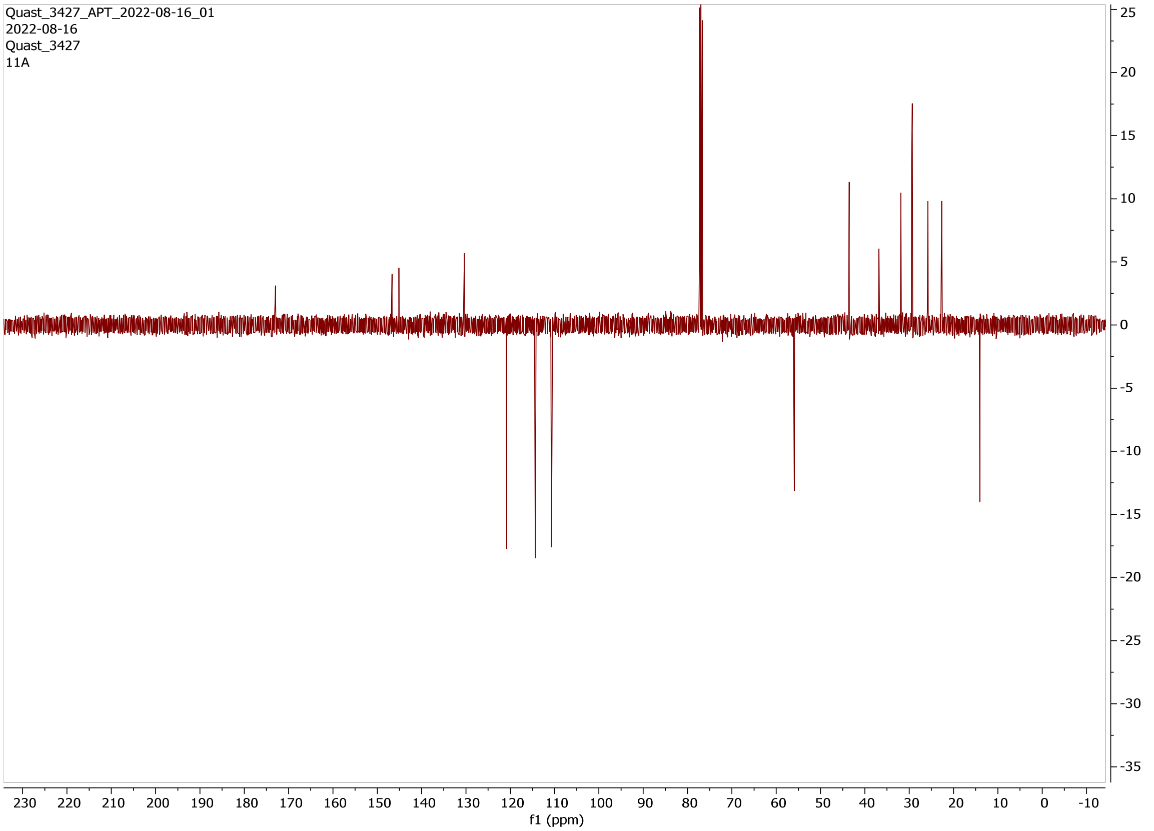

Compound **12**

^{1}H NMR Spectrum ($CDCl_3$, 400 MHz)

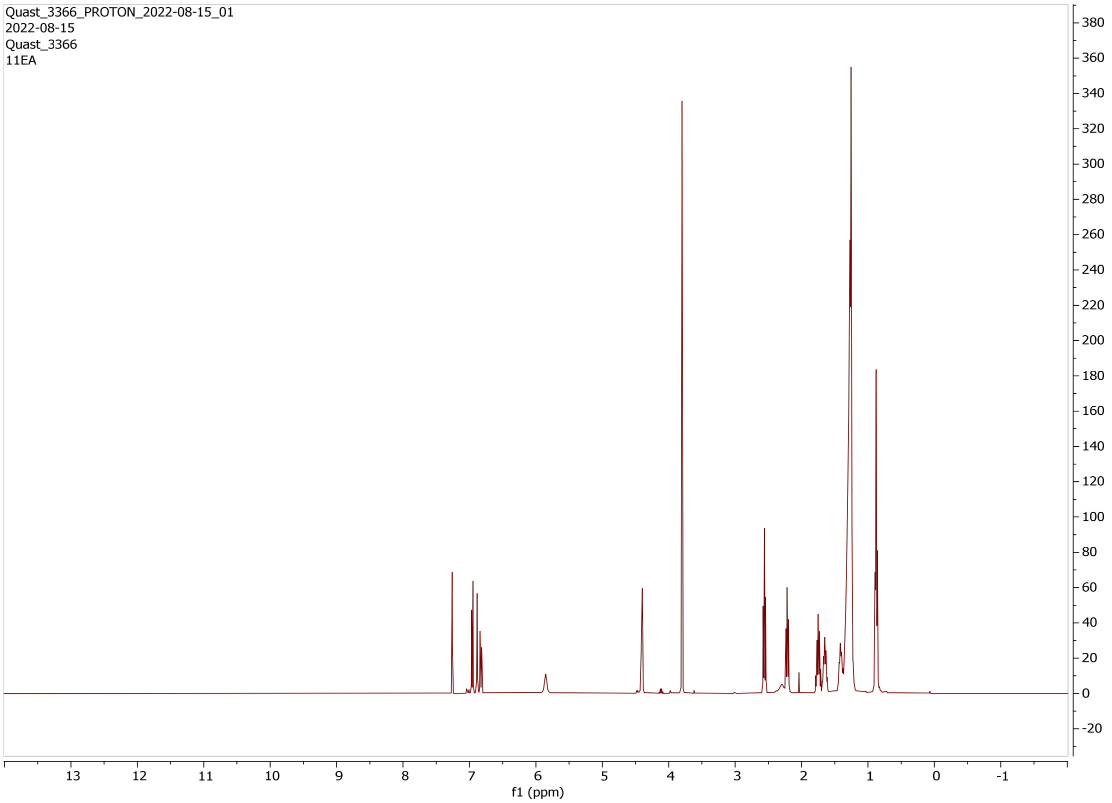

^{13}C-APT NMR Spectrum ($CDCl_3$, 101 MHz)

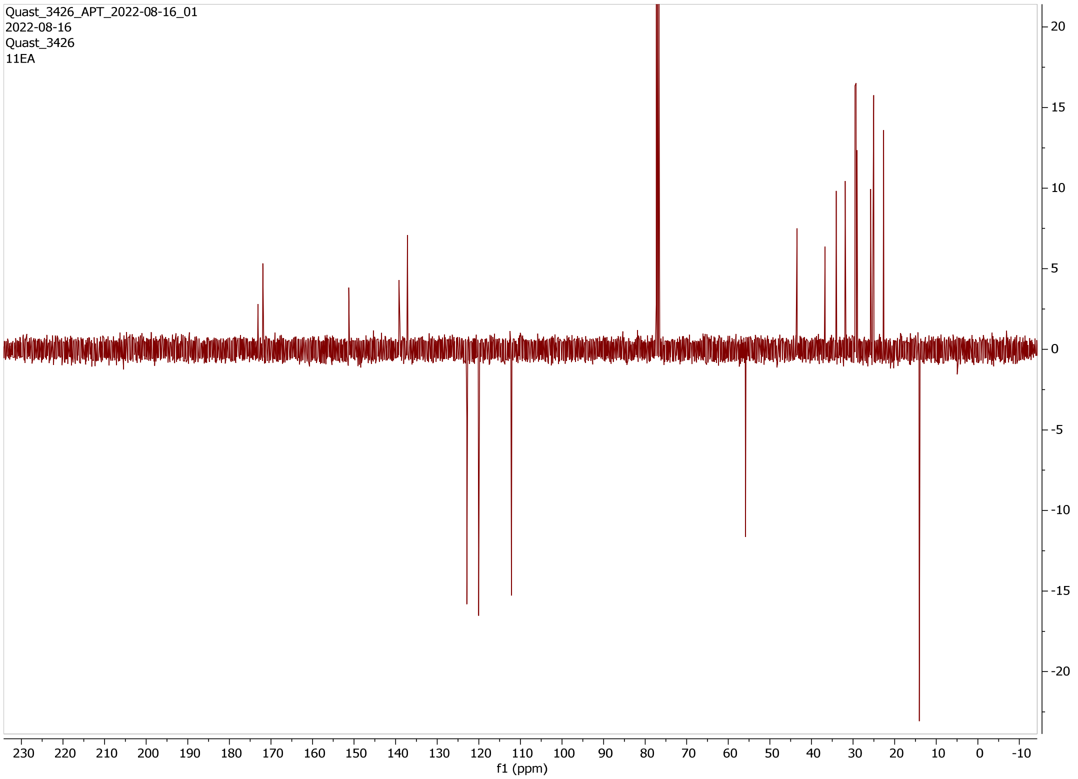

Compound **13**

^{1}H NMR Spectrum ($CDCl_3$, 400 MHz)

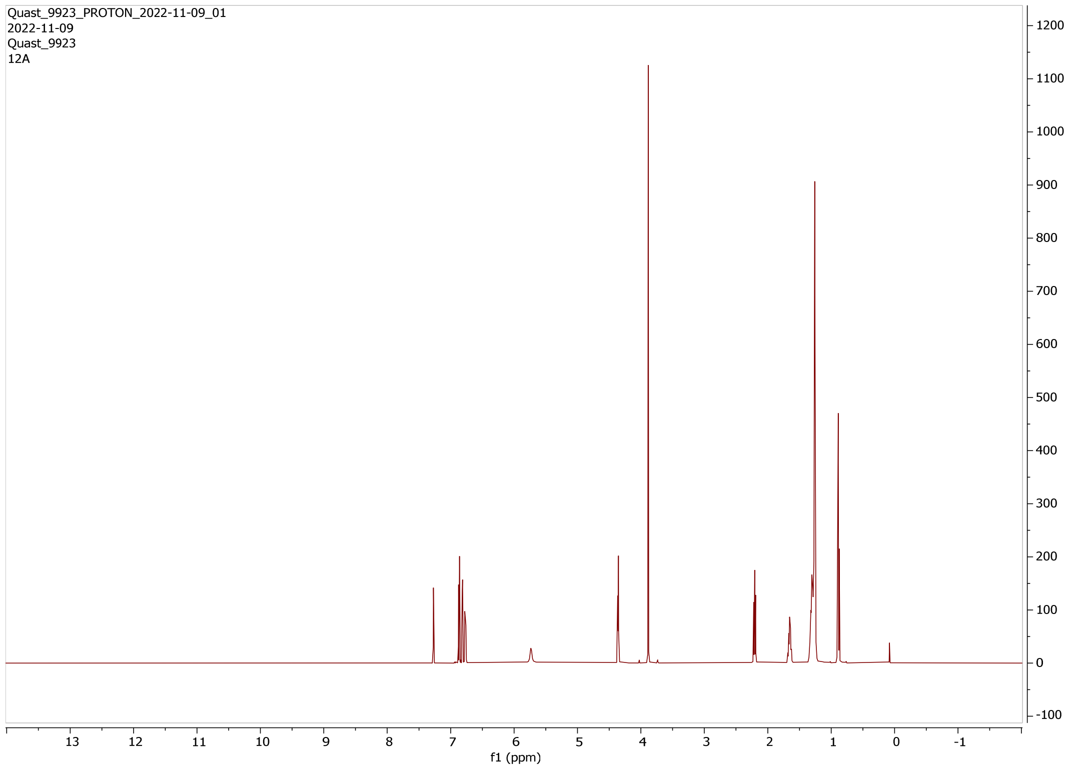

^{13}C-APT NMR Spectrum ($CDCl_3$, 101 MHz)

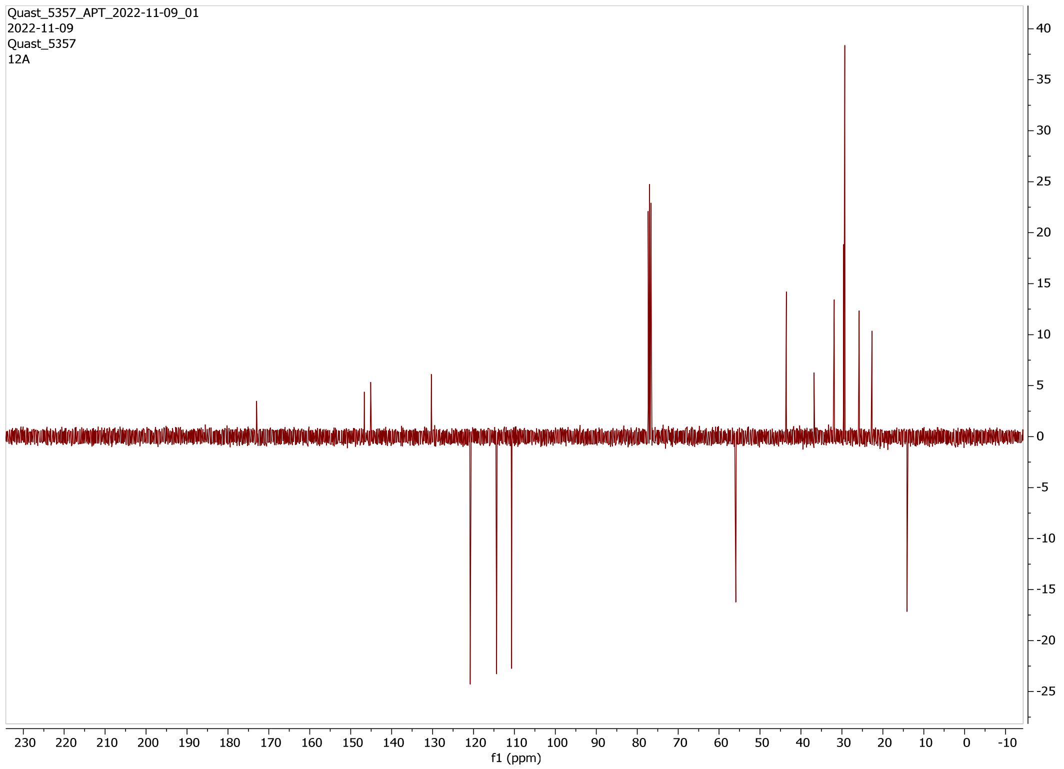

Compound **14**

^{1}H NMR Spectrum ($CDCl_3$, 400 MHz)

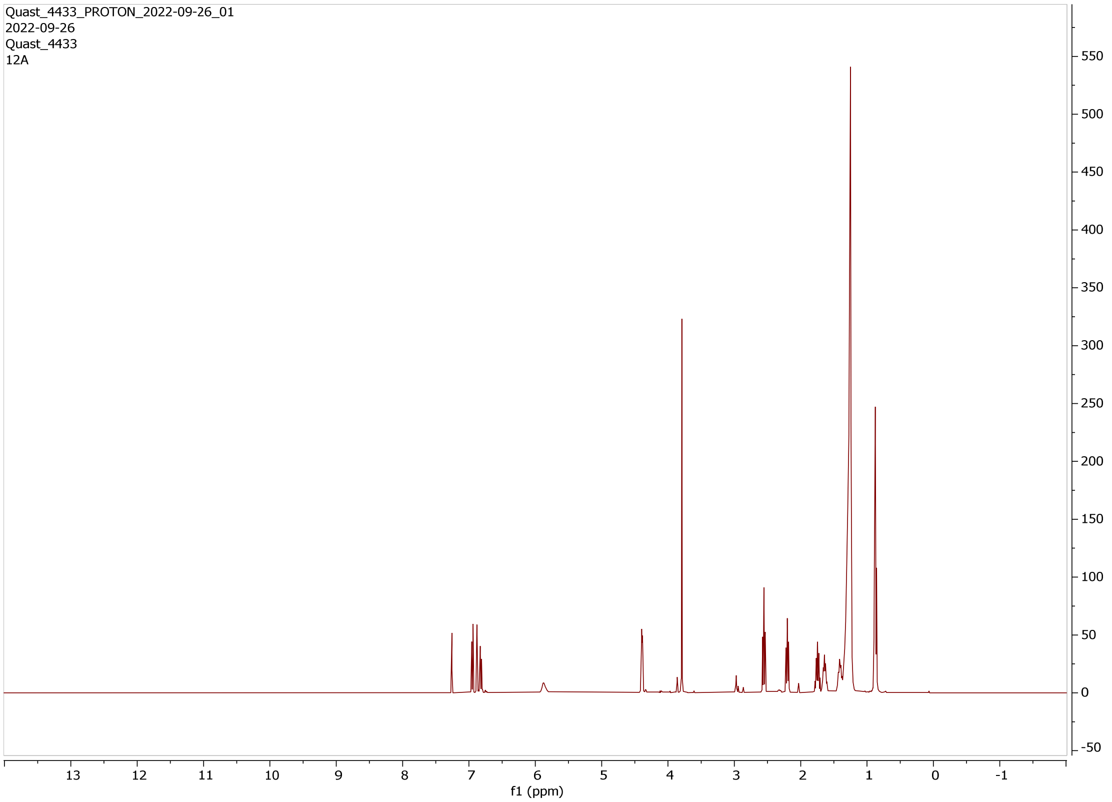

^{13}C-APT NMR Spectrum ($CDCl_3$, 101 MHz)

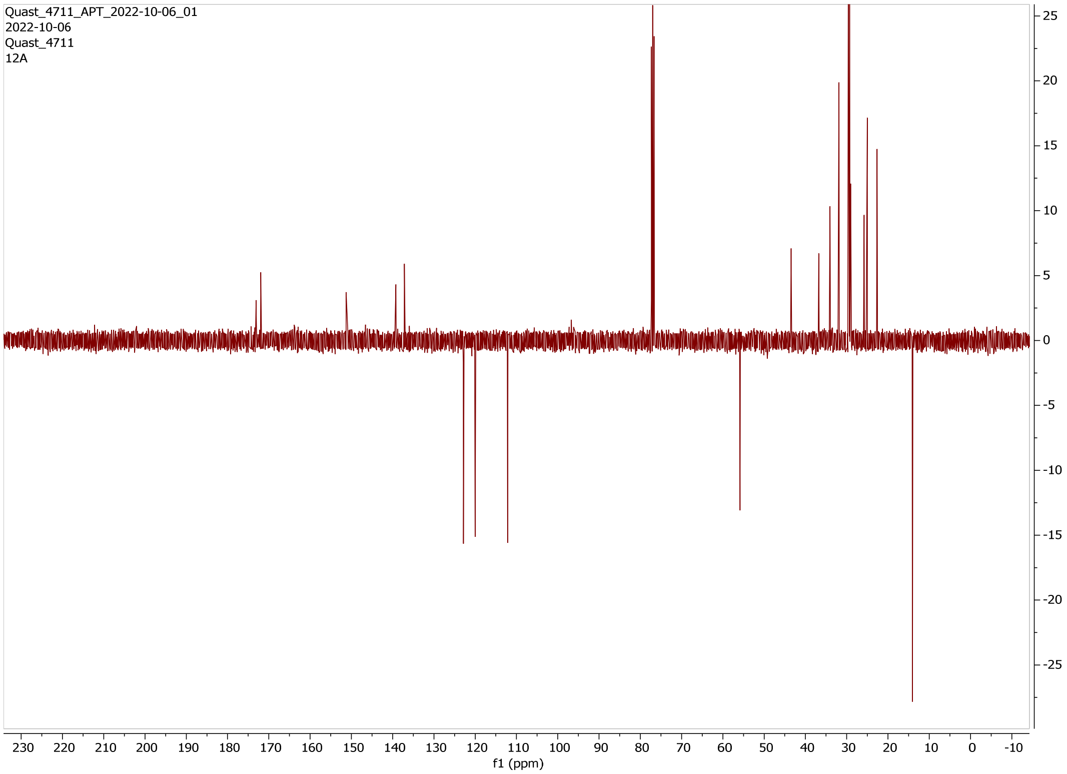

Compound **15**

^{1}H NMR Spectrum ($CDCl_3$, 400 MHz)

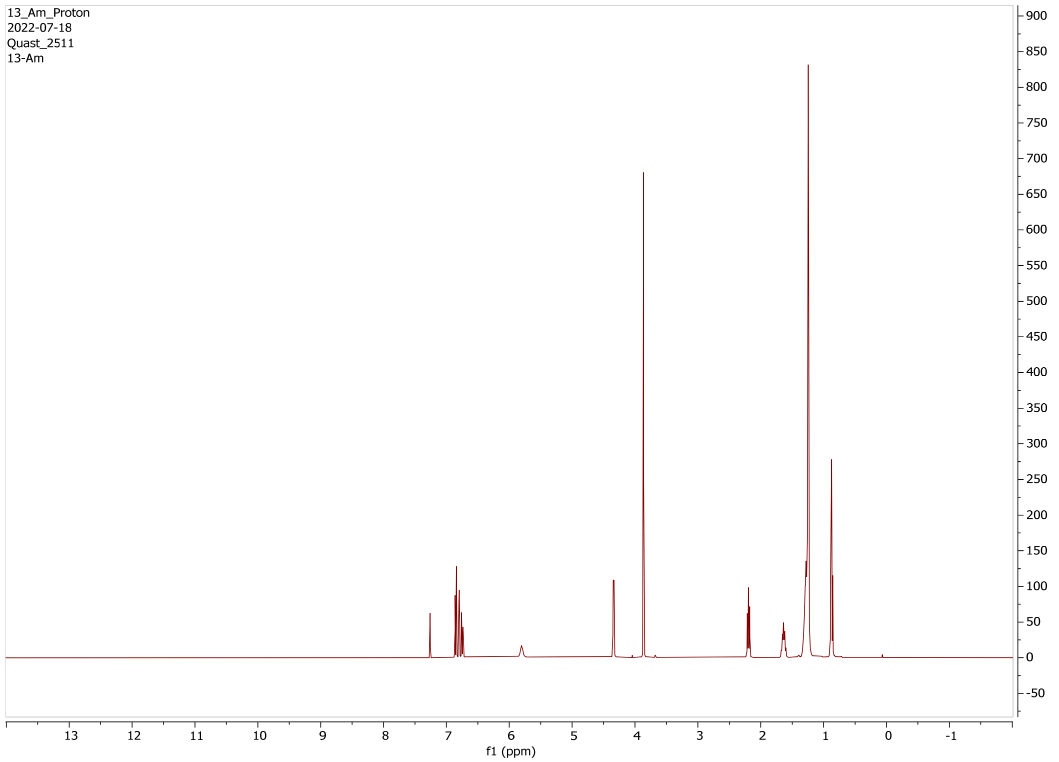

^{13}C-APT NMR Spectrum ($CDCl_3$, 101 MHz)

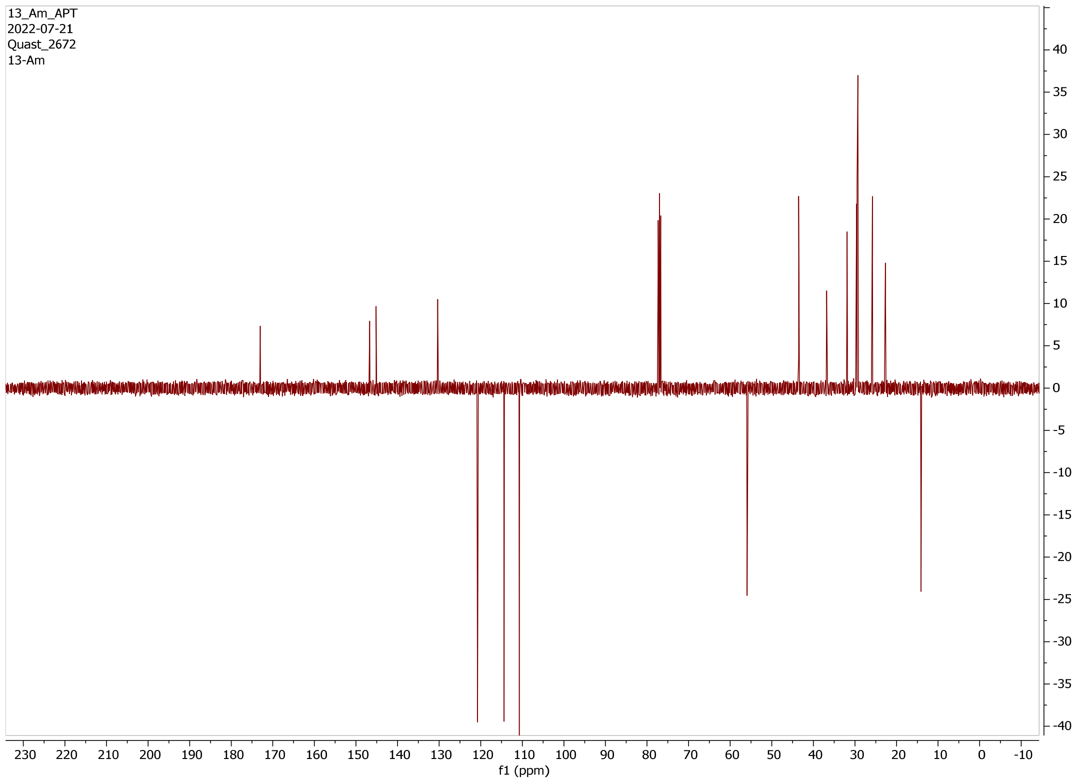

Compound **16**

^{1}H NMR Spectrum ($CDCl_3$, 400 MHz)

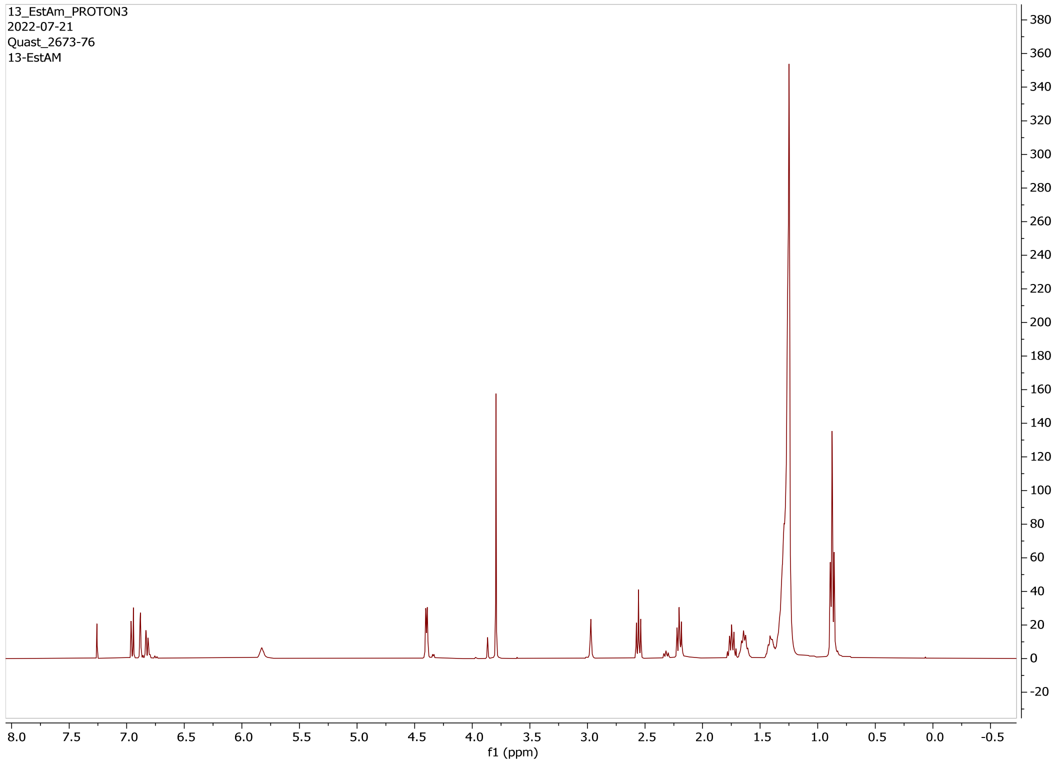

^{13}C-APT NMR Spectrum ($CDCl_3$, 101 MHz)

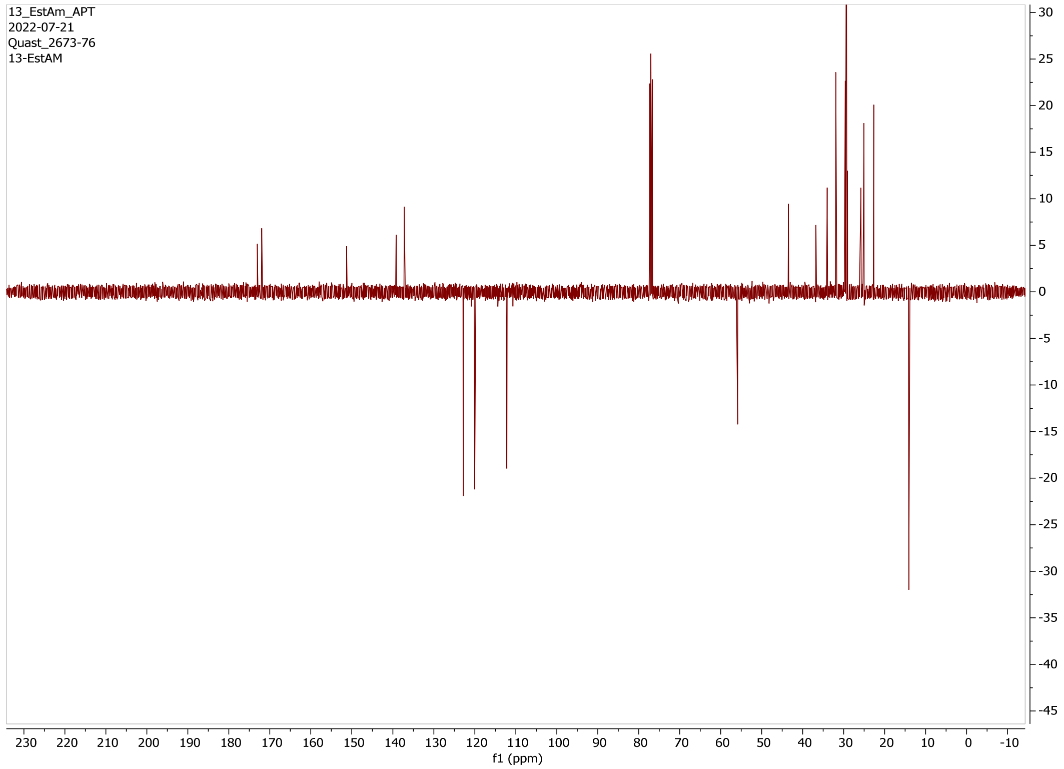

Compound **17**

^{1}H NMR Spectrum ($CDCl_3$, 400 MHz)

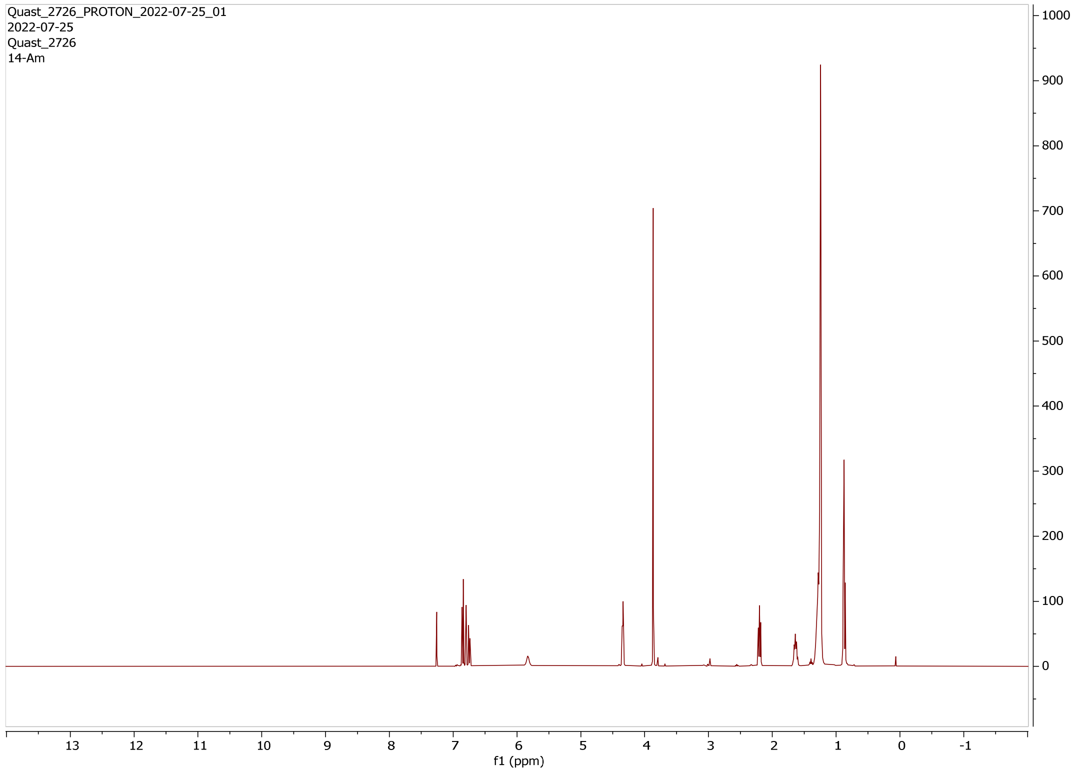

^{13}C-APT NMR Spectrum ($CDCl_3$, 101 MHz)

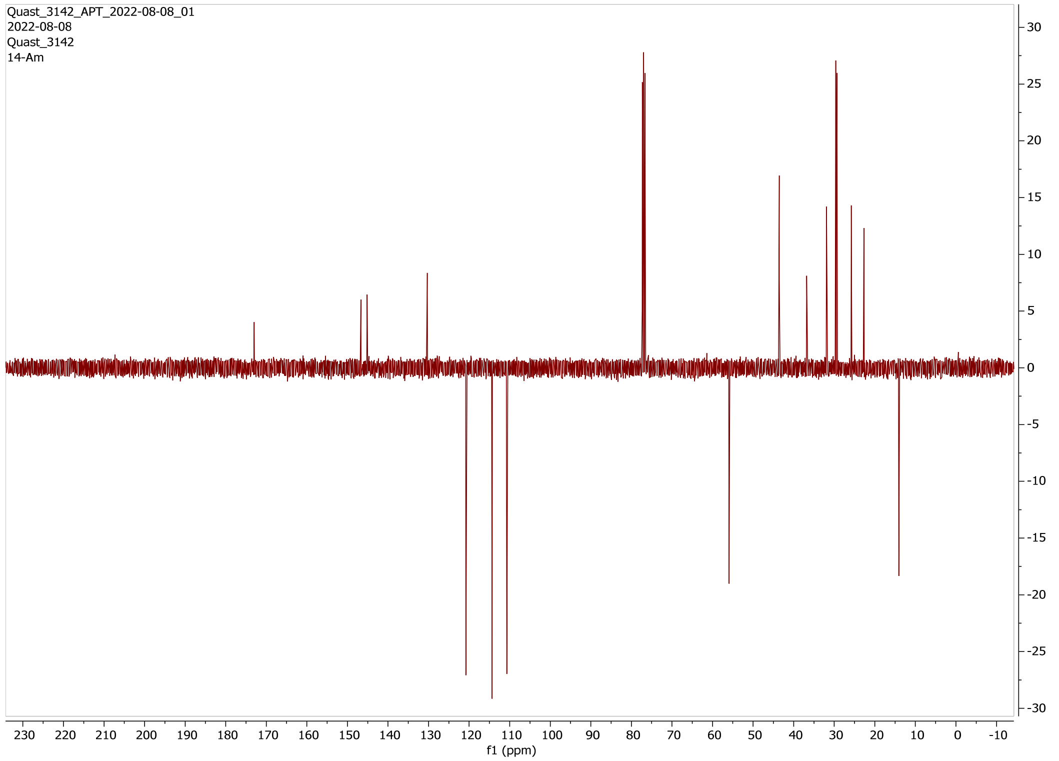

Compound **18**

^{1}H NMR Spectrum ($CDCl_3$, 400 MHz)

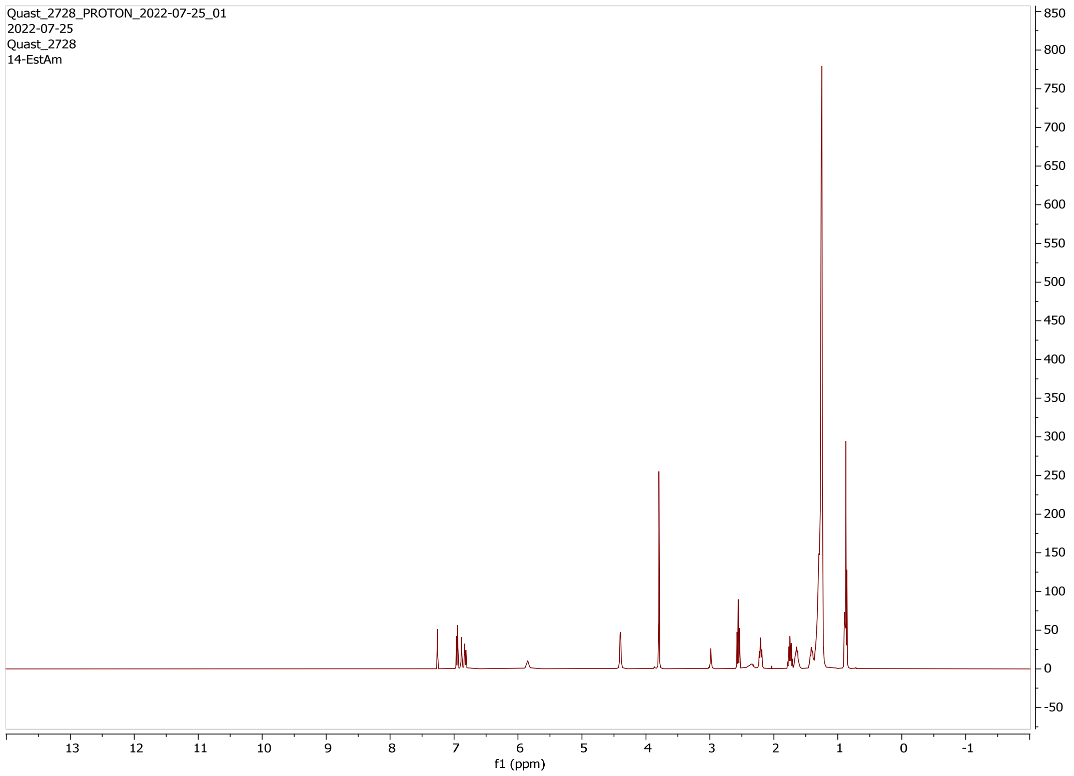

^{13}C-APT NMR Spectrum ($CDCl_3$, 101 MHz)

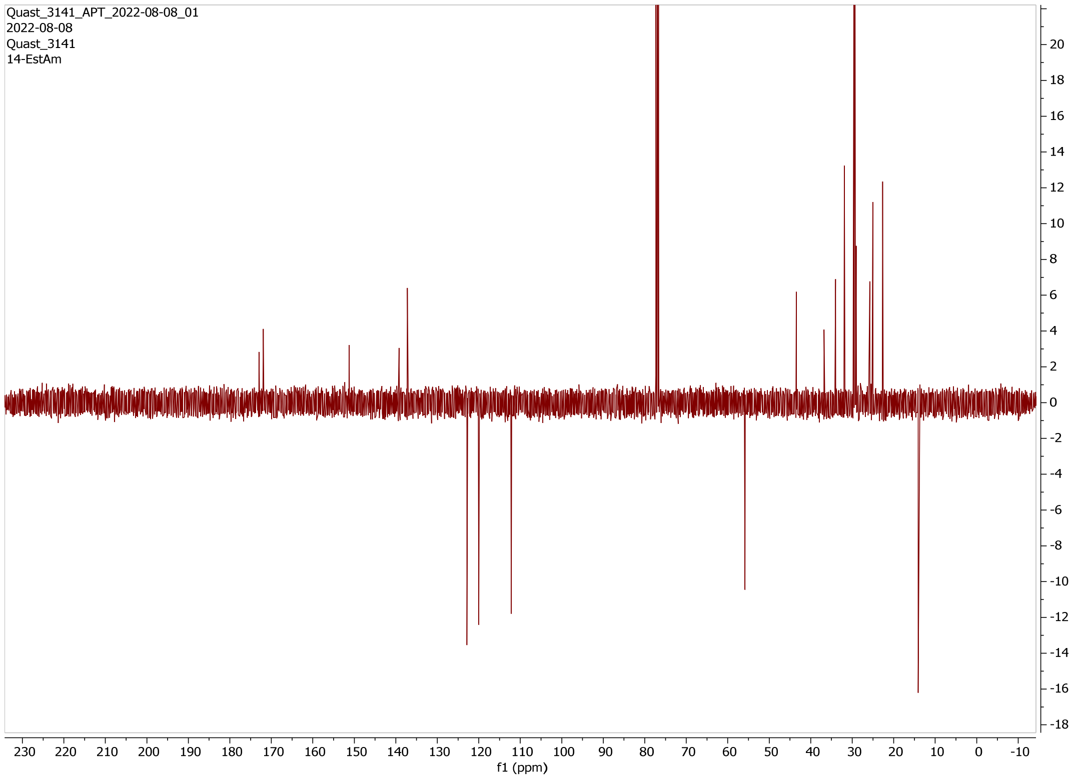

Compound **19**

^{1}H NMR Spectrum ($CDCl_3$, 400 MHz)

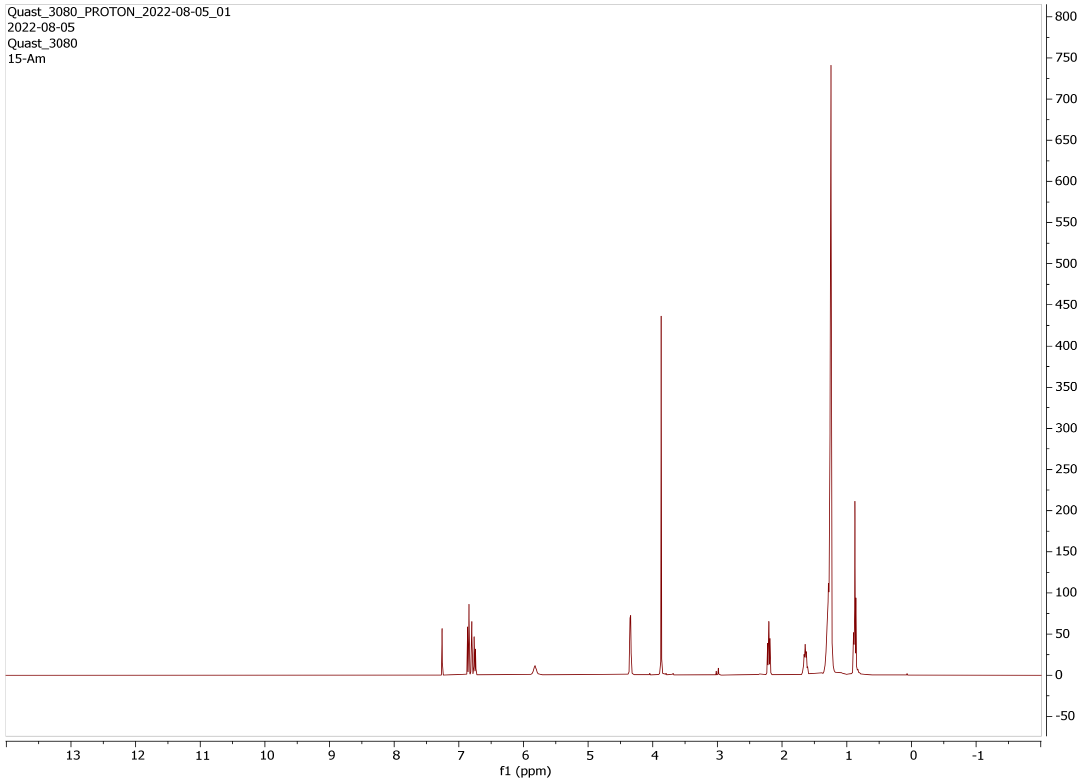

^{13}C-APT NMR Spectrum ($CDCl_3$, 101 MHz)

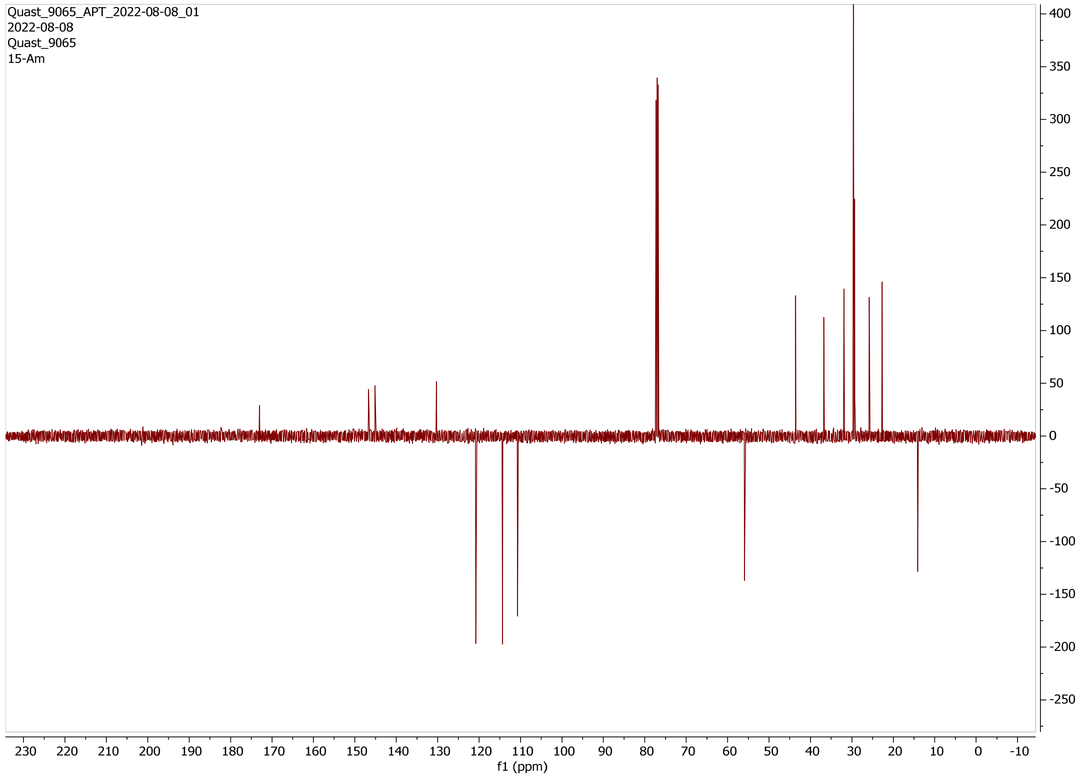

Compound **20**

^{1}H NMR Spectrum ($CDCl_3$, 400 MHz)

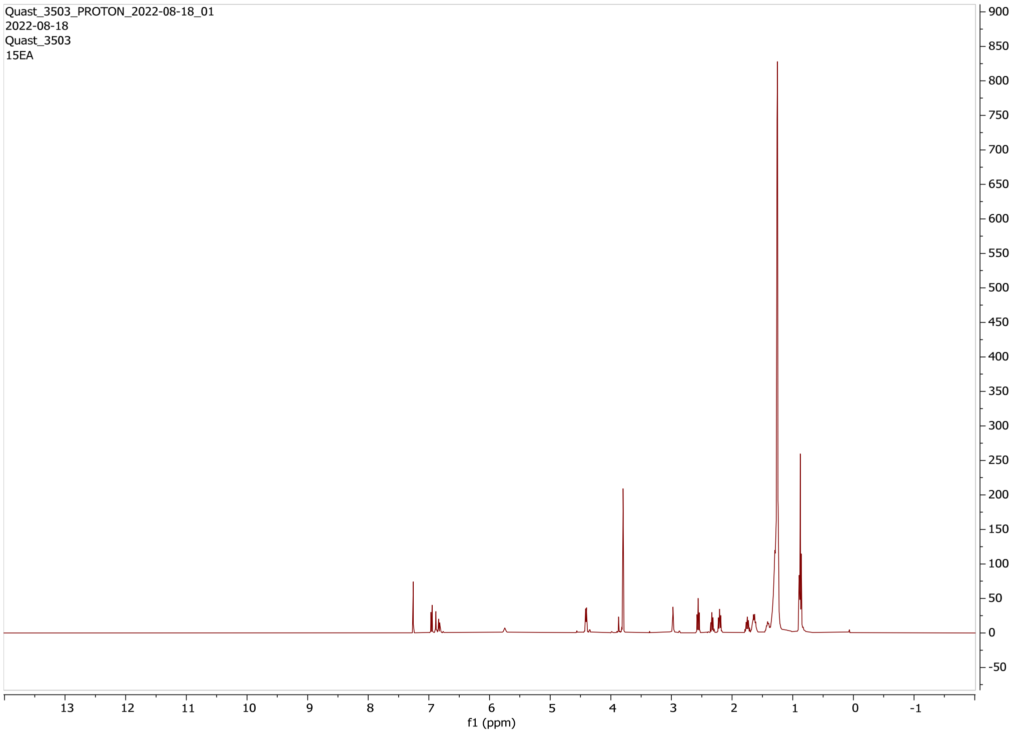

^{13}C-APT NMR Spectrum ($CDCl_3$, 101 MHz)

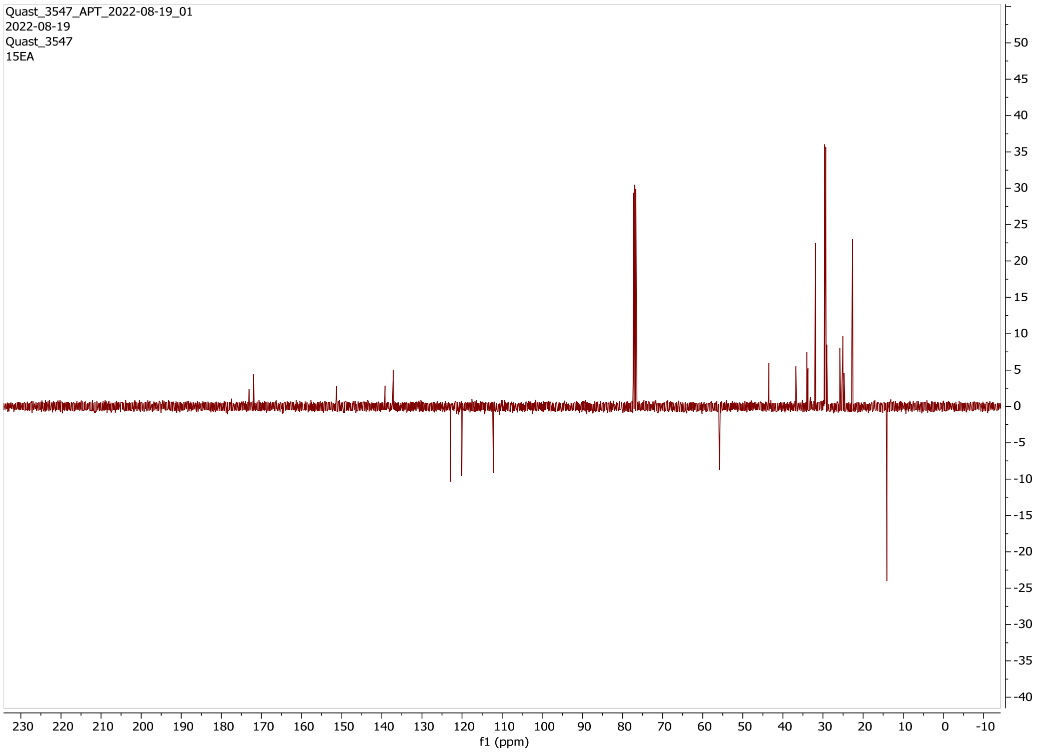

Compound **21**

^{1}H NMR Spectrum ($CDCl_3$, 400 MHz)

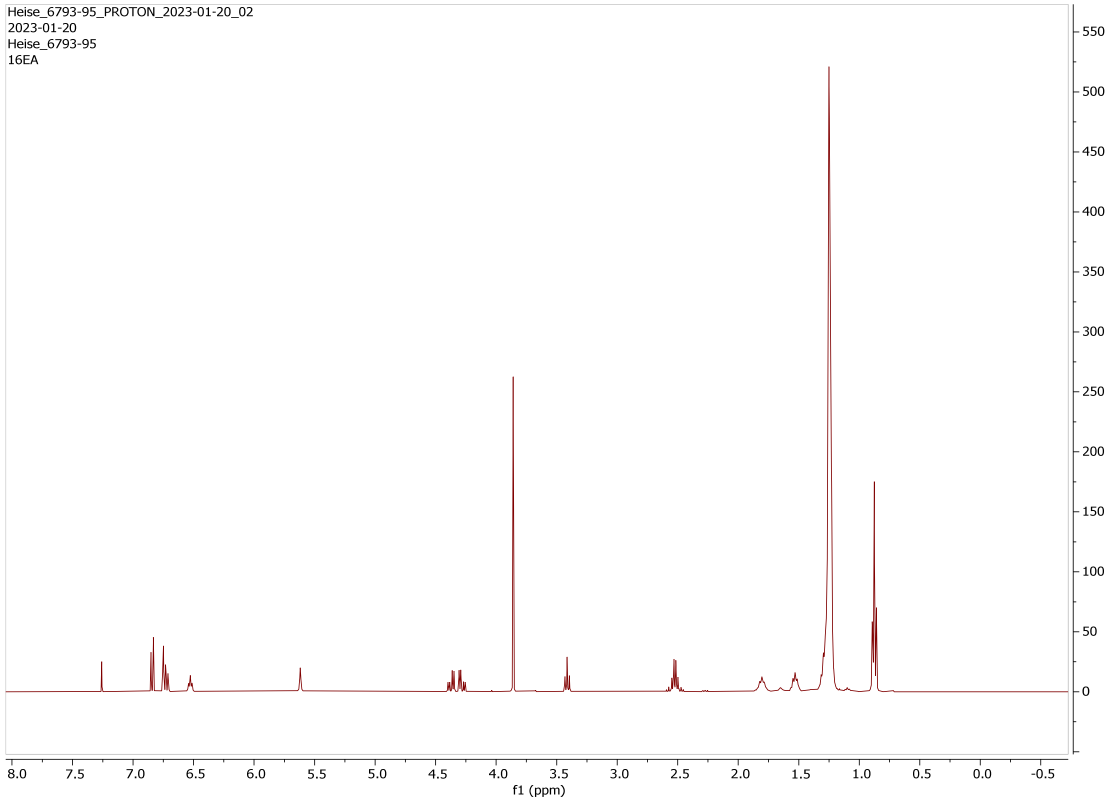

^{13}C-APT NMR Spectrum ($CDCl_3$, 101 MHz)

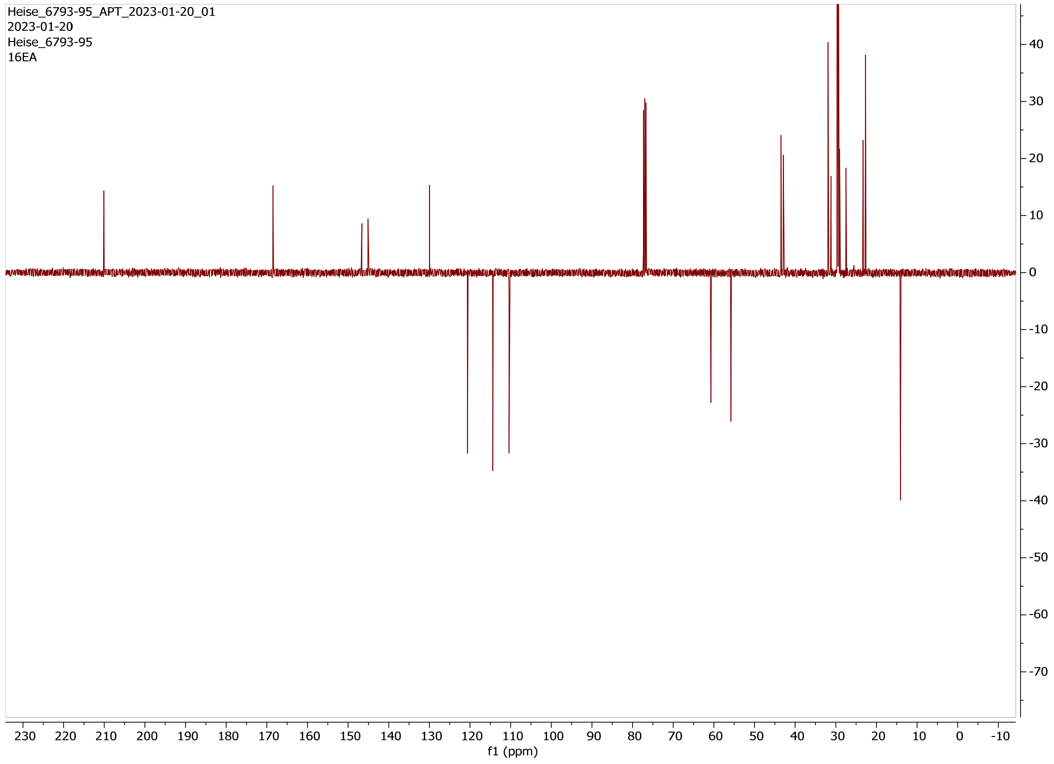

Compound **22**

^{1}H NMR Spectrum ($CDCl_3$, 400 MHz)

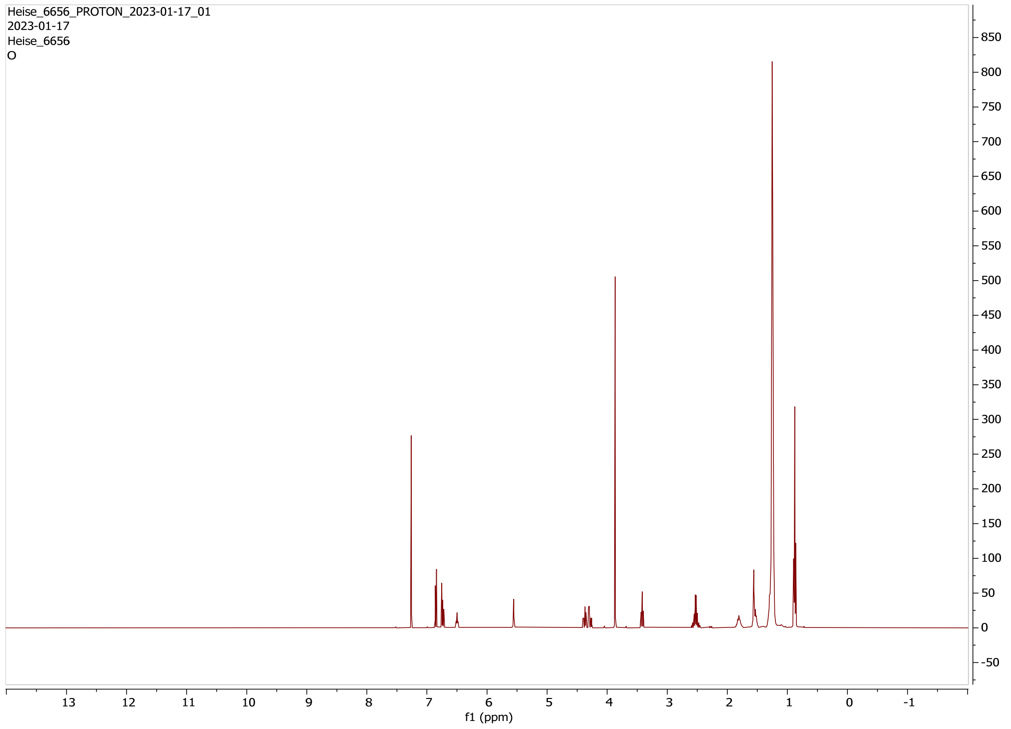

^{13}C-APT NMR Spectrum ($CDCl_3$, 101 MHz)

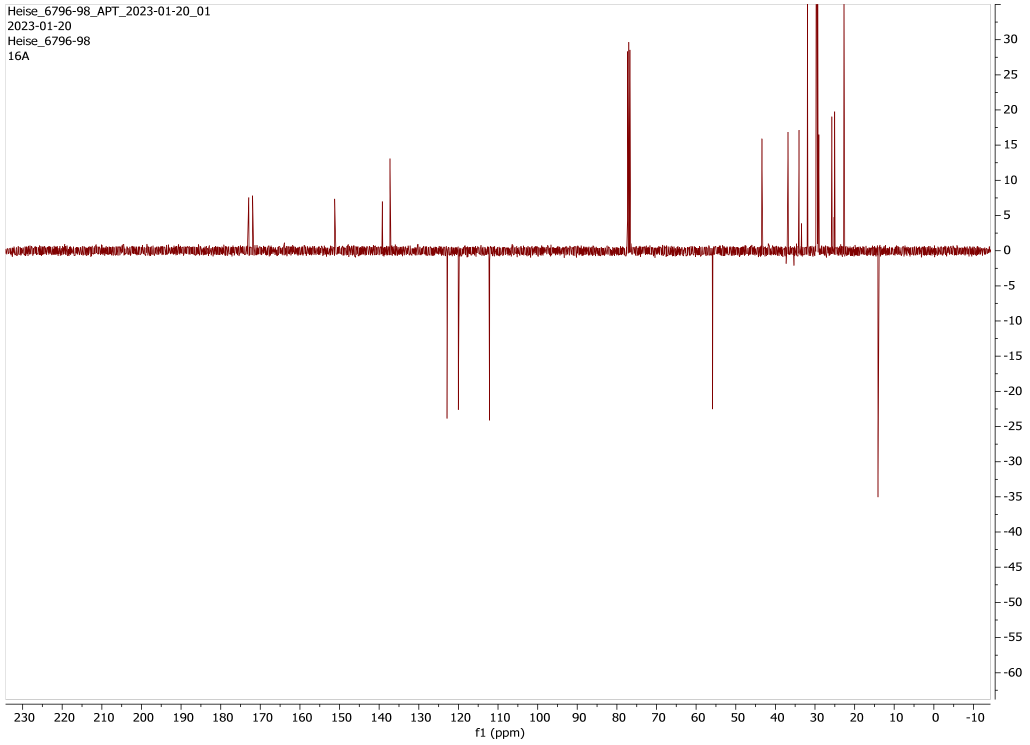

Compound **23**

^{1}H NMR Spectrum ($CDCl_3$, 400 MHz)

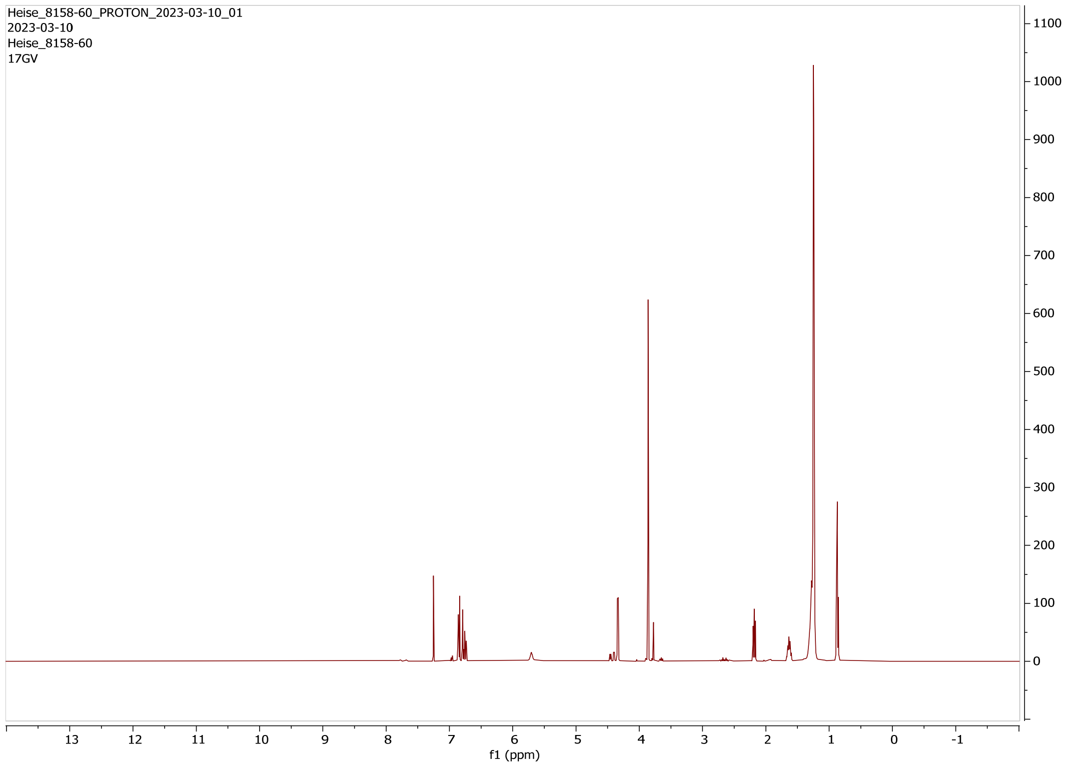

^{13}C-APT NMR Spectrum ($CDCl_3$, 101 MHz)

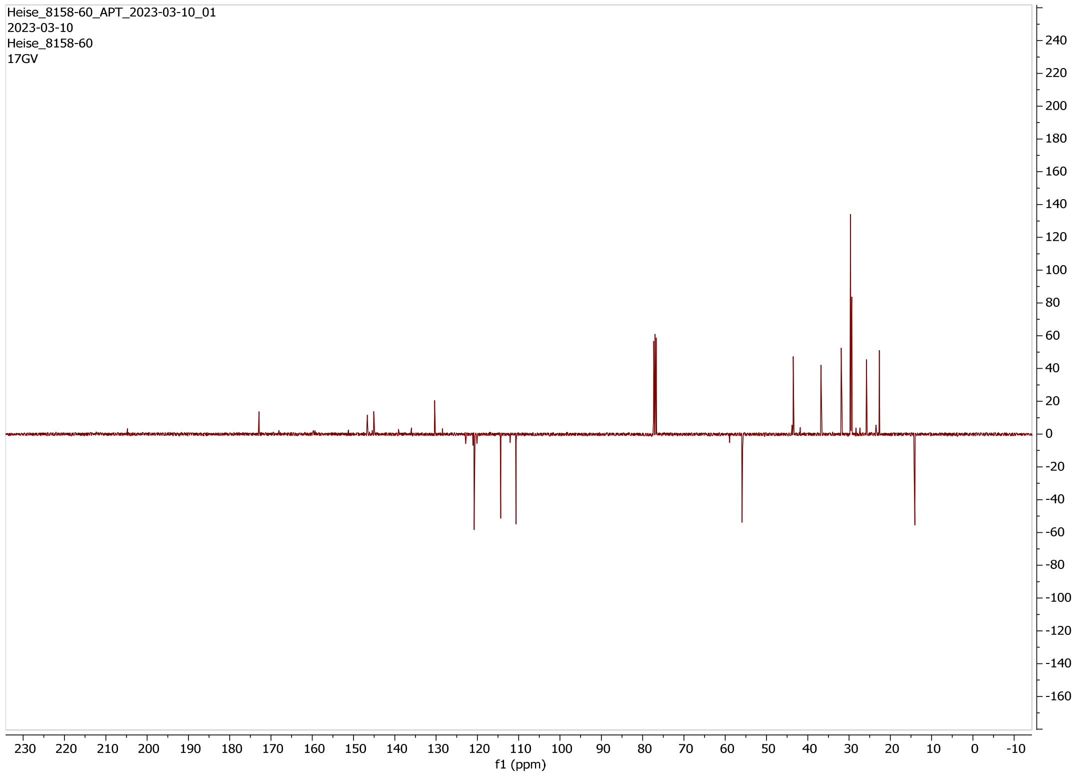

Compound **24**

^{1}H NMR Spectrum ($CDCl_3$, 400 MHz)

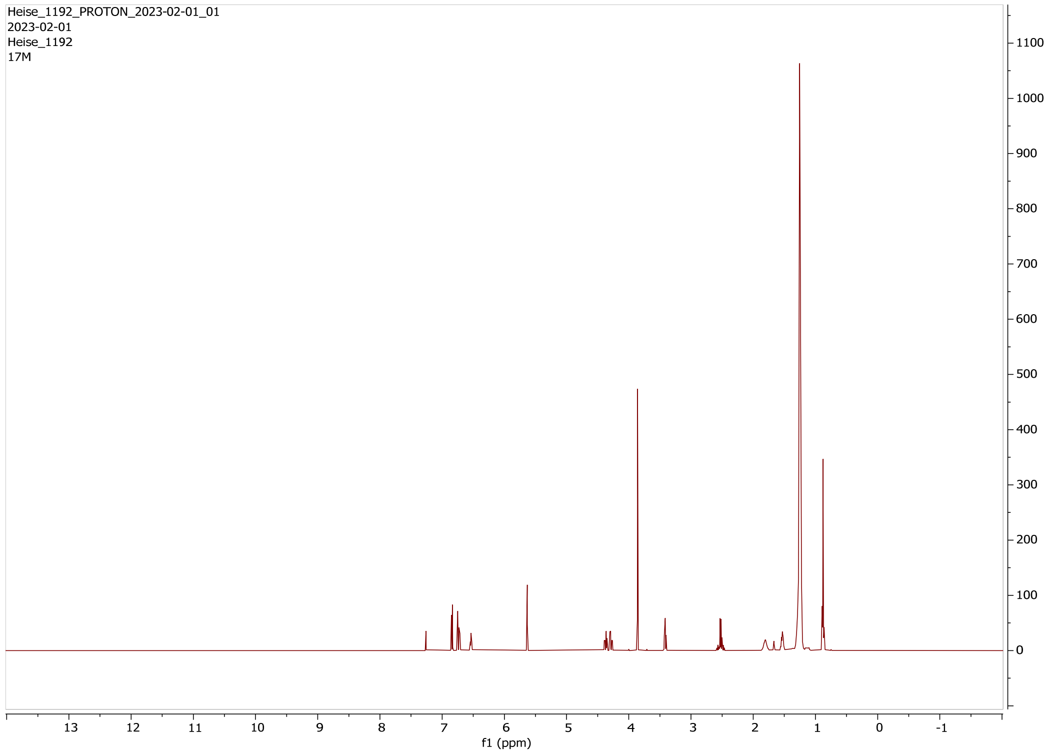

^{13}C-APT NMR Spectrum ($CDCl_3$, 101 MHz)

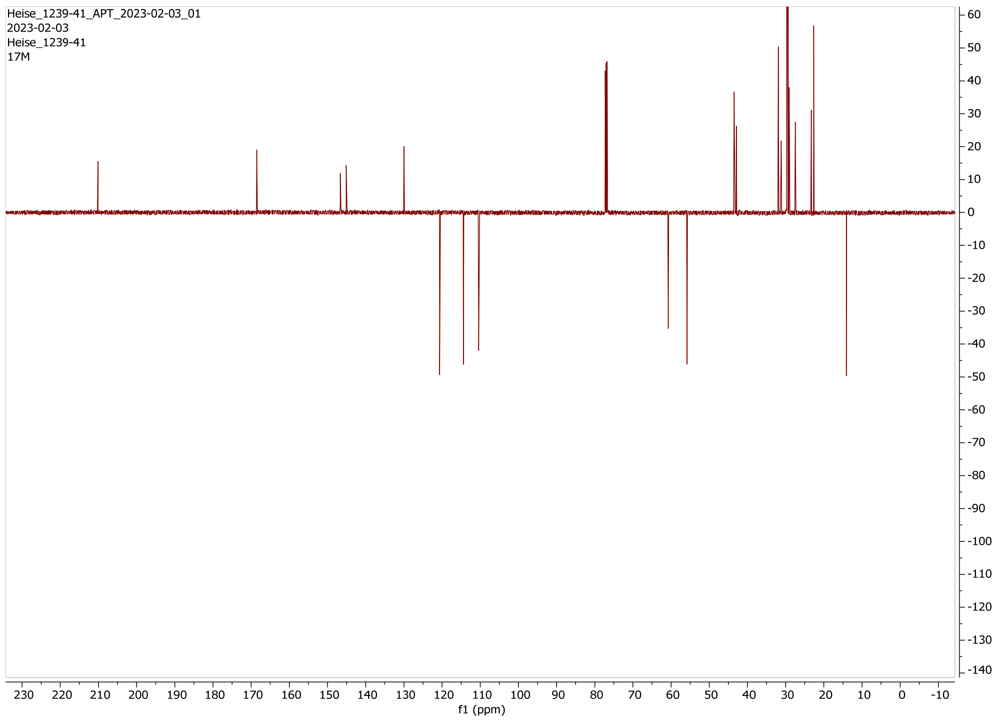

Compound **25**

^{1}H NMR Spectrum (CDCl$_3$, 400 MHz)

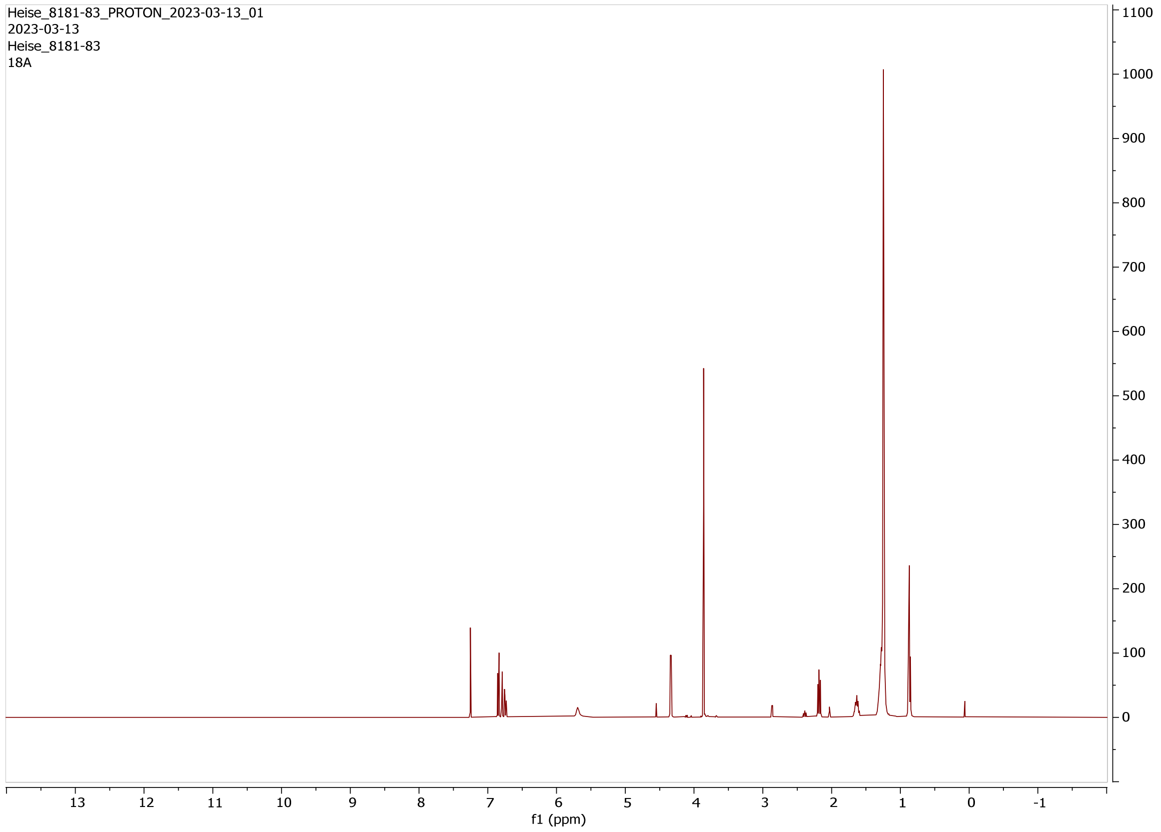

^{13}C-APT NMR Spectrum (CDCl$_3$, 101 MHz)

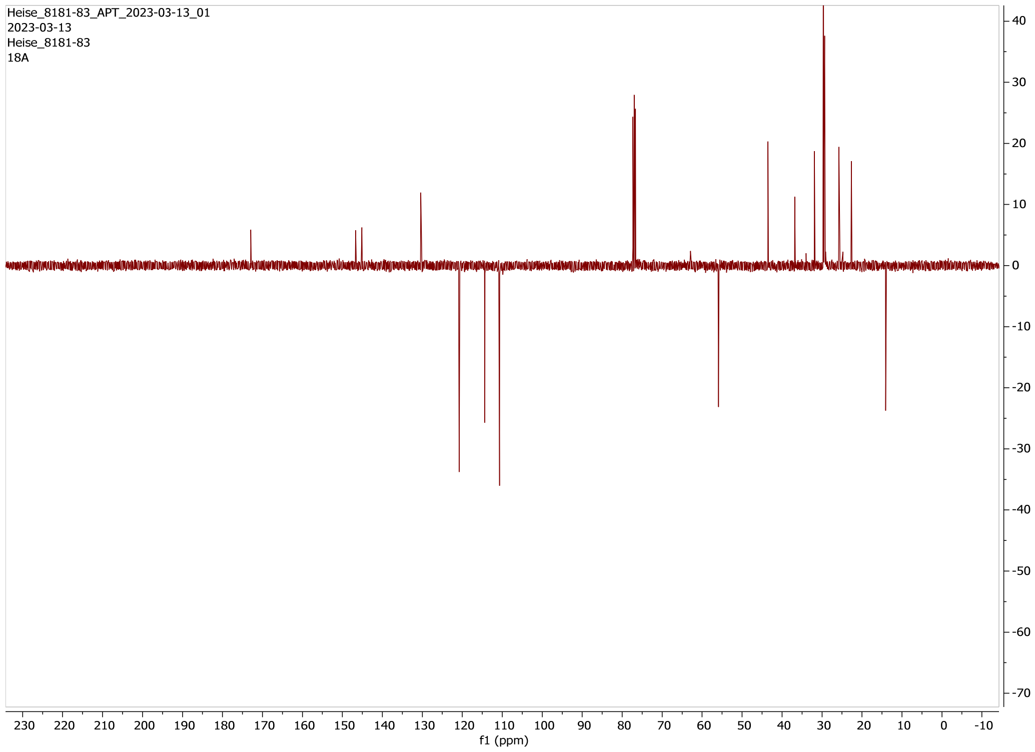

Compound **26**

^{1}H NMR Spectrum ($CDCl_3$, 400 MHz)

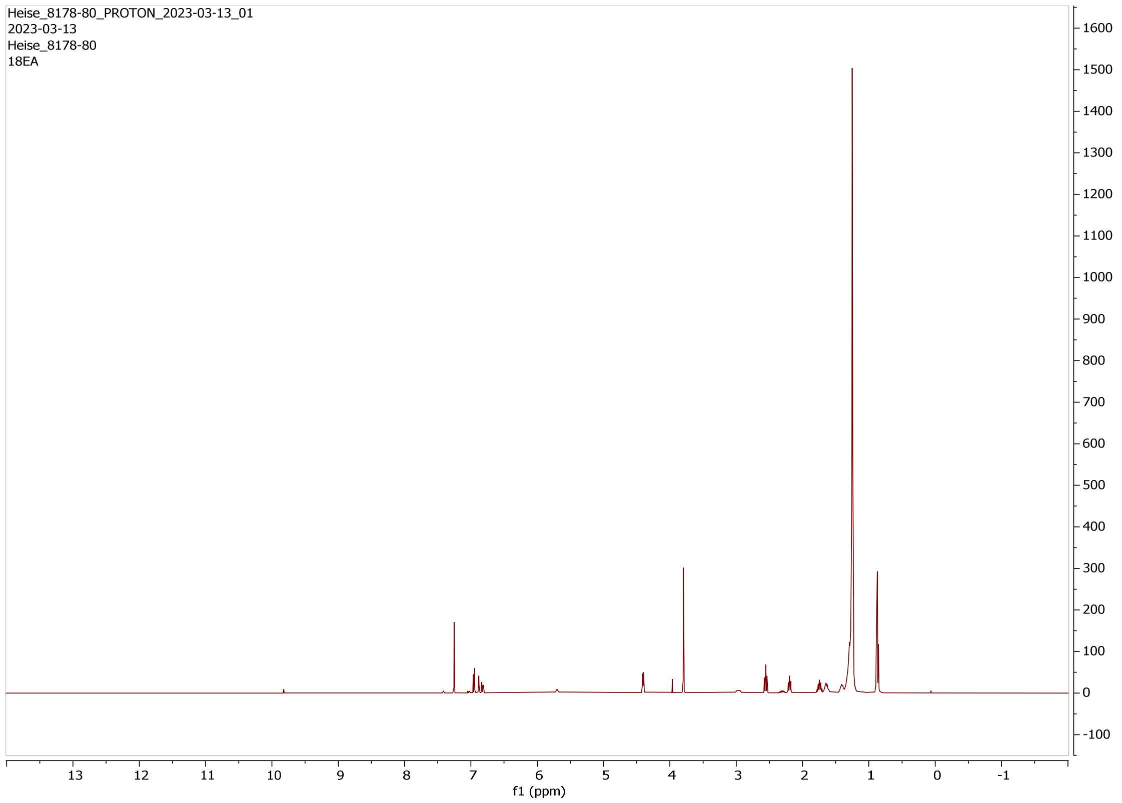

^{13}C-APT NMR Spectrum ($CDCl_3$, 101 MHz)

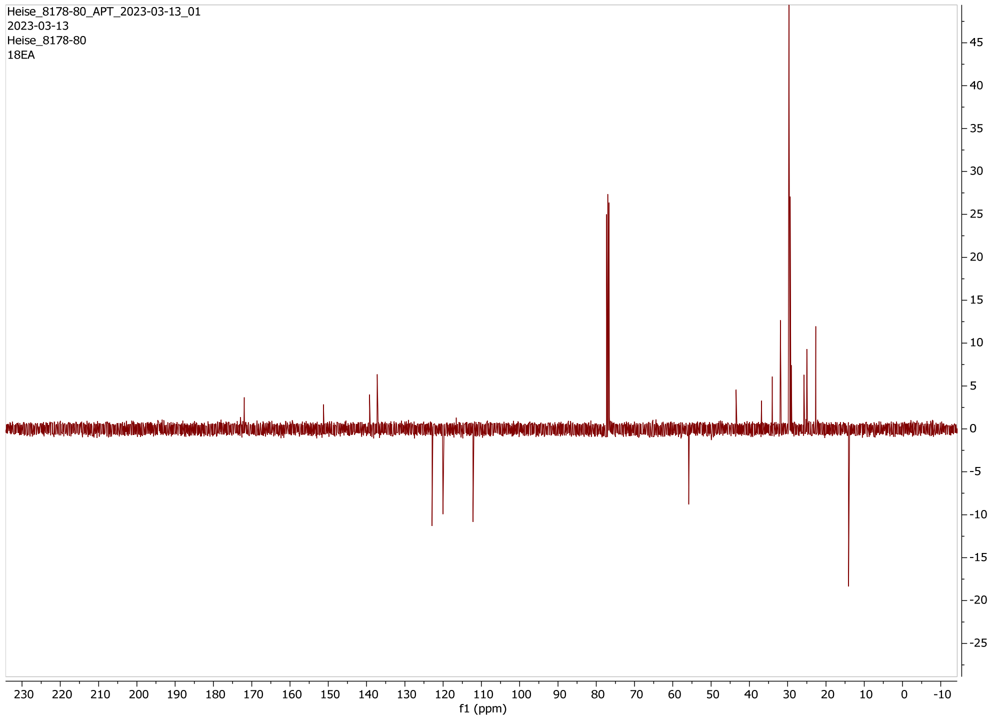

Compound **27**

^{1}H NMR Spectrum ($CDCl_3$, 400 MHz)

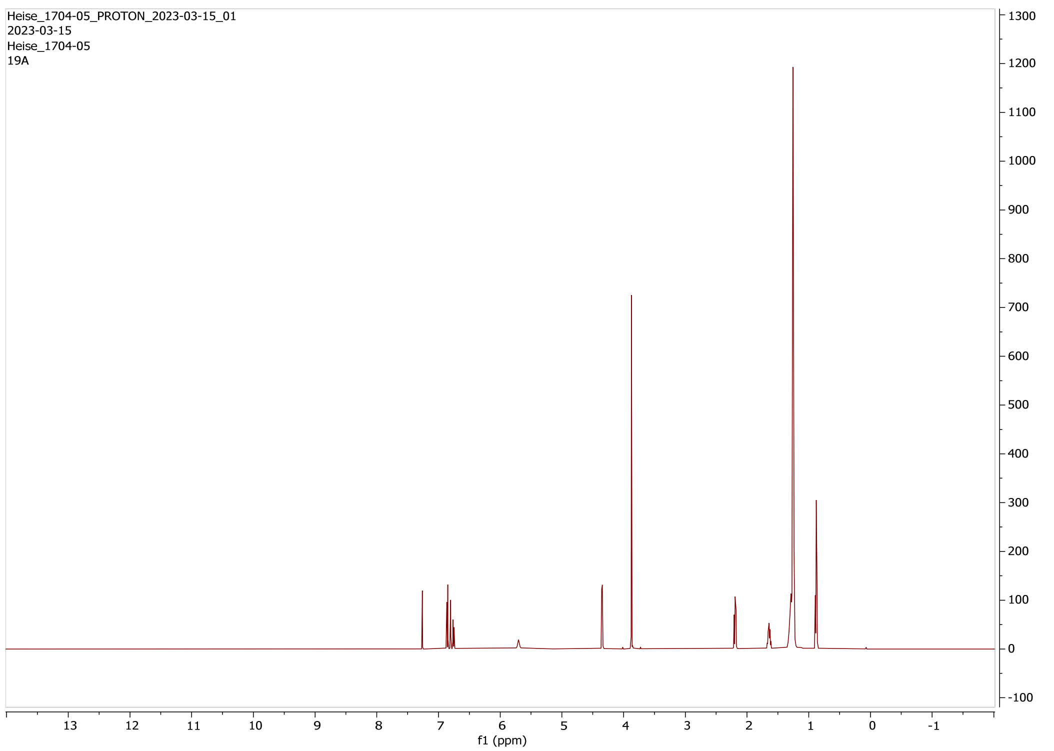

^{13}C-APT NMR Spectrum ($CDCl_3$, 101 MHz)

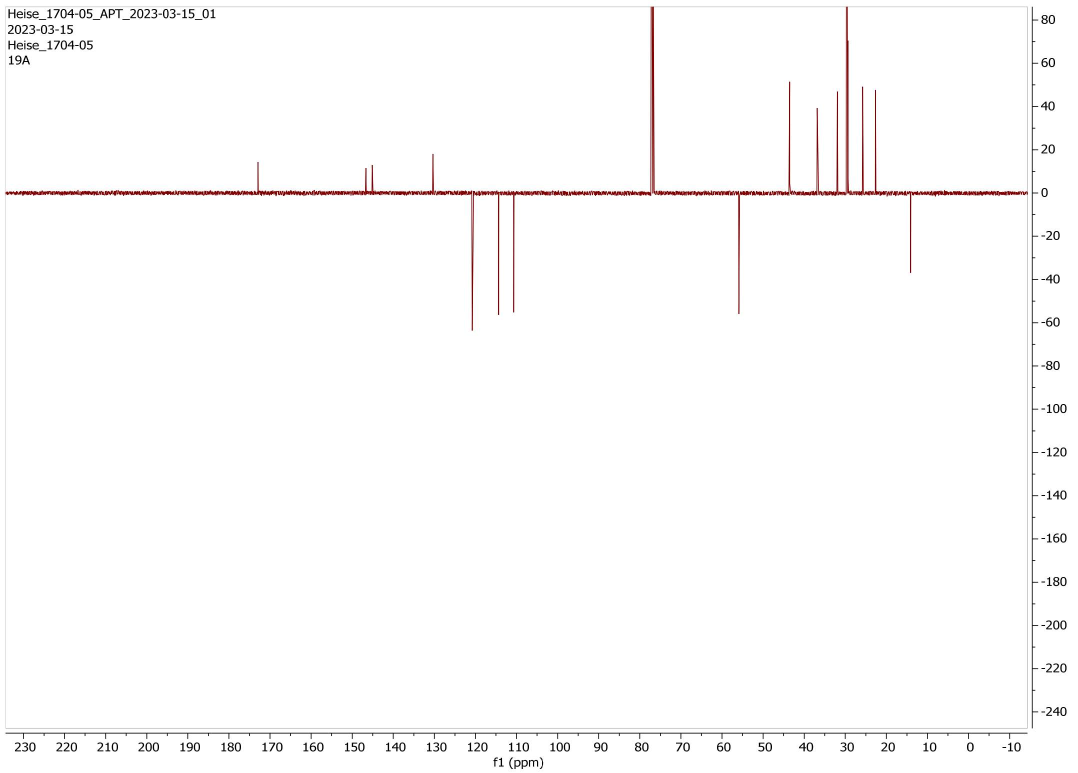

Compound **28**

^{1}H NMR Spectrum (CDCl$_3$, 400 MHz)

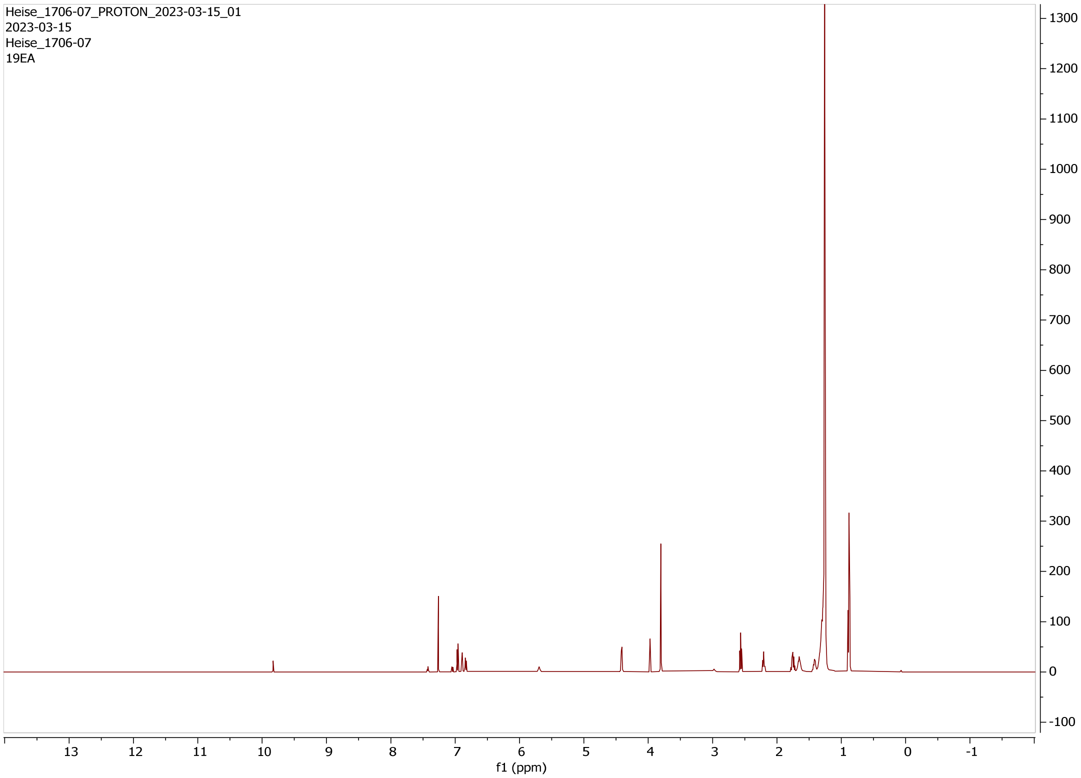

^{13}C-APT NMR Spectrum (CDCl$_3$, 101 MHz)

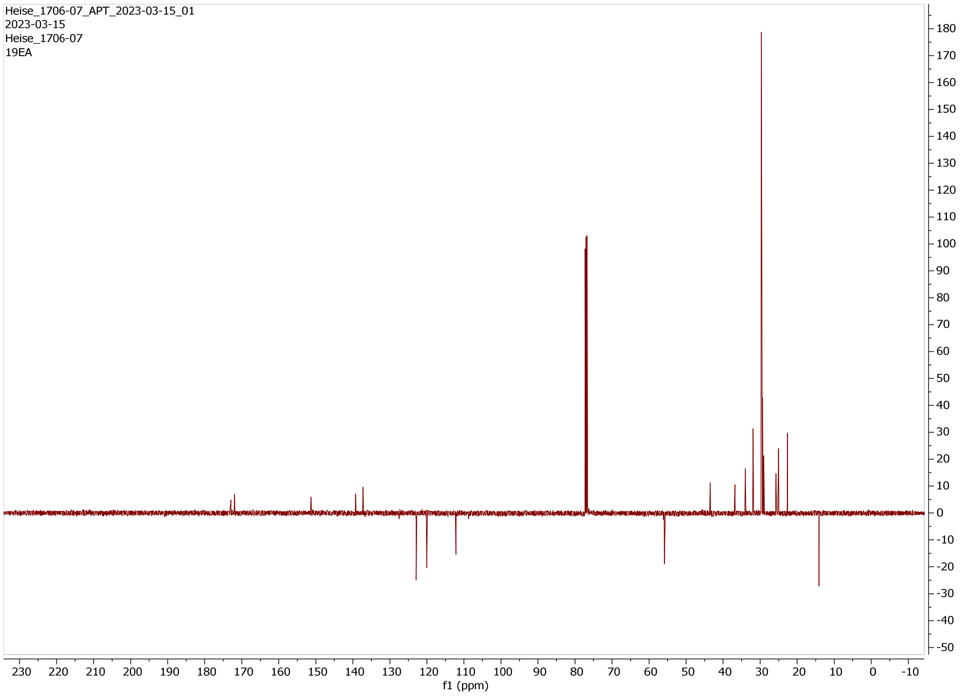

Compound **29**

^{1}H NMR Spectrum (CDCl$_3$, 400 MHz)

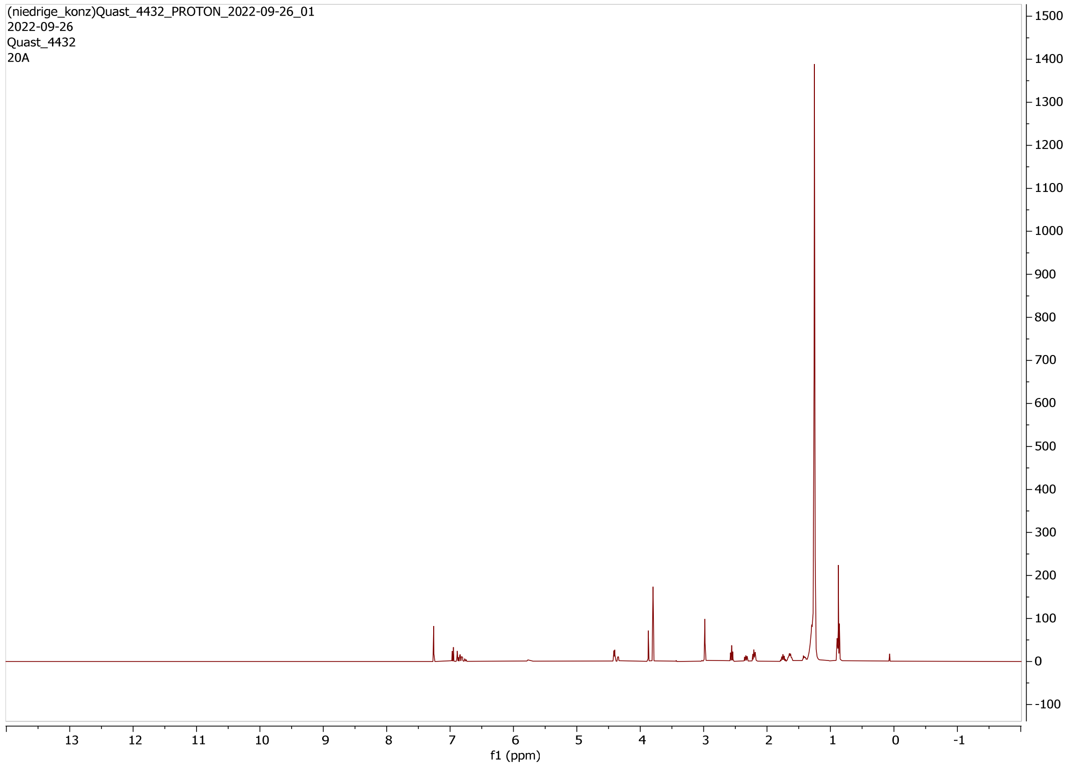

^{13}C-APT NMR Spectrum (CDCl$_3$, 101 MHz)

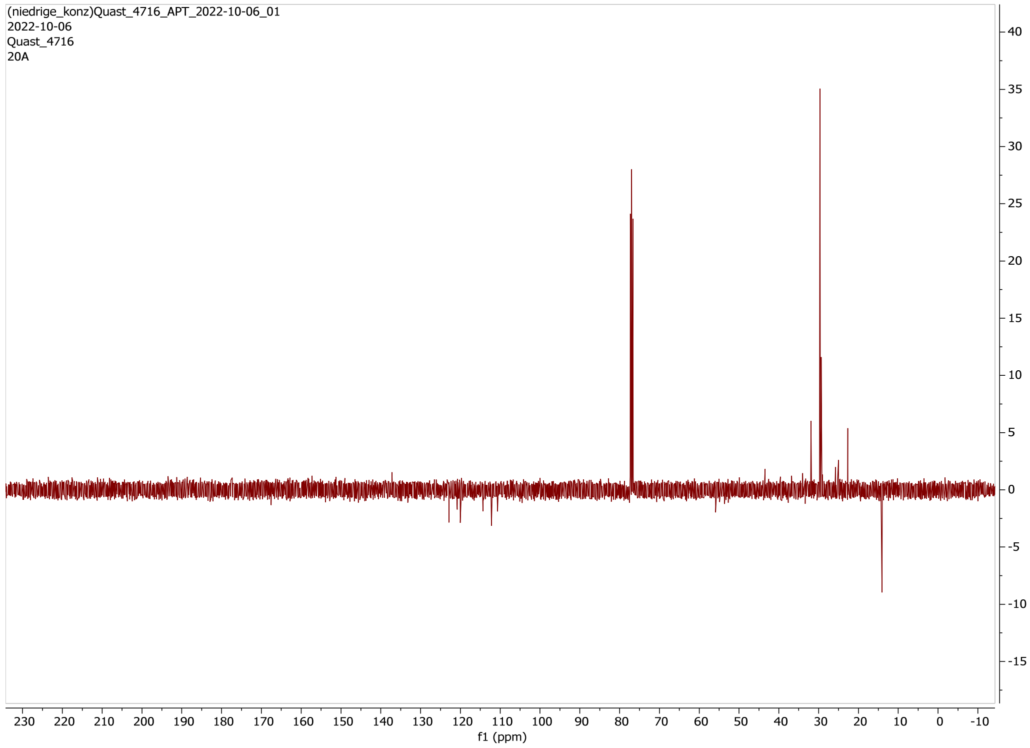

Compound **30**

^{1}H NMR Spectrum ($CDCl_3$, 400 MHz)

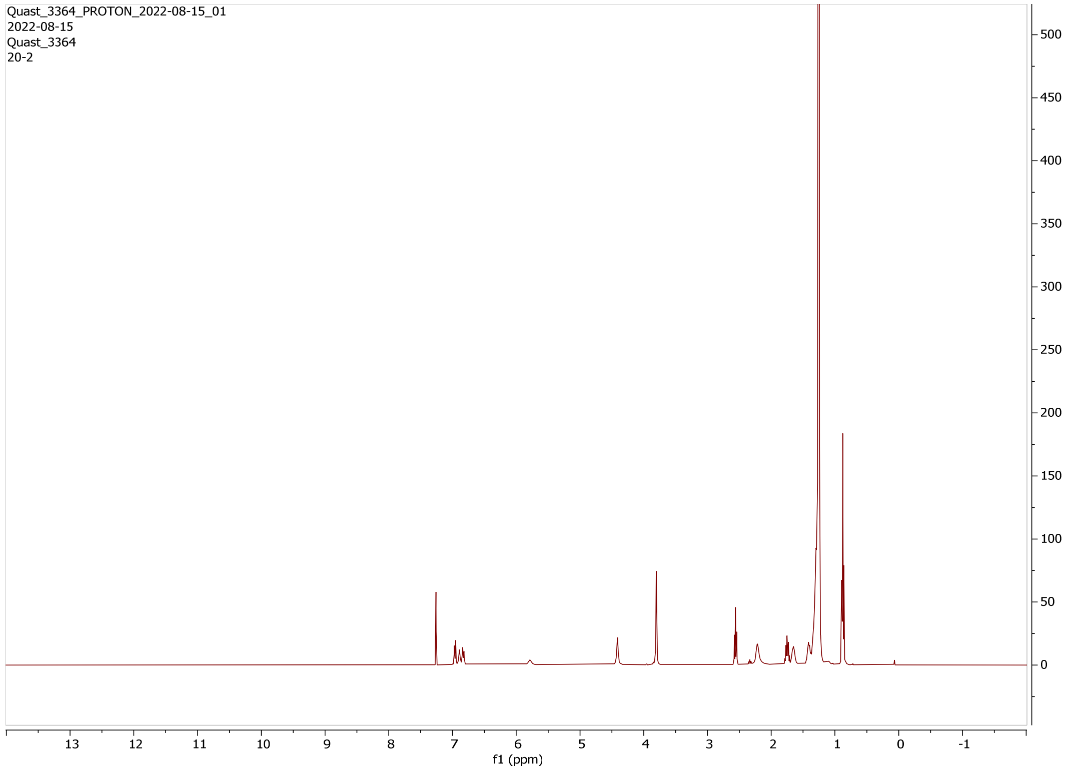

^{13}C-APT NMR Spectrum ($CDCl_3$, 101 MHz)

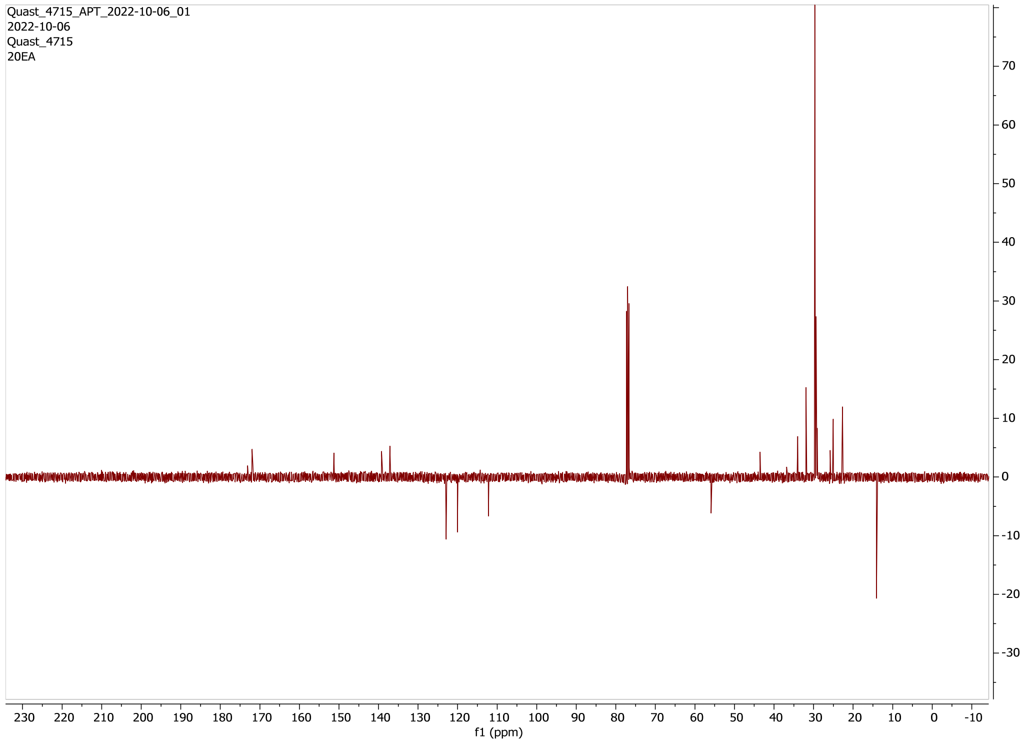

Compound **33**

^{1}H NMR Spectrum ($CDCl_3$, 400 MHz)

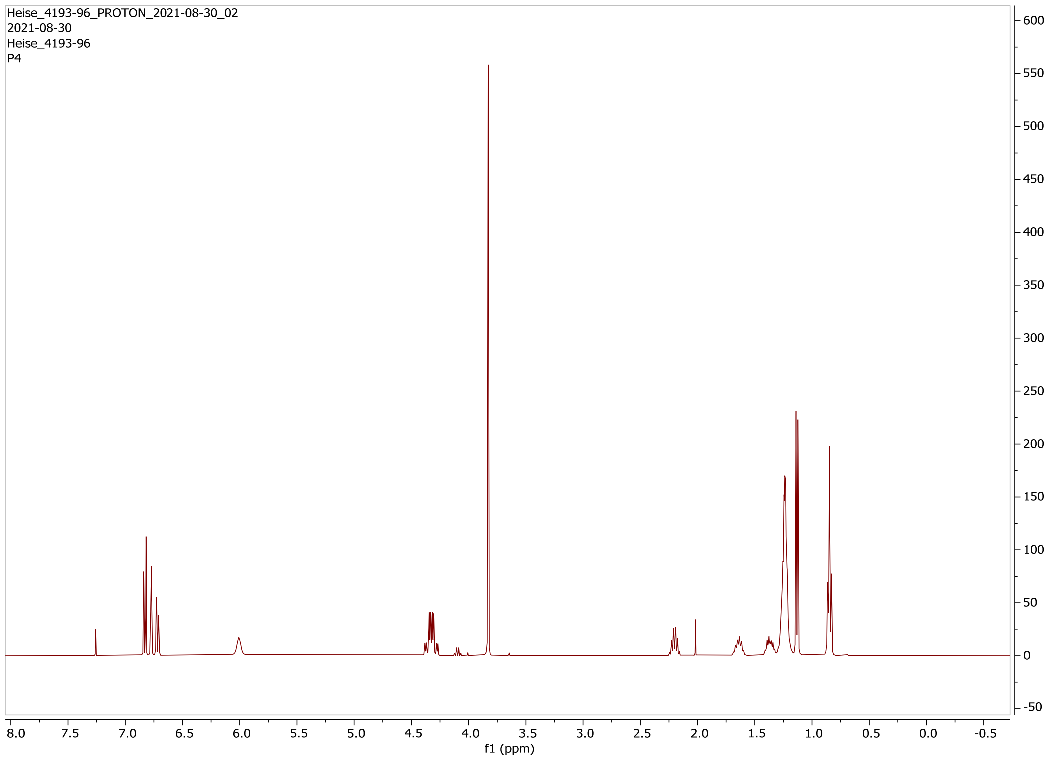

^{13}C NMR Spectrum ($CDCl_3$, 101 MHz)

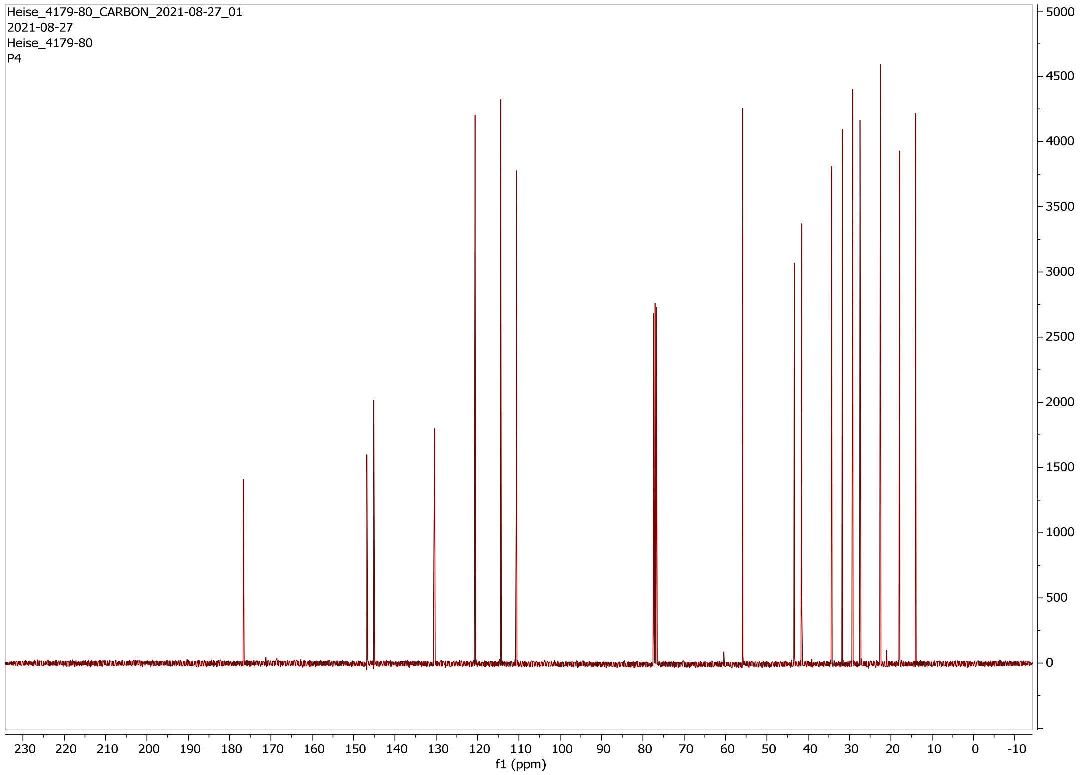

Compound **34**

^{1}H NMR Spectrum ($CDCl_3$, 400 MHz)

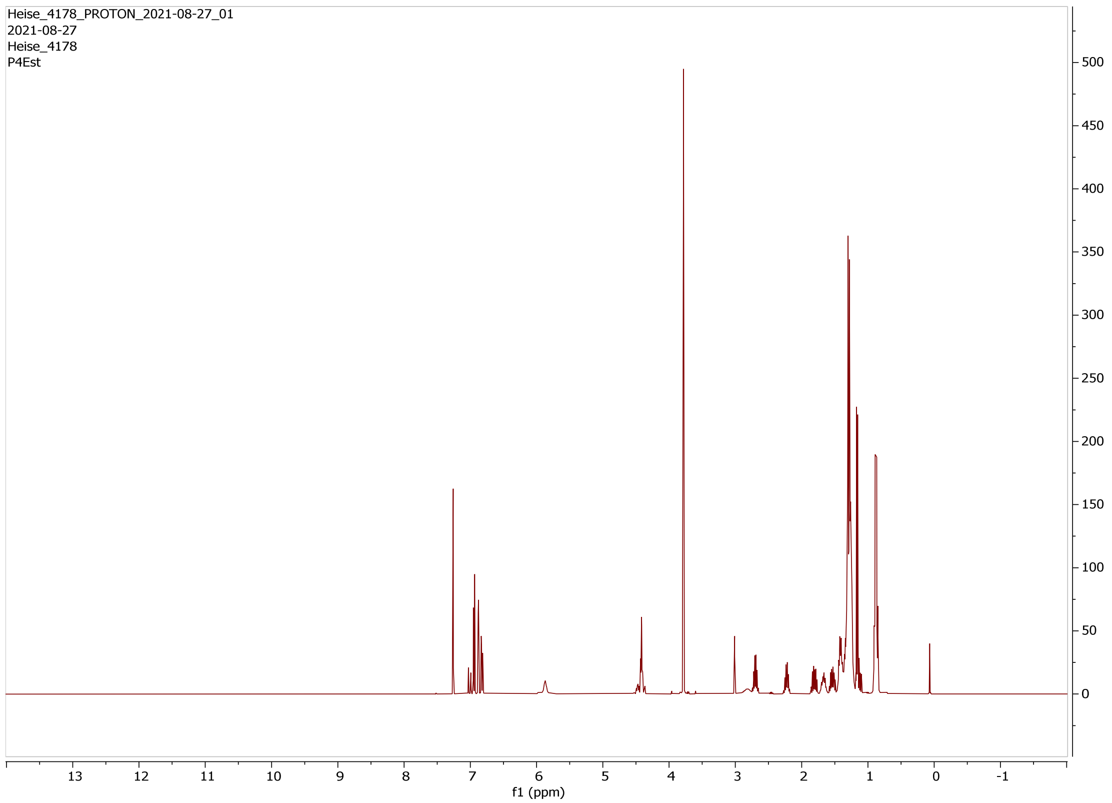

^{13}C NMR Spectrum ($CDCl_3$, 101 MHz)

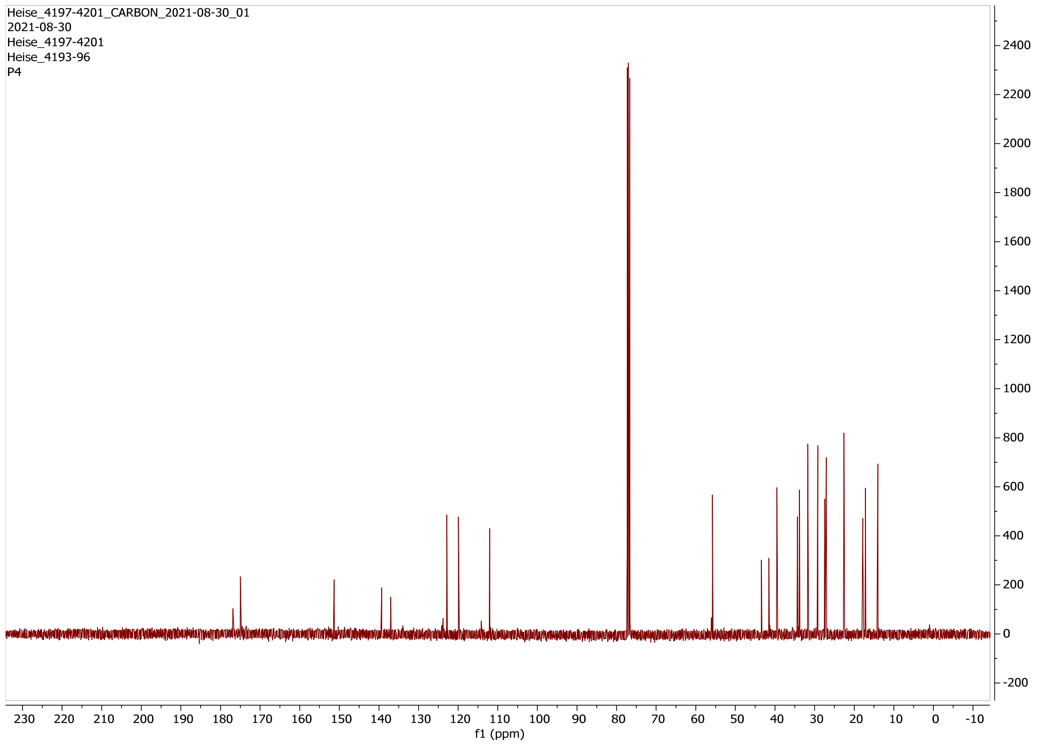

Compound **35**

^{1}H NMR Spectrum ($CDCl_3$, 400 MHz)

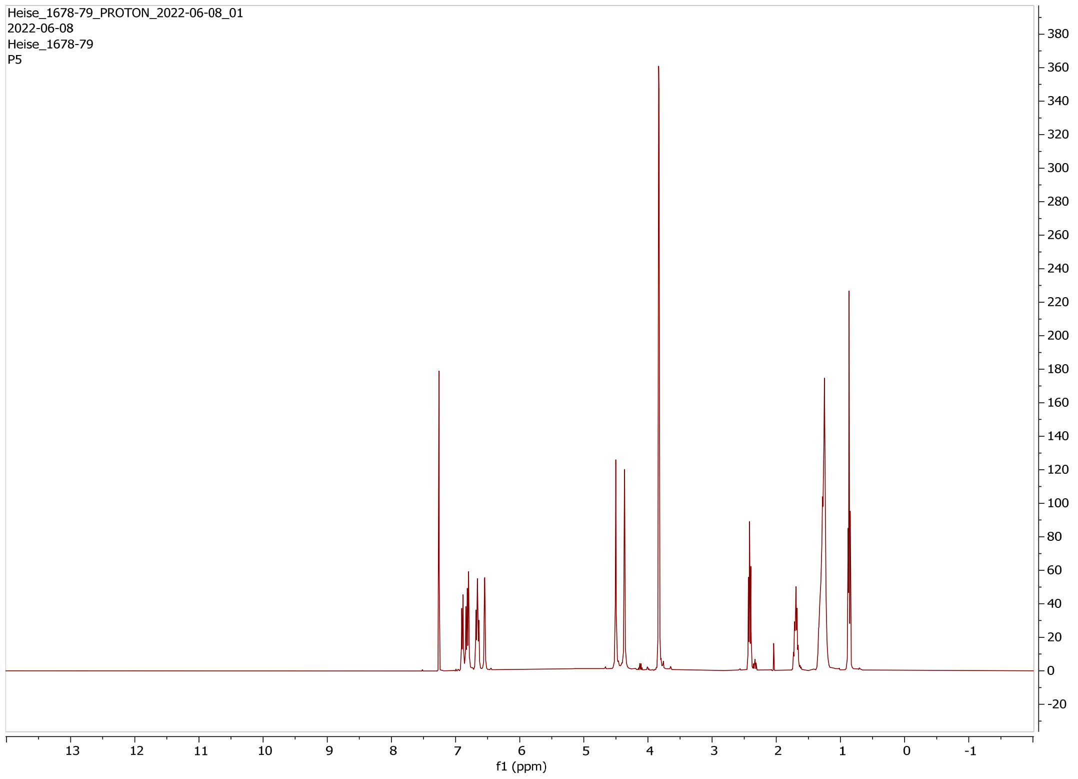

^{13}C NMR Spectrum ($CDCl_3$, 101 MHz)

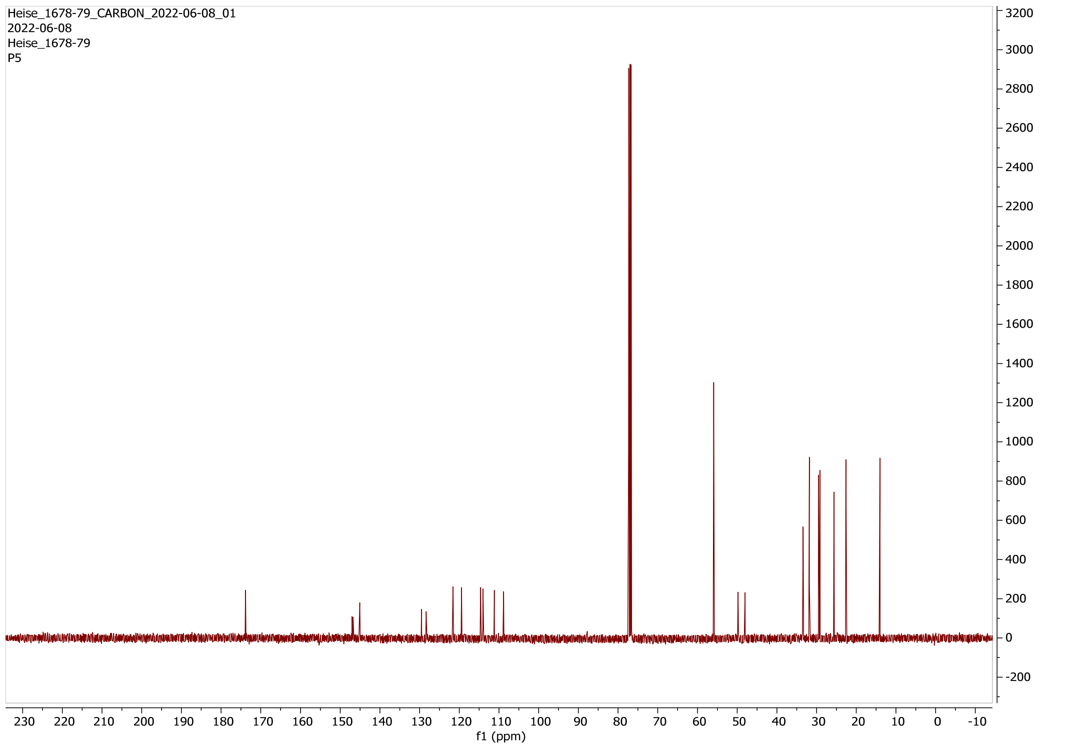

Compound **36**

^{1}H NMR Spectrum ($CDCl_3$, 400 MHz)

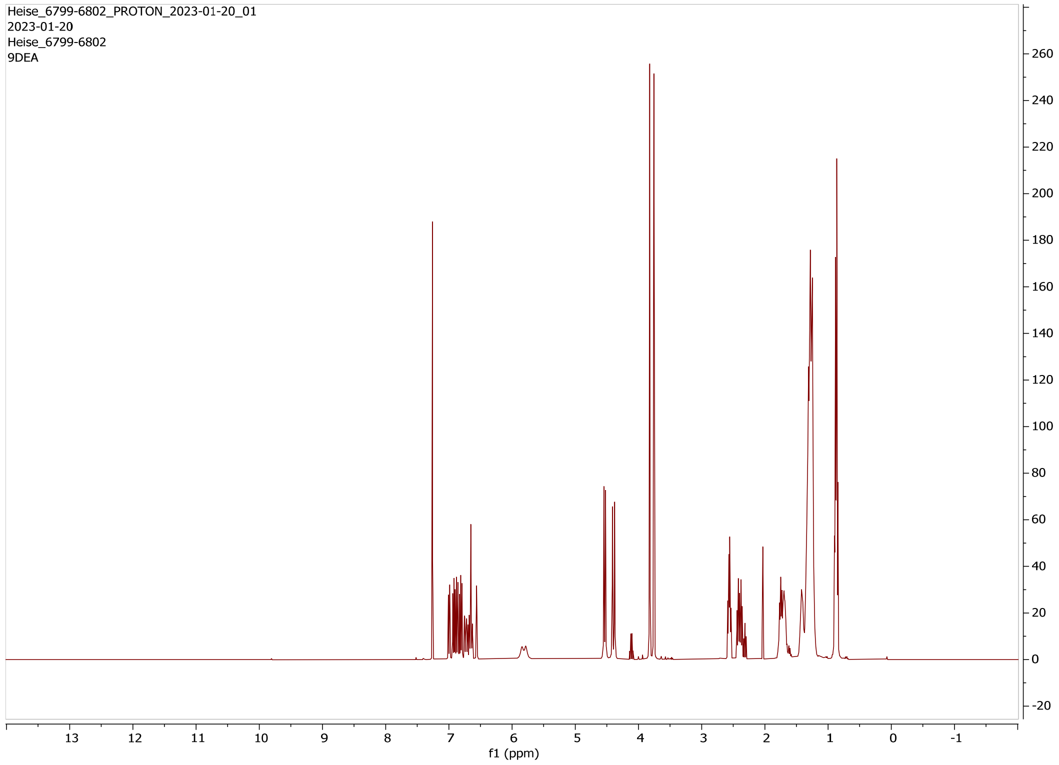

^{13}C-APT NMR Spectrum ($CDCl_3$, 101 MHz)

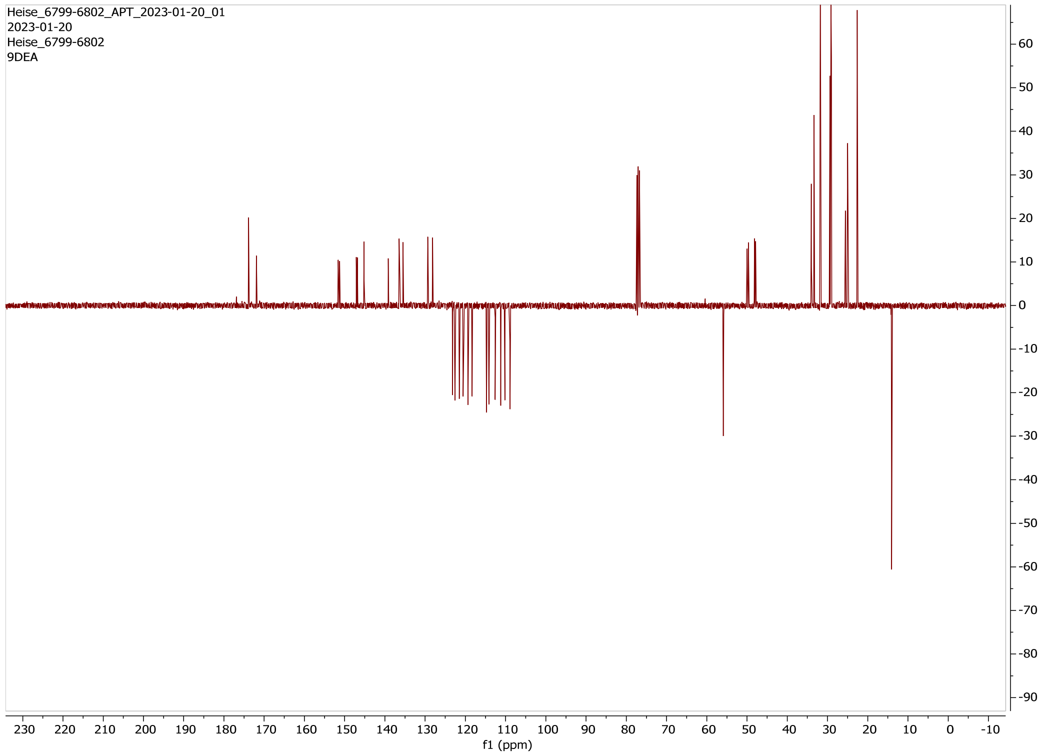

Compound **37**

^{1}H NMR Spectrum ($CDCl_3$, 400 MHz)

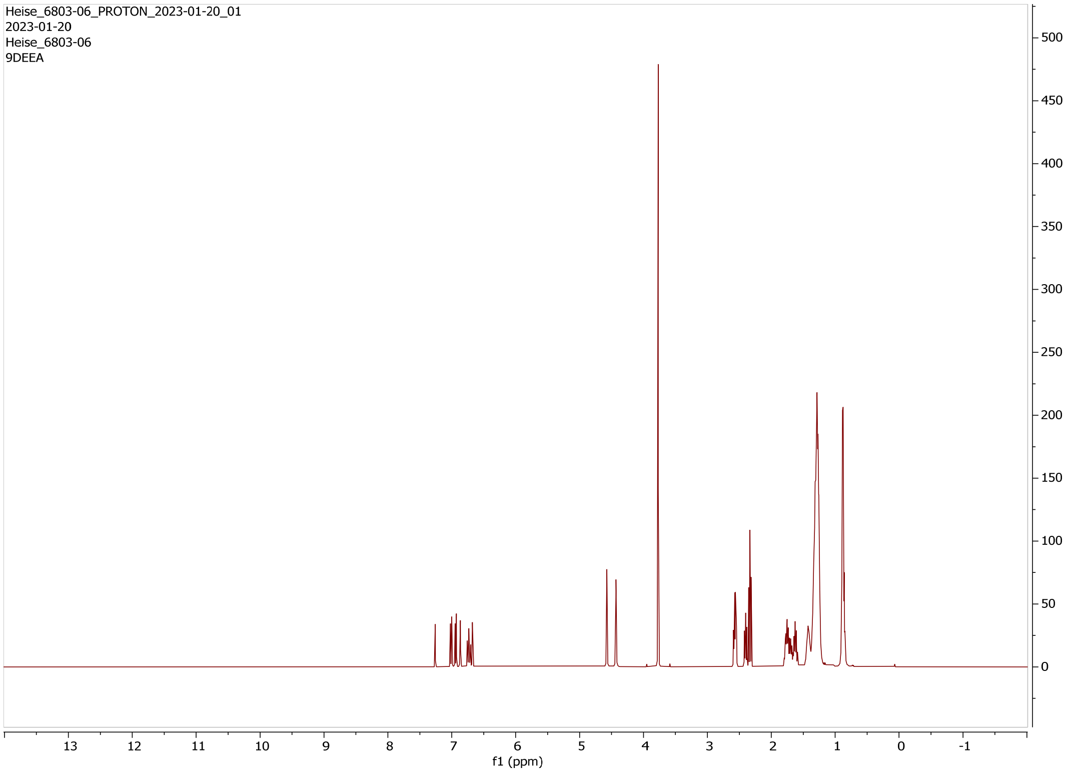

^{13}C-APT NMR Spectrum ($CDCl_3$, 101 MHz)

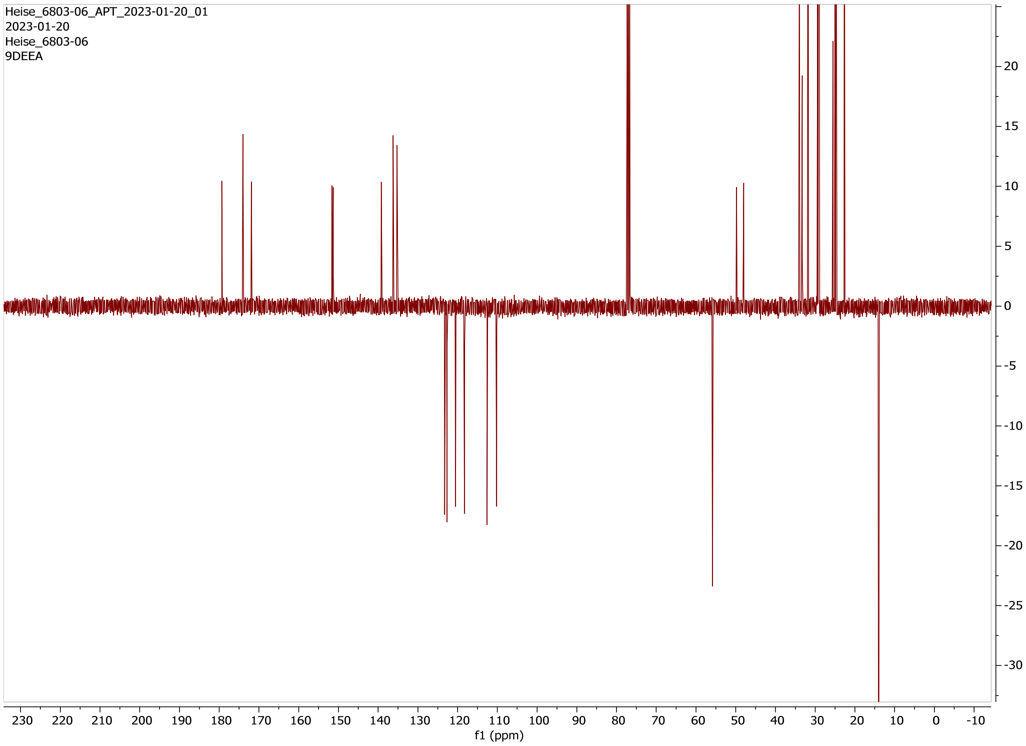

Supplement: Supplementary file 1 [file molecules-30-03488-s001.zip › molecules-3764916-supplementary.pdf]
